# Genome-wide Expression Analysis and Metabolite Profiling Elucidate Transcriptional Regulation of Flavonoid Biosynthesis and Modulation under Abiotic Stresses in Banana

**DOI:** 10.1038/srep31361

**Published:** 2016-08-19

**Authors:** Ashutosh Pandey, Anshu Alok, Deepika Lakhwani, Jagdeep Singh, Mehar H. Asif, Prabodh K. Trivedi

**Affiliations:** 1 CSIR-National Botanical Research Institute, Council of Scientific and Industrial Research (CSIR-NBRI), Rana Pratap Marg, Lucknow 226001, INDIA; 2 National Agri-Food Biotechnology Institute (NABI), Department of Biotechnology, Government of India, C-127, Industrial Area, Phase VIII, S.A.S. Nagar, Mohali 160071, India

## Abstract

Flavonoid biosynthesis is largely regulated at the transcriptional level due to the modulated expression of genes related to the phenylpropanoid pathway in plants. Although accumulation of different flavonoids has been reported in banana, a staple fruit crop, no detailed information is available on regulation of the biosynthesis in this important plant. We carried out genome-wide analysis of banana (*Musa acuminata*, AAA genome) and identified 28 genes belonging to 9 gene families associated with flavonoid biosynthesis. Expression analysis suggested spatial and temporal regulation of the identified genes in different tissues of banana. Analysis revealed enhanced expression of genes related to flavonol and proanthocyanidin (PA) biosynthesis in peel and pulp at the early developmental stages of fruit. Genes involved in anthocyanin biosynthesis were highly expressed during banana fruit ripening. In general, higher accumulation of metabolites was observed in the peel as compared to pulp tissue. A correlation between expression of genes and metabolite content was observed at the early stage of fruit development. Furthermore, this study also suggests regulation of flavonoid biosynthesis, at transcriptional level, under light and dark exposures as well as methyl jasmonate (MJ) treatment in banana.

Secondary plant products are beneficial for human health and utilized as anti-cancerous, anti-inflammatory, immunosuppressive, immunostimulant, anti-osteoporosis and in many other diseases including cardiovascular diseases^1^,^2^,^3^,^4^. Out of the different groups of secondary plant products, flavonoids are the low molecular weight polyphenolic secondary metabolites produced via phenylpropanoid/flavonoid pathway^5^,^6^,^7^. These are further classified into different groups including flavones, flavonols, flavanones, flavan-3-ols and anthocyanidins^8^. The basic skeleton of different groups of flavonoids is further modified by the various enzymes such as glycosyltransferases, acyltransferases, and methyltransferases as well as polymerization leading to huge diversity. To date, more than 9000 different flavonoids have been identified from different plant species.

Due to the selective and differential synthesis of flavonoids, biological activities differ across plant species as well as in different tissues of a plant. These differences have been correlated with the tight spatial and temporal regulation of genes involved in the biosynthesis of different flavonoids. Therefore, a number of food products consumed by the human are deficient in these phytochemicals. Accumulation of flavonoids in plants also fluctuates depending upon cellular, climatic and developmental conditions. These factors are major limitation for proper exploitation of secondary plant products in the drug as well as neutraceutical development.

Out of different biosynthetic pathways leading to secondary plant product biosynthesis, the phenylpropanoid pathway (Fig. 1) is probably the best characterized in terms of enzymatic steps and regulatory genes. Though phenylalanine ammonia lyase (PAL) is the first enzyme of the phenylpropanoid pathway, chalcone synthase (CHS) is the entry step enzyme that is responsible for the biosynthesis of flavonoid backbone (naringenin-chalcone)^5^,^6^,^9^. Naringenin chalcone is enzymatically converted into flavanones through chalcone isomerase (CHI)^10^. Subsequently, flavonoid 3′-hydroxylase (F3′H) catalyses introduction of the −OH group at 3′ position. Another enzyme flavonoid 3′5′hydroxylase (F3′5′H) is responsible for the hydroxylation at 3′ and 5′ positions of the B-ring (Grotewold, 2006). Flavonol synthase (FLS) converts different dihydro-flavonols into corresponding flavonols like quercetin, kaempferol, and myricetin^11^. Dihydroxyflavonol 4-reductase (DFR) is an entry step enzyme for the anthocyanin biosynthesis that acts over dihydroflavonols to produce corresponding leucoanthocyanidins^5^,^12^. Through the aid of enzymes anthocyanidin synthase/leucoanthocyanidin dioxygenase (ANS/LDOX), the leucoanthocyanidins (e.g. leucocyanidin, leucopelargonidin, and leucodelphinidin) are converted into corresponding anthocyanidins (e.g. cyanidin, pelargonidin and delphinidin)^13^,^14^. Leucoanthocyanidin reductase (LAR) converts leucoanthocyanidines into corresponding flavan-3-ols (e.g., catechin, gallocatechin, afzelechin) with 2, 3-trans stereochemistry^15^.

Banana is a staple fruit crop for a major world population, especially in developing countries. Certain agronomic traits such as fruit quality and stress resistance are of great importance in this plant species. For various reasons, banana improvement through breeding exercises has been challenging. Therefore, genetic engineering based manipulations hold great promise for the crop improvement in banana. The recent release of banana genome sequence provides a useful resource for functional genomics and identification of candidate genes which can be utilized in banana improvement program for agronomically important traits^16^. Despite the availability of comprehensive genome resource of banana^17^, there have been limited reports related to genome-wide identification of members from important gene families. To the best of our knowledge, as of now, exhaustive analysis of gene families in banana has been limited to MAPK^18^, ethylene biosynthesis and perception^19^, NAC domain transcription factors^20^, AP2/ERF transcription factors^21^, HDZIV transcription factors^22^ and WRKY transcription factors^23^. In the present work, we have carried out genome-wide identification of gene families involved in the flavonoid biosynthesis in banana. In addition, correlation between expression of specific gene family member with various metabolites in different tissues and throughout the fruit development as well as during various abiotic stresses has been established.

## Results

### Gene families involved in flavonoid biosynthesis in banana

Structural genes encoding enzymes involved in various intermediate steps of the flavonoid biosynthesis pathway were identified through BLAST searches in banana genome using protein from different plant species as query sequences. Protein sequences identified from banana genome were analyzed through multiple sequence alignments with other known proteins. Analysis revealed that some of the sequences were partial with missing regions of variable lengths suggesting requirement of further refinement in the gene models. To achieve this, corresponding genomic sequences were retrieved, and gene models were modified using online tool (Fgenesh program) followed by manual analysis of the intron–exon boundaries. This analysis led to the identification of a total of 28 genes encoding enzymes involved in flavonoid biosynthesis pathway in banana (Supplementary File S1). These putative banana proteins were further divided into five groups including chalcone synthase (CHS), chalcone isomerase (CHI), 2-oxoglutarate-dependent dioxygenase (FLS, F3H, and ANS), CYP450 (F3′H and F3′5′H) and reductase/epimerase superfamily (LAR, ANR, and DFR). The genes, their accession numbers, predicted polypeptide lengths, theoretical isoelectric points (pI), molecular weights, transmembrane helix (TMH) numbers and duplication events are summarized in Table 1.

### Phylogenetic analysis of banana proteins

**Figure 2:**
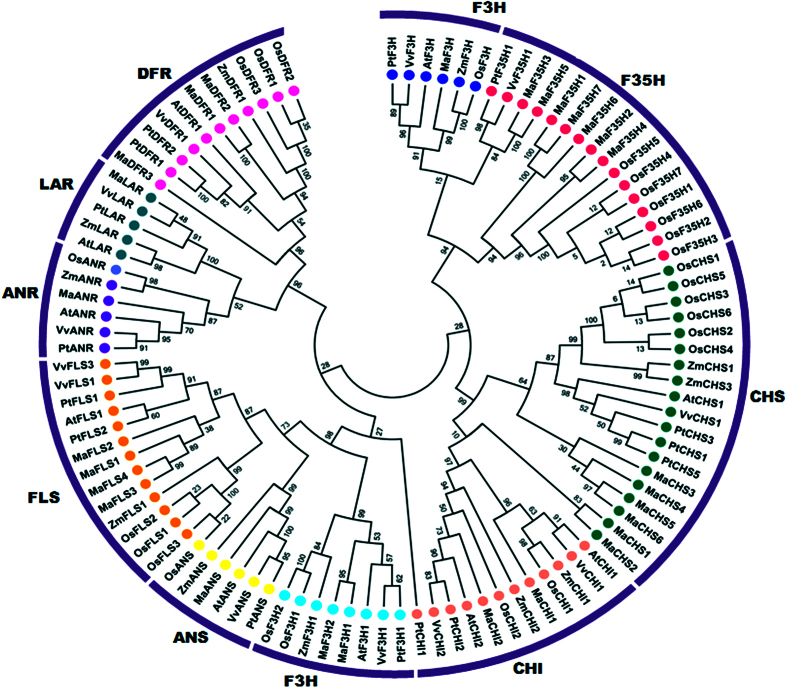
Phylogenetic relationships between genes involved in the flavonoid biosynthesis from different plant species. The Neighbor-joining tree was constructed using MEGA 6 with 1000 bootstrap value.

A phylogenetic tree was constructed to study the relationship of various enzymes of the flavonoid biosynthesis pathway from the banana with the corresponding genes from other plants (rice, maize, and Arabidopsis). Most of the proteins from banana and other monocots grouped together and formed monocot-specific clusters in their respective clades (Fig. 2). In certain groups, there were more paralogs for banana genes (for example *MaF3*′*5*′*H*) as compared to that in other monocots.

### Gene structure, chromosomal location and gene duplication

To study gene organization, genomic regions of the identified genes were analyzed for the architecture of introns and exons. Analysis suggested variation in number of exons and introns in different gene families (Supplementary Fig. S1). However, most of the genes showed exon-intron composition similar to orthologous genes in different species. All the identified genes, except *MaF3'5*′*H7* and *MaLAR*, could be mapped on different banana chromosomes. *MaF3'5*′*H7* and *MaLAR* got mapped on the un_random chromosome in the banana genome (Supplementary Fig. S2). None of the banana genes involved in the flavonoid biosynthesis mapped to the chromosome 1.

### Tissue-specific gene expression

**Figure 3:**
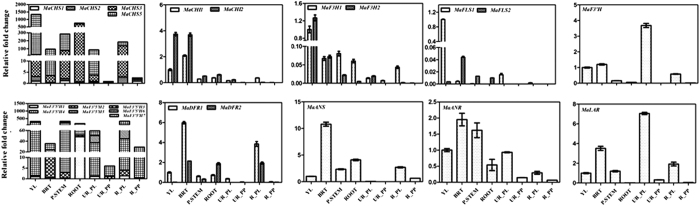
Expression analysis of genes in various tissues. Different tissues are the Young leaf (YL), Bract (BT), Pseudostem (P. STEM), Root (ROOT), Unripe peel (UR-PL), Unripe pulp (UR-PP), Ripe peel (R-PL) and Ripe pulp (R-PP). Expression of genes related to flavonoid biosynthetic pathway was analyzed using Real-Time PCR and RNA from different tissues of banana. In each case, expression level is expressed as relative fold change as compared to the young leaves tissue. The graph shows values ± SD of three samples from each of the independent plant.

**Figure 4:**
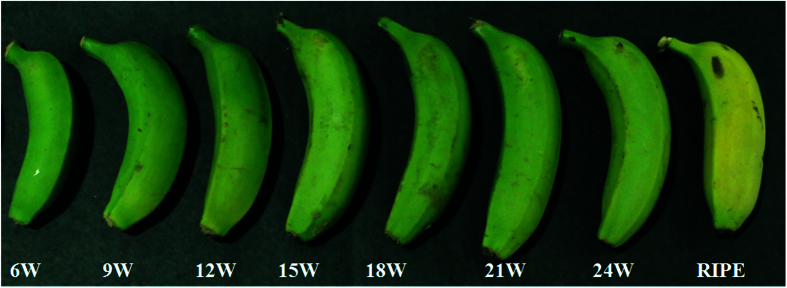
Different developmental stages of banana fruit used for molecular and phytochemical analysis. 6W, 9W, 12W…… upto 24W (full green stage) represents number of weeks post bunch emergence. After full green stage (24W), ripening was induced by ethylene treatment (100 μL/L) for 24 h at 22 °C in dark and then allowed to ripen in the air.

Most of the identified genes expressed differentially in tissues used in the study. Surprisingly, transcripts of *MaCHS4, MaFLS3* and *MaDFR3* were not detected in any of the tissue used for expression analysis. Higher expression of most of the genes, except F3’H, DFR and LAR, was observed in vegetative tissues as compared to fruit tissues (Fig. 3). To study the transcriptional regulation of flavonoid biosynthesis in banana, the transcript profiling of the identified flavonoid biosynthesis genes were carried out in the different developmental stages of the fruit (from the onset of flowering to ripening) at the 3-week intervals separately in pulp and peel tissues (Fig. 4). Results observed for each gene family are provided below in different sections.

### Chalcone synthase gene family

*In silico* analysis identified six homologs of chalcone synthase in banana genome. Out of six CHS homologs, transcripts of four genes were detected in different tissues at varying level. Out of all the expressing CHS homologs, minimum expression was observed for the *MaCHS1* in all the tissue except in fruit pulp. The transcript of *MaCHS2, MaCHS3* and *MaCHS5* was highly abundant in different tissues. The transcript of *MaCHS5* was maximum in the young leaves. The expression of *MaCHS2* and *MaCHS3* was maximum in root followed by in the peel of ripe fruit (Fig. 3).

**Figure 5:**
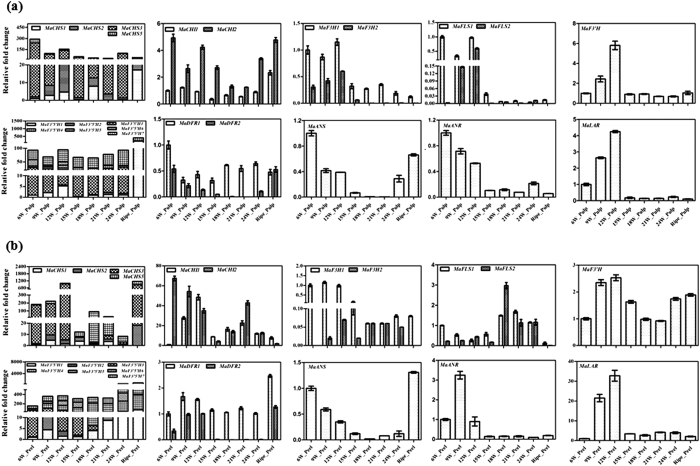
Expression analysis of genes at different developmental stages of fruit. Expression of genes related to flavonoid biosynthetic pathway was analyzed by real time PCR using RNA from different developmental stages of banana fruit pulp (**a**) and peel (**b**). In each case, expression level is expressed as relative fold change as compared to the 6W old tissue. The graph shows values ± SD of three samples from each of the independent fruit.

In case of pulp of banana fruit, the maximum expression was observed for *MaCHS1* and *MaCHS2* at the ripening stage whereas the transcript of *MaCHS3* was highly abundant at the unripe stage of the fruit pulp (Fig. 5a). In the peel tissue, the maximum expression of *MaCHS2, MaCHS3* and *MaCHS5* was observed in the ripe fruit as compared to the peel of fruits from other developmental stages (Fig. 5b).

### Chalcone isomerase gene family

*In silico* analysis revealed presence of two members of CHI in the banana genome. The expression of both the genes was significantly higher in young leaves and bract as compared to other tissues of banana (Fig. 3). Differential expression of both the genes was observed throughout the fruit developmental stages. In case of fruit pulp, expression of *MaCHI1* and *MaCHI2* was higher in the pulp of fruit at the ripening stage (Fig. 5a). In contrast, expression of both the genes was higher in the peel tissue of unripe fruit as compared to peel of the ripe fruit (Fig. 5b).

### 2-Oxoglutarate-dependent dioxygenase (F3H, FLS and ANS) gene family

Our *in silico* analysis suggests presence of two homologs of F3H in the banana genome. Both the genes showed differential expression in tissues analysed in this study. The transcripts of both the genes were abundant in the leaf tissue while minimum expression was found in the pulp of ripe fruit (Fig. 3). Similar to our result, the higher transcript levels for F3H were reported in the young and growing tissues such as young leaves and flower buds of grape fruit^24^.

The expression analysis of F3H in pulp (Fig. 5a) and peel (Fig. 5b) of different developmental stages of fruit suggested that expression of both the homologs was the maximum in the early stage of fruit pulp which decreased with fruit maturation. In agreement with our result, decrease in the expression of F3H has been reported in bilberries at the onset of fruit ripening^25^,^26^. In the peel tissue, *MaF3H1* followed similar expression pattern as in the case of pulp tissue; however the transcript of *MaF3H2* was not detected in the peel of fruit at the early stage of fruit development (6W) as well as in ripe fruit (Fig. 5b).

Though four homologs of FLS encoding genes were identified in the banana genome, expression of only two was detected in tissues analysed in this study. The multiple copies of FLS encoding genes have also been reported in several other plant species including Arabidopsis^26^,^27^,^28^. The expression of FLS encoding genes was the maximum in the young leaves and bract (Fig. 3). The expression analysis in fruit developmental stages suggested that the transcript of both the homologs was higher in the pulp of fruit at the early developmental stages which decreased with the maturation of the fruit (Fig. 5a). Similar results have been reported in other plant species and it was suggested that flavonols and their glycosides accumulate in the growing tissues which subsequently decrease with maturation^29^,^30^. In contrast to expression in pulp, expression of genes encoding FLS was low in the peel of fruit at the early developmental stage (6W–15W) and increased with fruit maturation (18W–24W) and ceased during ripening (Fig. 5b). These results suggest that members of the *MaFLS* gene family are differentially regulated in different tissues and developmental stages of the fruit. Earlier reports suggest that Arabidopsis FLS have different substrate specificities which controls the amount and type of flavonols synthesised in tissue-specific manner^31^,^32^.

In most of the plant species including Arabidopsis, ANS is encoded by a single copy gene^33^,^34^. Our analysis suggests that ANS in banana is encoded by a single gene. Maximum expression of ANS encoding gene was observed in the bract tissue followed by pseudostem suggesting that anthocyanin biosynthesis pathway may be more active in bract as compared to other tissues (Fig. 3). The expression analysis in different developmental stages suggested that the expression of *MaANS* in both, pulp and peel was higher in the early developmental stages of fruit (6W–12W). The expression decreased with fruit maturation; however, again increased at the time of ripening (Fig. 5a,b). The similar expression pattern of ANS gene was observed in the fruit development analysis of berry fruit^35^,^36^.

### CYP450 (F3′H and F3′5′H) gene family

Hydroxylation at B-ring of flavonoid backbone is catalysed by members of cytochrome P450 family including F3′H, and F3′5′H^37^. Our analysis revealed presence of single gene encoding F3′H in the banana genome. In tissue-specific expression analysis, the highest expression of F3′H was observed in the peel of unripe fruit whereas lowest expression was recorded in the pulp of the ripe fruit (Fig. 3). The expression analysis of *MaF3′H* in fruit developmental stages followed a biphasic pattern (Fig. 5a,b). These results suggest that *MaF3′H* might have consistency of function in both anthocyanin and PA biosynthesis.

F3′5′H catalyses hydroxylation at 3′ and 5′ position of the B ring and leads to the biosynthesis of myricetin- and delphinidin-type of anthocyanin. Interestingly, seven members of F3′5′H gene family are present in the banana genome, and all of them express at varying level in different tissues. Large number of F3′5′H encoding genes in the banana genome suggest that each member may encode different isoforms with varying substrate specificity and may act in a tissue-specific manner. The transcript of *MaF3*′*5*′*H* was highest in the pulp and peel of ripe fruit (Figs 3 and 5). In previous reports, content of myricetin and its derivatives was identified by various groups in banana fruit and sap^38^,^39^. The higher transcript of *MaF3*′*5*′*H* in the ripe stage is in agreement with these report in which the expression of *MaF3*′*5*′*H* was weak at the early developmental stages of fruit which increased during ripening leading to enhanced anthocyanin biosynthesis^36^.

### Reductase epimerase (DFR, ANR, and LAR) gene family

Three homologs of DFR encoding genes were identified in the banana genome; however only two genes expressed in tissues analysed in this study. Though presence of a single copy of DFR is reported in Arabidopsis, rice and grapes however; multiple copies have also been identified in Petunia, Ipomoea, and Medicago^26^. A differential expression of DFR (although not statistically significant) was found throughout the fruit development in peel and pulp tissues (Fig. 5a,b).

Our analysis suggests presence of single copy genes encoding ANR and LAR. The differential expression of these genes was observed in various tissues used in this study (Fig. 3). Expression of both the genes (ANR and LAR) was higher in the peel and pulp of the fruit at the early developmental stage. The expression of these genes was significantly higher in the peel as compared to pulp of the fruit (Fig. 5a,b). The higher transcript levels of ANR and LAR at the early developmental stages may be related to epicatechin- and catechin-type of flavon 3-ols, respectively during fruit development. Similar to our results, higher transcript of PA biosynthesis-specific genes was observed at the early developmental stages of several other fruits^35^,^36^,^40^. These studies reported reduction in expression of ANR and LAR encoding genes however, content of catechin and epicatechin was at a significant amount. Similar observations were also made for the regulation of PA biosynthesis in blueberry fruit. In blueberry, though expression of ANR and LAR encoding genes was significantly reduced in the later stage of ripening, no reduction was observed in PA content. These observations support the hypothesis that PA biosynthesis starts at the early developmental stages of the fruit, after that it may get sequester in the specific cells during fruit maturity.

### Metabolite content analysis

**Figure 6:**
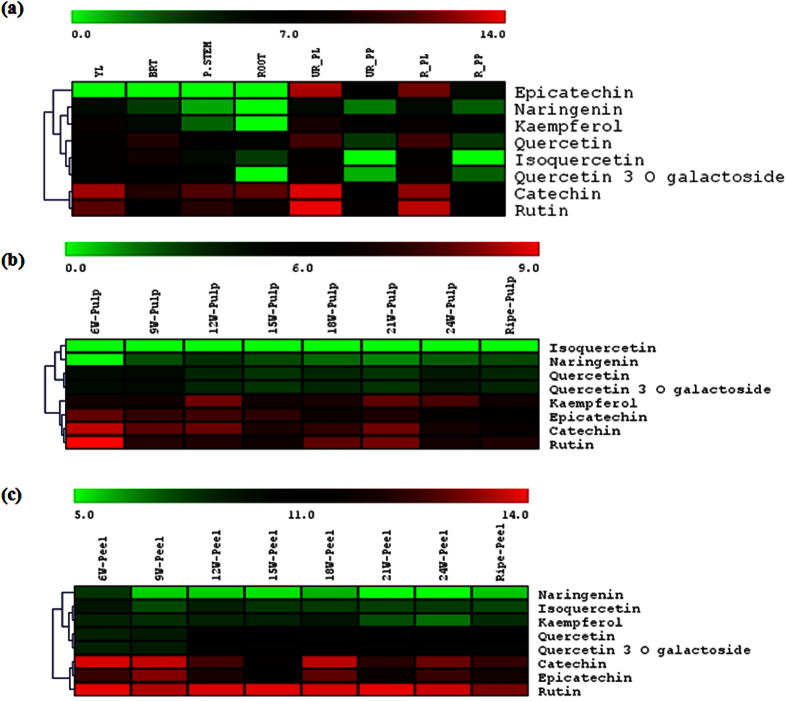
Metabolite analysis in different tissues of banana. Phytochemical analysis of methanolic extracts of different tissues (**a**), fruit pulp (**b**) and peel (**c**) of banana. Metabolites were quantified by separating methanolic extracts using HPLC. Log_2_ transformed values were used for heat map construction. Green to red colour is showing lower to higher expression of genes. Individual values were given in supporting information Table S2.

The quantitative phytochemical analysis of methanolic extracts from different vegetative tissues as well as different developmental stages of fruit (peel and pulp, separately) was carried out using HPLC coupled with Q-TRAP. HPLC analysis identified different classes of flavonoids such as flavanones (Naringenin), flavonols (isoquercetin, quercetin, and kaempferol), flavonol glycoside (quercetin 3-O-galactoside and rutin), and flavan-3-ols (catechin and epicatechin) at varying level in different banana samples. Isoquercetin was identified in all the tissues analyzed except pulp (both unripe and ripe) of the fruit. Content of isoquercetin ranged from 10.28 to 486.00 μg/100 g DW in root and bract tissue, respectively. Quercetin-3-O-galactoside was detected in all the tissue except root and its content ranged from 2.09 to 321.69 μg/100 g DW in unripe pulp and unripe peel, respectively. Rutin was identified as the major flavonoid and its content ranged from 117.30 to 14310.37 μg/100 g DW in bract and unripe peel tissue, respectively. Quercetin and kaempferol was also identified as aglycone form of flavonols. The content of quercetin ranged from 11.08 to 1811.77 μg/100 g DW in ripe and unripe peel tissue, respectively. Similarly, the content of kaempferol ranged from 5.27 to 595.61 μg/100 g DW in pseudostem and unripe peel tissue, respectively (Fig. 6a and Supplementary Table S2).

A detectable amount of naringenin, a flavanone, was also identified in most of the tissues except root sample. Its content ranged from 2.24 to 58.63 μg/100 g DW in pseudostem and unripe peel tissue, respectively. Catechin and epicatechin (flavan-3-ols) were also identified in different tissues. The maximum content of catechin and epicatechin was observed in the unripe peel tissue. The content of catechin ranged from 94.66 to 12742.37 μg/100 g DW in ripe pulp and unripe peel tissues, respectively. Similarly, the content of epicatechin ranged from 51.40 to 7816.92 μg/100 g DW in ripe pulp and unripe peel tissues, respectively (Fig. 6a and Supplementary Table S3 and S4). Modulated content of different classes of flavonoids was observed in pulp and peel of fruit during different developmental stages (Fig. 6b,c). In general, the content of flavonoids decreased in pulp and peel tissue during ripening of banana fruits.

### Expression of genes and flavonoid content under stress conditions

Flavonoid biosynthesis is modulated by various biotic and abiotic stresses^41^. Therefore, to study the effect of various environmental factors including light and defense signals on flavonoid biosynthesis in banana, we carried out expression analysis of flavonoid biosynthesis associated genes as well as metabolite profiling in response to continued incubation in dark as well as methyl jasmonate treatment.

### Dark exposure modulates expression of genes and flavonoid content

**Figure 7:**
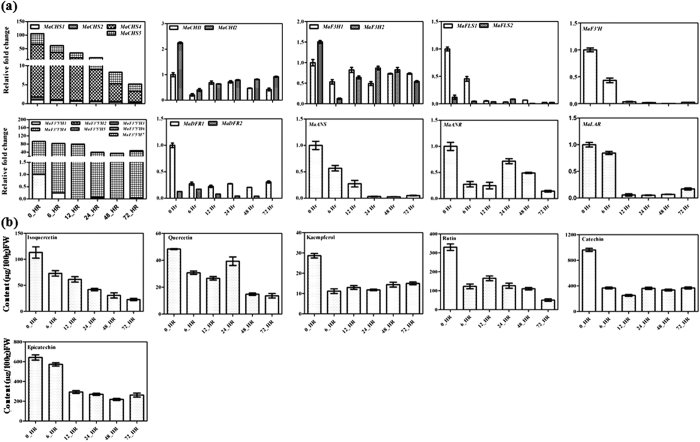
Expression of genes and flavonoid content following dark treatment. (**a**) Expression of genes associated with flavonoid biosynthetic pathway was analyzed by Real-Time PCR using RNA from different time points of dark exposed leaves of banana plantlets. In each case, expression level is expressed as relative fold change as compared to the control leaves (zero hour). (**b**) Phytochemical analysis of methanolic extracts from different time points of dark exposed leaves of banana plantlets. Compounds were quantified by separating methanolic extracts using HPLC. The graph shows values ± SD of three samples from each of the independent plantlets.

Light is one of the most important modulators and prerequisite for the expression of genes involved in flavonoid biosynthetic pathway under normal growth conditions^42^,^43^,^44^. We investigated expression of various genes involved in the flavonoid pathway in banana after dark exposure to the banana plants for 0, 6, 12, 24, 48 and 72 h. The qPCR expression analysis clearly suggests that the dark exposure leads to significantly down-regulated expression of genes involved in the flavonoid biosynthesis (Fig. 7a). As dark exposure modulated expression of genes involved in the flavonoid biosynthesis, we carried out metabolite profiling of these samples. The analysis indicated reduced content of various flavonoids in dark exposed samples (Fig. 7b). Expression of genes and metabolite analyses clearly suggest a positive effect of light over the flavonoid biosynthesis and the involvement of light-regulated factors in the regulation of flavonoid biosynthesis in banana.

### Expression of genes and flavonoid content in response to methyl jasmonate

**Figure 8:**
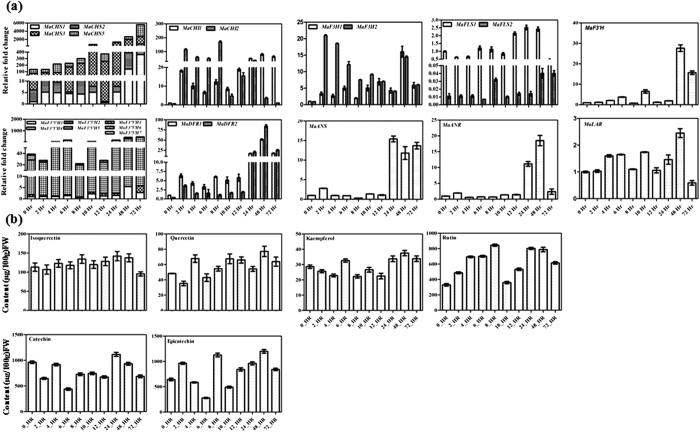
Expression of genes and flavonoid content following methyl jasmonate treatment. (**a**) Expression of genes associated with flavonoid biosynthetic pathway was analyzed by real time PCR using RNA from different time points of methyl jasmonate treated leaves of banana plantlets. In each case, expression level is expressed as relative fold change as compared to the control leaves (zero hour). (**b**) Phytochemical analysis of methanolic extracts from different time points of methyl jasmonate treated leaves of banana plantlets. Compounds were quantified by separating methanolic extracts using HPLC. The graph shows values ± SD of three samples from each of the independent plantlets.

The response of methyl jasmonate (MJ) over flavonoid biosynthesis in banana was studied through qPCR using RNA isolated from the leaves of *in vitro* grown plants exposed with MJ at different time points. Analysis suggests that expression of genes encoding enzymes associated with flavonoid biosynthesis is responsive to MJ. Although MJ led to the enhancement in the expression of most of the genes, the modulation was differential for different genes (Fig. 8a). In the case of *MaCHS* and *MaF3′5′H* expression, a significant increase in the expression was observed at 4 h which continued till 72 h. The expression pattern of *MaCHI, MaF3H* and *MaDFR* increased significantly at 2 h which continued till 72 h. The expression patterns of *MaANS* and *MaANR* increased significantly after 24 h to 72 h however, the expression of *MaFLS* and *MaLAR* did not increase significantly in response to MJ treatment (Fig. 8a). To assess the impact of MJ over flavonoid content, targeted metabolic profiling was carried out at different time points (Fig. 8b). MJ treatment led to the increase in quercetin, kaempferol, rutin and taxifolin content as compared to control samples. These results suggest that flavonoid biosynthetic genes are responsive to JM treatment in banana.

## Discussion

The flavonoid biosynthesis has been an important target for genetic manipulation in plants owing to their role in pigmentation in flowers, providing insect resistance as well as their health beneficial properties^1^,^45^,^46^,^47^,^48^,^49^. The biosynthesis of the diverse group of flavonoids is tightly regulated by various spatial and temporal cues that can limit the accumulation of these compounds in plants^50^. In banana, peel and pulp of the fruit have been reported to contain various secondary metabolites including polyphenols and flavonoids^51^. A complete insight about the genes and enzymes involved in flavonoid biosynthesis in this fruit crop would be essential in the strategically selecting gene(s) to enhance flavonoid biosynthesis in this fruit crop through biotechnological approach. In addition, most of the cultivated varieties of bananas are triploid in nature, hence sterile and provide the natural barrier to the cross pollination. Therefore, the application of transgenic approach for the improvement of this crop would be biologically safe and useful tool to introduce desirable traits. Considering important role of flavonoids in human health, in the present work, we have carried out a comprehensive survey of different gene families involved in the flavonoid biosynthetic pathway in important fruit crop, banana.

Our genome-wide analysis identified 28 genes belonging to 9 gene families associated with flavonoid biosynthesis in banana. Homology, exon-intron architecture and phylogenetic analyses suggested that identified genes are functional homologs of the genes associated with flavonoid biosynthesis from closely-related monocots. Various studies suggest that plant genes encoding enzymes of diverse functions might evolve from one of the two evolutionary processes. In the first case, single gene develops multifunctionality and produces various products utilizing different types of substrate^31^,^52^,^53^. In the next case, gene duplication and mutational evolution might lead to gene for the new function^54^. Similar to CHS superfamily in most of the plants, banana CHS contain single intron (Fig. S2). Apart from presence of single intron in CHS encoding genes in most of the plants, a few CHS encoding genes have been characterized as intron-less or with more than two introns^55^,^56^. In past, metabolic profiling of banana suggested presence of different derivatives of myricetin^38^,^39^. Interestingly, in banana F3′5′H family has seven members and might be responsible for the biosynthesis of myricetin and its derivatives. Different homologs of the gene may possibly encode different isoforms of the enzyme which might act in a tissue-specific manner.

To the best of our knowledge, there is no report available for the comprehensive analysis of various classes of flavonoids in different parts of the banana including different developmental stages of the fruit. We analyzed different tissues of banana such as young leaves, pseudostem, bract, root as well as peel and pulp of fruit at different developmental stages. Our analysis suggests that unripe peel of banana fruit contains the maximum amount of all the metabolites identified as compared to other tissues. Metabolites such as rutin, catechin and epicatechin are the predominant flavonoids in the banana peel. In our analysis, the highest amount of rutin (143.14 μg/g DW) was detected in the peel of the early developmental stage of the fruit. This is well correlated with expression analysis where flavonol biosynthetic genes were highly expressed at the early developmental stages of the fruit (Fig. 5). Previously, a large variation in the flavonol accumulation in the different varieties of banana has been reported^39^, which might be due to differences in the cultivars, sample processing and method of analysis as well as the selection of the developmental stages. In our analysis, flavonol accumulation and expression of associated genes were enhanced in the young fruit. Similar results were observed in apple fruit where flavonols were localized at the early stage of fruit development^30^. Similar to our study, naringenin, catechin and epicatechin has been previously reported in banana by other researchers^57^,^58^,^59^. The analysis suggested that PAs and transcripts associated with their biosynthesis (*MaANR* and *MaLAR*) were abundant at the early developmental stages of the fruit. However, after fruit maturation, there is a shift in flavonoid biosynthesis from PA to anthocyanin biosynthesis which acts as antimicrobials and might be involved in protection of the fruit from pathogens and fungi^36^.

The metabolite profiling, carried out in this study, also suggested that peel contain the higher amount of flavonoids than pulp of the fruit. The higher content of flavonoids in the peel may confer disease resistance and protection from herbivores and insects to the fruit. The higher content of flavonoids and the corresponding biosynthetic genes in the berry skin have already been correlated with insect resistance^60^. It has been well reported that lignins and tannins are involved in the strengthening of the cell wall and associated with the defence signaling^58^,^61^,^62^. Anthocyanidine enriched banana *cv* Mysore have been reported to provide resistance to black leaf streak disease whereas the other cultivar (Nanicao) lacking anthocyanidine is susceptible to black leaf streak disease^58^. Among flavonoids, flavan, 3-4-diols (leucoanthocyanidins) has also been reported in the banana pulp^63^. Similar to our result, previous reports also suggested that unripe banana contains the higher content of flavonoid (leucoanthocyanidins) as compared to the ripe tissue^63^. Our expression analysis of genes encoding DFR and ANS in different tissues and fruit development stages suggested that ANS and DFR may regulate anthocyanin biosynthesis pathway in banana.

Light is one of the most important modulators of flavonoid biosynthesis^64^,^65^,^66^. In the present study, dark exposure resulted in the decreased expression of genes involved in the flavonoid biosynthesis in banana plants. This clearly suggests that light-regulated factors may also be involved in transcriptional regulation of flavonoid biosynthesis in banana. In Arabidopsis, CHS has been extensively studied for its transcriptional activation through different qualities of light such as UVA/blue light, UVB and far-red light^67^. The promoter region of CHS and other biosynthetic genes are defined to have LRUs (Light Responsive Units) which binds to light responsive MYBs^65^.

Our study also suggests that MJ has an inducing effect over flavonoid biosynthesis. Similar results were also observed for anthocyanin biosynthesis in diverse plant species including Arabidopsis^68^,^69^. The Arabidopsis mutant coronatine insensitive1 (*coi1*), being defective in JA signalling is unable to induce anthocaynin biosynthesis in response to JA^69^. The Jasmonate Zim-domain (JAZ) proteins that are substrates of CORONATINE INSENSITIVE1 (COI1) based SCFCOI1 complex negatively regulate various plant JA responses including anthocyanin accumulation.

### Conclusions and future perspective

We have developed a comprehensive resource of gene families involved in flavonoid biosynthesis in banana. Transcript profiling and phytochemical analysis in various tissues, pulp and peel of different developmental stages of fruit as well as under different stresses suggest tight spatial and temporal regulation of flavonoid biosynthesis in banana. Future research involving development of transgenic banana lines with modified expression of specific genes should elucidate the function of individual gene family members. The manipulation of expression of the structural genes encoding the key enzymes of the limiting steps in the pathway appears to be an attractive approach to modify the flavonoid content in plants^70^. Apart from the biosynthetic genes, diverse families of regulatory proteins or transcription factors as well as miRNAs have been implicated in the regulation of flavonoid biosynthesis^71^. Therefore, there is a need to explore the transcription factors as well as miRNAs involved in the flavonoid biosynthesis to develop comprehensive understanding about flavonoid biosynthesis in banana.

## Methods

### Bioinformatics analysis

The whole genome sequence of banana (*Musa acuminata*) was downloaded from the Banana Genome Hub database (http://banana-genome.cirad.fr/)^16^. BLAST searches were performed using sequences of *Zea mays, Oryza sativa, Arabidopsis thaliana, Populus tremuloides* and *Vitis vinifera* as a query sequence to search similar protein sequence in *M. acuminata* database. Banana proteins, resulting from each BLAST search were pooled, and redundant sequences were removed. The protein sequences, thus retrieved were aligned with that of maize, rice, Arabidopsis, *Populus* and *Vitis*. Further refinement of gene models of banana proteins was carried out by retrieving corresponding genomic sequences and by using Fegenesh program (www.omictools.com/fgenesh-s1037.html). Finally, manual inspection of intron and exon boundaries was carried out. The analysis for the presence of introns and exons was carried out by using tool available at http://gsds.cbi.pku.edu.cn/.

### Alignment, phylogenetic analysis and chromosomal localization

Multiple sequence alignments were performed using Clustal X programme. The phylogenetic analysis was performed using Maximum likelihood method by MEGA 6 software with 1000 bootstrap value^72^ (http://www.megasoftware.net/). The chromosomal mapping of individual genes was carried out by performing BLASTx searches in a local database of Banana genome sequence.

### Plant material

Dessert banana, *Musa acuminata* (AAA genome), was taken as an experimental system in this study. Leaves, pseudostem, bract, fruit peel, and pulp, etc. were collected from the six month old banana plants growing in the field and stored at −80 °C, following freezing in liquid nitrogen. Fruits from three independent plants at different developmental stages were harvested to collect the samples. Fruits were collected at three weekly intervals, starting from 6 to 24 weeks and then treated with ethylene for ripening. Banana fingers from the same whorl of the hand representing similar developmental stage were treated with 100 μL/L ethylene for 24 h at 22 °C in dark and then allowed to ripen in the air as described previously^73^.

### Imposition of various stress treatments

*In vitro* grown plantlets were hardened in the growth chamber for 2 months. Young banana plants of uniform size were taken for different stress treatments. For dark exposure, plants were shifted in dark chamber with similar growth conditions as in the growth chamber as described previously^3^. The samples were collected at different time points of complete darkness condition. The similar plants grown in the growth chamber following normal photoperiod (16 light/ 8dark) were taken as experimental control. The 250 μM methyl jasmonate solution was used for evoking hormone stress as described in earlier study^74^. The sterile water treated plants were taken as experimental control. The young and healthy leaves of the same size from various stress-treated plants were collected at varying time points and used for RNA isolation and phytochemical analysis. In each case of stress treatment, samples were collected in triplicates.

### Total RNA isolation and cDNA synthesis

Total RNA was isolated from different banana tissues according to the previously described protocol^75^. For abiotic stress treated samples, total RNA was extracted from young leaves of banana plants using the Spectrum Plant Total RNA kit (Sigma-Aldrich, USA). Each RNA sample was treated with DNase I Digest kit (Sigma-Aldrich, USA) to eliminate DNA contamination. The integrity and size distribution of total RNA were analyzed by the agarose gel electrophoresis.

### Differential gene expression analysis

The quantitative real-time PCR (qPCR) expression was carried out with an ABI 7700 Sequence Detector (Applied Biosystems, USA). The transcripts were quantified by SYBR Green chemistry. The amount of cDNA was normalized by using amplification of housekeeping banana actin as an internal control. The data from real-time PCR amplification was estimated in terms of comparative fold expression following 2^−∆∆ct^ method. The list of different primers used in the study is given in the Supplementary Table S1.

### Extraction of plant material for quantitative estimation of flavonoids

Dried and powdered plant material (1 g) was extracted three times successively with 10 ml of methanol: water (80:20 v/v) using an IKA analytical mill (Staufen, Germany). Samples were homogenized and put in on an orbital shaker for 15 min at room temperature^76^,^77^. The Filtrate was collected in clear glass HPLC vial (Agilent Technologies, USA) and inject into HPLC-MS-MS.

### Liquid chromatography-Tandem mass spectroscopy and data analysis

All the samples were analyzed on an HPLC system (1260 infinity series, Agilent Technologies, Singapore) equipped with a pump, autosampler, column compartment and thermostat using Agilent Zorbax Eclipse Plus column (4.6 × 100 mm, 3.5 μm) maintained at 40 °C. A guard column of the same chemistry was also used. The mobile phase consisted aqueous solution of 0.1% formic acid (LC-MS grade; Solution A) and 0.1% formic acid in acetonitrile (LC-MS grade; Solution B). The gradient was programmed as follows: 0–2 min, solution B 2%; 2–22 min, Solution B 2-98%; 22.01–25 min, Solution B 98-2%. Other chromatographic parameters included a constant flow of 0.6 ml/min, injection volume was 5 μl, autosampler temperature was set at 10 °C and a run time of 25 min including equilibration. Before analysis, all of the samples were filtered through a 0.22 μm syringe membrane filters (Millipore, USA).

The detection was performed using a HPLC system coupled to a triple quadrupole system QTRAP 5500 (ABSciex, Singapore) using ESI probe both mode of ionisation. The voltage was set at 5.5 KV for positive ionisation and −4.5 KV for negative ionisation, The value of Gas1 and Gas2 was set at 50, curtain gas (CUR) was set at 30, collision assisted dissociation (CAD) was set at medium, temperature of the source (TEM) was set at 550 °C. The mass spectrometer was used in multiple reactions monitoring (MRM) mode. Identification and quantification analysis was performed using Analyst software (version 1.5.2) and MultiQuant software (version2.0.2).

### Statistical analysis

For all the experiments three biological and three technical replicates were used for the analysis. The data were analyzed by Student’s paired t-test, and the mean values under each treatment were compared at P ≤ 0.05–0.001.

## Additional Information

**How to cite this article**: Pandey, A. *et al*. Genome-wide Expression Analysis and Metabolite Profiling Elucidate Transcriptional Regulation of Flavonoid Biosynthesis and Modulation under Abiotic Stresses in Banana. *Sci. Rep.* **6**, 31361; doi: 10.1038/srep31361 (2016).

## Supplementary Material

Supplementary Information

Supplementary Information

## Figures and Tables

**Figure 1:**
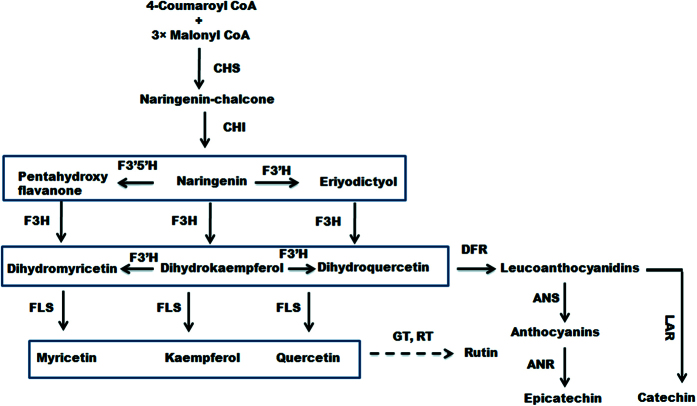
Schematic representation of flavonoid biosynthetic pathway. Dashed arrows represent multiple enzymatic steps. ANS, anthocyanidin synthase; ANR, anthocyanidin reductase; CHS, chalcone synthase; CHI, chalcone isomerase; DFR, dihydro flavanol 4-reductase; F3H, flavanone 3-hydroxylase; F3′H, flavonoid 3′-hydroxylase; F3′5′H, flavonoid 3′5′ hydroxylase; FLS, flavonol synthase; LAR, leucoanthocyanidin reductase.

**Table 1:** Structural features of flavonoid biosynthetic genes in banana.

Musa acuminata reference genome sequence locus id	Name	Start	End	Number of amino acid	MW (kDa)	PI	TMH	Signal peptide	Duplication event
GSMUA_Achr6P08170_001	MaCHS1	5448636	5450145	369	40.96	7.15	—	—	Dispersed
GSMUA_Achr6P10910_001	MaCHS2	7269753	7271280	329	35.81	8.43	—	—	Dispersed
GSMUA_Achr6P16370_001	MaCHS3	10908536	10910004	314	34.41	8.42	—	—	Dispersed
GSMUA_Achr6P08180_001	MaCHS4	5459774	5461332	401	44.46	9.22	Yes	—	Dispersed
GSMUA_Achr10P12200_001	MaCHS5	21295737	21297266	325	35.41	6.67	—	—	Dispersed
GSMUA_Achr10P12260_001	MaCHS6	21315173	21318120	344	37.50	6.68	—	—	Dispersed
GSMUA_Achr4P16830_001	MaCHI1	15371260	15372308	237	25.00	5.01	—	—	Dispersed
GSMUA_Achr11P23630_001	MaCHI2	23473258	23474634	288	32.47	5.96	—	—	Dispersed
GSMUA_Achr2P02770_001	MaF3H1	8468601	8469887	373	41.43	5.3	—	—	Proximal
GSMUA_Achr7P16780_001	MaF3H2	17165381	17166656	374	41.38	5.14	—	—	Tandem
GSMUA_Achr3P07030_001	MaFLS1	4742251	4744098	334	37.42	5.61	—	—	Tandem
GSMUA_Achr8P23330_001	MaFLS2	27816227	27817456	333	37.06	5.72	—	—	Dispersed
GSMUA_Achr10P25240_001	MaFLS3	29326369	29328343	336	37.79	6.23	—	—	Tandem
GSMUA_Achr10P25250_001	MaFLS4	29328762	29330222	314	35.18	5.74	—	Yes	WGD
GSMUA_Achr3P30350_001	MaF3′H	29141939	29143583	458	50.24	6.55	Yes	Yes	Dispersed
GSMUA_Achr2P00210_001	MaF3′5′H1	730250	732197	452	49.66	6.51	Yes	Yes	Dispersed
GSMUA_Achr8P05810_001	MaF3′5′H2	3777871	3779637	476	52.82	8.1	—	Yes	Dispersed
GSMUA_Achr8P08630_001	MaF3′5′H3	5801769	5803358	445	49.69	9.13	Yes	—	Dispersed
GSMUA_Achr9P17300_001	MaF3′5′H4	11832622	11834707	474	52.60	5.8	Yes	—	Dispersed
GSMUA_Achr11P05200_001	MaF3′5′H5	3883787	3885381	424	46.70	8.51	—	Yes	Dispersed
GSMUA_Achr11P15630_001	MaF3′5′H6	17315963	17317643	453	50.26	9.26	Yes	—	Dispersed
GSMUA_AchrUn_randomP12120_001	MaF3′5′H7	58016718	58018562	421	46.45	8.95	—	Yes	Dispersed
GSMUA_Achr3P31110_001	MaDFR1	29563009	29564492	357	40.21	5.12	—	—	WGD
GSMUA_Achr4P10640_001	MaDFR2	7720951	7722685	353	39.73	5.86	—	—	WGD
GSMUA_Achr4P24540_001	MaDFR3	24297768	24299513	353	39.36	5.79	—	—	WGD
GSMUA_Achr5P04080_001	MaANS	2913298	2914465	361	40.68	5.61	—	—	WGD
GSMUA_Achr8P01490_001	MaANR	1217455	1218907	354	38.28	6.27	—	—	WGD
GSMUA_AchrUn_randomP18370_001	MaLAR	87332970	87335842	352	37.88	6.32	—	—	WGD
