# Supplementary material for: Genome-wide Expression Analysis and Metabolite Profiling Elucidate Transcriptional Regulation of Flavonoid Biosynthesis and Modulation under Abiotic Stresses in Banana
Source: Sci Rep. 2016 Aug 19;6:31361. doi: 10.1038/srep31361 (PMC4990921; doi:10.1038/srep31361)
Supplement: Supplementary Information [file srep31361-s1.pdf]

Supplementary File S1

Genome-wide expression analysis and Metabolite Profiling Elucidate Transcriptional Regulation of Flavonoid Biosynthesis and Modulation under Abiotic Stresses in Banana

Ashutosh Pandey[1,2,*], Anshu Alok[2], Deepika Lakhwani[1], Jagdeep Singh[2], Mehar H. Asif[1] & Prabodh K. Trivedi[1,*]

[1]CSIR-National Botanical Research Institute, Council of Scientific and Industrial Research (CSIR-NBRI), Rana Pratap Marg, Lucknow-226001, INDIA

[2]National Agri-Food Biotechnology Institute (NABI), (Department of Biotechnology, Government of India), C-127, Industrial Area, Phase VIII, S.A.S. Nagar, Mohali 160071, India

[*]Authors for correspondence:

PKT: prabodht@nbri.res.in; prabodht@hotmail.com

AP: ashutosh_biotech@yahoo.co.in

>GSMUA_Achr6T08170_001_(MaCHS1)_Genomic

GTCATCTACTACAAATGTCCAAACTCCACGACATCCAAAGCAAGCCATCAACTGTTCGAGCTGCAATACTCGGCT
TGTGGCAGTCGTTGGTGAAGATTAGAAGAGCCAGGAGTCATGGCCAAGGTGGAGGAGATCCGGCGATCGCAGAGG
GCTGAGGGGCCAGCGACGGTGCTCGCCATCGGCACCTCCAACCCGGACAACGTCGTGTACCAGGCTGACTACCCC
GACTATTACTTCCGCATCACGAAGAGCAACCACCTCCCAGAGCTCAAACAGAAGTTTAAGAGAATTTGTGAGTGA
CTGATACATATTCTCTACCATTTCTTTGGCATTTCCTTTGTTTCCTTTATACCAGATTTTTTTTTTATTTTTCT
TCTGCGTAAATGACCACAAAGGTAGGATTTGTTTCCCCCTTCCTAAGCCTTGTTACAGGGAAGGATGAGAAAAGG
CAGCAGTTTGGACTAAGTTGTTCCTCTGCAACAGTGATCATCAGTGTACTATCTCCTATATATCTATCTAAAAGA
ATAAGAAAGCATGACATTATTACAAGTGAAAGACAGAGGTCTATTGAGAAATATAAGACTCTTAATTGCCATGCT
TGTTGCACTCCCTGATGGTTCAGGTGAGAAGACGATGATACGTAAACGTTACATGTTCCTCAACGAGGAGATCCT
GAAAGCGAACCCTAACATCGCCGAATACATGGCACCGTCAATGGACGTCCGGCGGGACATCTTGGCCGTGGAGGT
GCCGAAACTGGCCAAGGAGGCGGCAGTCAAGGCCATCGAAGAGTGGGGGCACCCCAAGTCCAGGATCACCCACCT
GGTCTTCTGCACCACCGCCAGCTTCGACTCGCCCGGCCACGACTACCAGCTTGTCAAGCTCCTCGGACTCAGCCC
TTCCATTAACCGGTTCACGTTGTCCCACCATGGCTGCTTCGCCGGCGGCACGGTGCTCCGCCTCGCCAAGGATCT
CGCGGAGAACAACCGCGGCGCCCGGGTGCTCGTGGTCTGTTCCGAGCTCACCGCCGTCACCTTCCGCGGGGCCAC
GGAGACCCACCTCGACAACCTCGTCGGACAGGCCCTGTTCGGAGACGGCGCCGCCGCCGTCATCATCGGAGCTGA
CCCCGACGGAGCCACGGAGCGTCCCCTCTTCCAACTCGTCTCGGCCAGCCAGACCCTCCTCCCCGACTCCGACGG
CGCCATCGAGGGCCACCTCAAGGAGGTCGGCCTCACCTTCCATCTGCTCAAGGACGTGCCCAGGATCATTGCCAA
GAACATCGAGCAGAATCTCGTGGAGGCCTTCGAGTCGTTGGGGATCAGCGACTGGAACTCCATCTTCTGGATCGT
GCACCCCGGTGGATCCGCGATCCTCGACGCGATGGAGGCCAAGCTGGGGCTGGAGAAGGAAAAGCTGAAGGCGAC
GAGGCAGGTGATGACCGAATACGGGAACATGTACAGCGCCTGCGTGCTGTTCATCCTCGACGAGATGAGGAAGCG
GTCGGCCGAGGACGGGAAGGCGACCACCGGCGACGGGTTGGAGTGGGGGGTGCTCTACGGGTTCGGTCCTGGCCT
CACCGTGGAGACCGTGGTCTTGCGCAGCGTCGCCATATCTTCCCGTTGAAAGCAGAATGACCATCGTTTGCTATT
GGCCTCAATTTGACATTGTCAACCGGATCGACTTCTCTAGCAGTGATGATACTGCTTTGCATCTACCTACTACTT
AGTAAGATATATGCG

>GSMUA_Achr6T08170_001_(MaCHS1)_Modified CDS

ATGGCCAAGGTGGAGGAGATCCGGCGATCGCAGAGGGCTGAGGGGCCAGCGACGGTGCTCGCCATCGGCACCTCC
AACCCGGACAACGTCGTGTACCAGGCTGACTACCCCGACTATTACTTCCGCATCACGAAGAGCAACCACCTCCCA
GAGCTCAAACAGAAGTTTAAGAGAATTTGTGAGAAGACGATGATACGTAAACGTTACATGTTCCTCAACGAGGAG
ATCCTGAAAGCGAACCCTAACATCGCCGAATACATGGCACCGTCAATGGACGTCCGGCGGGACATCTTGGCCGTG
GAGGTGCCGAAACTGGCCAAGGAGGCGGCAGTCAAGGCCATCGAAGAGTGGGGGCACCCCAAGTCCAGGATCACC
CACCTGGTCTTCTGCACCACCGCCAGCTTCGACTCGCCCGGCCACGACTACCAGCTTGTCAAGCTCCTCGGACTC
AGCCCTTCCATTAACCGGTTCACGTTGTCCCACCATGGCTGCTTCGCCGGCGGCACGGTGCTCCGCCTCGCCAAG
GATCTCGCGGAGAACAACCGCGGCGCCCGGGTGCTCGTGGTCTGTTCCGAGCTCACCGCCGTCACCTTCCGCGGG
GCCACGGAGACCCACCTCGACAACCTCGTCGGACAGGCCCTGTTCGGAGACGGCGCCGCCGCCGTCATCATCGGA
GCTGACCCCGACGGAGCCACGGAGCGTCCCCTCTTCCAACTCGTCTCGGCCAGCCAGACCCTCCTCCCCGACTCC
GACGGCGCCATCGAGGGCCACCTCAAGGAGGTCGGCCTCACCTTCCATCTGCTCAAGGACGTGCCCAGGATCATT
GCCAAGAACATCGAGCAGAATCTCGTGGAGGCCTTCGAGTCGTTGGGGATCAGCGACTGGAACTCCATCTTCTGG
ATCGTGCACCCCGGTGGATCCGCGATCCTCGACGCGATGGAGGCCAAGCTGGGGCTGGAGAAGGAAAAGCTGAAG
GCGACGAGGCAGGTGATGACCGAATACGGGAACATGTACAGCGCCTGCGTGCTGTTCATCCTCGACGAGATGAGG
AAGCGGTCGGCCGAGGACGGGAAGGCGACCACCGGCGACGGGTTGGAGTGGGGGGTGCTCTACGGGTTCGGTCCT
GGCCTCACCGTGGAGACCGTGGTCTTGCGCAGCGTCGCCATATCTTCCCGTTGA

>GSMUA_Achr6P08170_001_(MaCHS1)_Protein

MAKVEEIRRSQRAEGPATVLAIGTSNPDNVVYQADYPDYYFRITKSNHLPELKQKFKRICEKTMIRKRYMFLNEE
ILKANPNIAEYMAPSMDVRRDILAVEVPKLAKEAAVKAIEEWGHPKSRITHLVFCTTASFDSPGHDYQLVKLLGL
SPSINRFTLSHHGCFAGGTVLRLAKDLAENNRGARVLVVCSELTAVTFRGATETHLDNLVGQALFGDGAAAVIIG
ADPDGATERPLFQLVSASQTLLPDSDGAIEGHLKEVGLTFHLLKDVPRIIAKNIEQNLVEAFESLGISDWNSIFW

IVHPGGSAILDAMEAKLGLEKEKLKATRQVMTEYGNMYSACVLFILDEMRKRSAEDGKATTGDGLEWGVLYGFGP
GLTVETVVLRSVAISSR

>GSMUA_Achr6T08180_001_(MaCHS2)_Genomic

AACAAGGAAGATGCCGACGCACTCACCAAACCCATCGTGGCTTCACGTATAGCCCGTCCCTCCCACCTATCCTCT
ACTATAAATCTTCAAGCTCCATTCCATCCATCTCCACCACATCCAAAGCAAGCTGAAGTGCTCGGCTTGTGGTAG
TTGTTTGTGAGGATAGGAAGAGAGAGAAGCAGGAGTCATGGCCAAAGTCGAGGAGATCCGGCGATCGCAGAGGGC
AGAGGGGCCAGCGACGGTACTTGCCATCGGCACCGCCAACCCGGCCAACGTCGTGTACCAGGCCGACTACCCTGA
CTACTACTTCCGCATCACCAGGAGCAACCACCTCCCTGAGCTCAAACAGAAGTTTAAGAGAATGTGTGAGTGACT
TATACGTACTCTCGACCGTTTCTTTGGTAGTTGCTTTGTTGCATGGCGTTATTACTAGTGACAGACAGAGATCTA
CTAACTGTACTCGTCGCTAGATAAGAGTCTTGGTTGTGATGCTCGTACTTCGTGATGGTTCAGGTGACAAGACAA
TGATCCGTAAACGTTACATGTTCCTCAACGAGGAGATCCTGAAAGCGTACCCTAACATCGGCGCGTACATGGCAC
CGTCACTGGACGTCCGGCGGGACATCATGGCCGTGGAGGTGCCGAAGCTGGCCATGCAGGCGGCAGTAAAGGCCA
TCGAAGAGTGGGGGCACCCCAAGTCCAGGATCACCCACCTGGTCTTCTGCACCACCGTCAGTTTCGACTTGCCCG
GCCACGACTACCAGCTTGTCAAGCTCCTCGGACTCAACCTTTCCATCAACCGCTTCACGTTGTCCCAGCATGGCT
GCTTCGCCGGCGGCACGGTGCTCCGCCTCGCCAAGGACATAGCAGAGAACAACCGCGGCGCCCGGGTGCTCGTGG
TCTGCTCCGAGCTCACCACAGTCACCTTCCGCGGGGCGGCGGAAACTCACCTCGACAACCTCGTCGGACAGGCCC
TGTTCGGCGACGGCGCCGCCGCCGTCATCATCGGAGCTGACCCCGACCCGGCCACGGAGCGACCCCTCTTTCAGC
TCGTCTCGGCCAGCCAGACCCTCCTCCCCGACTCCGACGGCGCCATCGAGGGCCACCTCAAGGAGGTCGGCCTCA
CCTTCCATCTGCTCAGGGACGTGCCCAGGATCATTGCCAAGAACATCGAGCAGAATCTCGTGGAGGCCTTCGAGC
CGCTGGGGATCAGCGACTGGAACTCCATCTTCTGGATCGCGCACCCCGGCGGACCCGCGATCCTCGACGCGATGG
AGGCCAAGCTGGGGCTGGGGAAGGCAAAGCTGAAGGCGACGAGGCAGGTGATGACCGAGTACGGGAACATGTCCA
GCGCCTGCGTGCTGTTCATCCTCGACGAGATGAGGAAGCGGTCGGCCGAGGACGGGAAGGCGACCACCGGCGAGG
GGTTGGAGTGGGGGGTGCTCTACGGGTTCGGTCCCGGCCTCACAGTGGAGACCGTGGTCTTGCGCAGCGTCGCCA
TTTCTTCCCGTTGATAGCAGAATGGCCATCCTTTGATACAAGCCTCACTTCGCCACTGTCAACTGGATCGACTTG
TTTAGTTGTGATTCTATTATAGTATGTAGTAAGATATATGCGAGGCAAAACTTGTGATGTATTAAGGAAGACGTG
CATGCACGTCTTAAGAAAGTGCCGAAGAATAAGACGGTGGCTCGTATGTTTGTGGTAATTAATATATTTCATCTT
GTCGCTCTACTTTTGCCTTAAAACATTTCTCTTGTCATCTTGTGTGCAATGCAGCCAAGTATCAAATCTGGAATA
TAAGACTGCAAGGTTGTCTTTGGTTGGGCATAATACATCAAATGTTCATATGATGATCAAAGCCCAATAAGAGAT
AAAATAATTCATCAA

>GSMUA_Achr6T08180_001_(MaCHS2)_ Modified CDS

ATGGCCAAAGTCGAGGAGATCCGGCGATCGCAGAGGGCAGAGGGGCCAGCGACGGTACTTGCCATCGGCACCGCC
AACCCGGCCAACGTCGTGTACCAGGCCGACTACCCTGACTACTACTTCCGCATCACCAGGAGCAACCACCTCCCT
GAGCTCAAACAGAAGTTTAAGAGAATGTGTGACAAGACAATGATCCGTAAACGTTACATGTTCCTCAACGAGGAG
ATCCTGAAAGCGTACCCTAACATCGGCGCGTACATGGCACCGTCACTGGACGTCCGGCGGGACATCATGGCCGTG
GAGGTGCCGAAGCTGGCCATGCAGGCGGCAGTAAAGGCCATCGAAGAGTGGGGGCACCCCAAGTCCAGGATCACC
CACCTGGTCTTCTGCACCACCGTCAGTTTCGACTTGCCCGGCCACGACTACCAGCTTGTCAAGCTCCTCGGACTC
AACCTTTCCATCAACCGCTTCACGTTGTCCCAGCATGGCTGCTTCGCCGGCGGCACGGTGCTCCGCCTCGCCAAG
GACATAGCAGAGAACAACCGCGGCGCCCGGGTGCTCGTGGTCTGCTCCGAGCTCACCACAGTCACCTTCCGCGGG
GCGGCGGAAACTCACCTCGACAACCTCGTCGGACAGGCCCTGTTCGGCGACGGCGCCGCCGCCGTCATCATCGGA
GCTGACCCCGACCCGGCCACGGAGCGACCCCTCTTTCAGCTCGTCTCGGCCAGCCAGACCCTCCTCCCCGACTCC
GACGGCGCCATCGAGGGCCACCTCAAGGAGGTCGGCCTCACCTTCCATCTGCTCAGGGACGTGCCCAGGATCATT
GCCAAGAACATCGAGCAGAATCTCGTGGAGGCCTTCGAGCCGCTGGGGATCAGCGACTGGAACTCCATCTTCTGG
ATCGCGCACCCCGGCGGACCCGCGATCCTCGACGCGATGGAGGCCAAGCTGGGGCTGGGGAAGGCAAAGCTGAAG
GCGACGAGGCAGGTGATGACCGAGTACGGGAACATGTCCAGCGCCTGCGTGCTGTTCATCCTCGACGAGATGAGG
AAGCGGTCGGCCGAGGACGGGAAGGCGACCACCGGCGAGGGGTTGGAGTGGGGGGTGCTCTACGGGTTCGGTCCC
GGCCTCACAGTGGAGACCGTGGTCTTGCGCAGCGTCGCCATTTCTTCCCGTTGA

>GSMUA_Achr6P08180_001_(MaCHS2)_Protein

MAKVEEIRRSQRAEGPATVLAIGTANPANVVYQADYPDYYFRITRSNHLPELKQKFKRMCDKTMIRKRYMFLNEE
ILKAYPNIGAYMAPSLDVRRDIMAVEVPKLAMQAAVKAIEEWGHPKSRITHLVFCTTVSFDLPGHDYQLVKLLGL
NLSINRFTLSQHGCFAGGTVLRLAKDIAENNRGARVLVVCSELTTVTFRGAAETHLDNLVGQALFGDGAAAVIIG
ADPDPATERPLFQLVSASQTLLPDSDGAIEGHLKEVGLTFHLLRDVPRIIAKNIEQNLVEAFEPLGISDWNSIFW

IAHPGGPAILDAMEAKLGLGKAKLKATRQVMTEYGNMSSACVLFILDEMRKRSAEDGKATTGEGLEWGVLYGFGP
GLTVETVVLRSVAISSR

>GSMUA_Achr6T10910_001_(MaCHS3)_Genomic

TCCCTATAAATCTTCCCGGTGCCTCCCCATAGCTTCTCCATCCTGGAAGCGGCTCCGCGTCGCCTTGGCACTACT
CCTACGACCGCTCCCCGGCCATCCCACCGTCCGTAACGATGGCCGACCTCCAGGAGATCCGCCGCTCGCAGAGGG
CCGGGGGACCCGCCGCGGTGCTCGCCATCGGCACCGCCACGCCGGCCAACGTCCTCTACCAAGCTGACTACCCCG
ACTACTACTTCCGCATCACCAAGAGCGACCACCTGACCGAGCTCAAGGAAAAGTTCAAGAGGATGTGTACGTCAC
CTCTACCTCTTCTTGTGATATTACACTCACCCTATTTTGTTCCTTGATCTTTGAGAGACTTCGTGACCGACAAGT
GTGTAGTGTGTGCCTCTCCAGACAGTCTGAGAGTCGTCCAAGAAGCGTGACTTCTTTCTTGTTGATTTCATACGT
ACAATCCTGCTTATCACAAACCAGCTGATGGTGTTGTATTGTTACCATAATTTAGTAGTATTTTATTGCCTGGTA
GCATTTAGATAAGAGCTGTGTCATTGGTCTAGAATAGGACTTGTGCTTTCTGCTGGAAATGTTCTCTCCTTTACC
AAAGGACCGTGCGTTATTGCATGGTTTTCTGTGGCTTCAGGCGACAAGTCGATGATCCGAAAGCGTTACATGCAC
CTCAACGAGGAGATCCTCAAGGAGAACCCCAACATCTGCGCCTACATGGCGCCGTCGCTGGACGCGCGGCAGGAC
ATAGTGGTGGTGGAGATTCCGAAGCTGGGGAAGGAGGCTGCCGTCAAGGCCATCAAGGAGTGGGGCCAGCCCAAG
TCCAAGATCACCCACCTGGTCTTCTGCACCACCAGCGGTGTCGACATGCCCGGCGCCGACTACCAGCTCACCAGG
CTCCTCGGCCTCCGCCCCTCCGTCAACCGCCTCATGATGTACCAGCAGGGCTGCTTCGCGGGCGGCACCGTGCTC
CGCCTCGCCAAGGACCTGGCCGAGAACAACCGCGGCGCGCGCGTCCTCGTGGTTTGCTCCGAGATCACCGCCGTC
ACCTTCCGCGGGCCCTCGGAGTCCCACCTCGACAGCCTCGTCGGCCAGGCCTTGTTCGGGGACGGCGCTGCTGCC
ATCATCGTGGGGGCCGACCCGGACCCCGCGATCGAGCGGCCCCTGTTCCAGATCGTCTCCGCCAGCCAGACCATC
GTCCCGGACTCCGAGGGCGCCATCGACGGCCACCTGAGGGAGGTCGGCCTCACGTTCCACCTTCTCAAGGACGTG
CCGGGGCTGATATCCAAGAACATAGAGAAGAGCCTGGTGGAGGCGTTCAAGCCGCTAGGGATCGACGACTGGAAC
TCCATCTTCTGGATCGCGCACCCCGGCGGTCCGGCGATCCTCGACCAGGTGGAGGCCAAGATCGGACTGCAGAAG
GAGAAGATGCAGGCGACGAGGCGCGTGCTCAGCGAGTACGGCAACATGTCGAGCGCTTGCGTGCTGTTCATCCTG
GACGAGATGAGGAACCGGTCGGCGGCGGACGGCAAGGCGACCACCGGCGAGGGATTGAAGTGGGGAGTACTCTTC
GGCTTCGGGCCCGGGCTGACCGTGGAAACCGTCGTCTTGCACAGTATGCCAATCGTTGCAAACTGATGGCATGCA
GCACTAGCAAGAACTGAACTCGTCCATCTTTCTCTTCCAGTATTCTACGCCTAAGAAGCTGTTTAAGCGTGTCCG
ATGGTGTTCAATAAGATGTATTCTGTCAGCTCAGTTTGATATGGATTTGGCTTAGATCATCAGTTCTCATTACTT
TTGTTGATCAAAATGGAATTCACCATCCCTGCATATAAAACCGAGGCTGAGTAGGCTTAACCTCCATCAAGAAAT
ATCATCTTTTTCTTC

>GSMUA_Achr6T10910_001_(MaCHS3)_Modified CDS

ATGGCCGACCTCCAGGAGATCCGCCGCTCGCAGAGGGCCGGGGGACCCGCCGCGGTGCTCGCCATCGGCACCGCC
ACGCCGGCCAACGTCCTCTACCAAGCTGACTACCCCGACTACTACTTCCGCATCACCAAGAGCGACCACCTGACC
GAGCTCAAGGAAAAGTTCAAGAGGATGTGCGACAAGTCGATGATCCGAAAGCGTTACATGCACCTCAACGAGGAG
ATCCTCAAGGAGAACCCCAACATCTGCGCCTACATGGCGCCGTCGCTGGACGCGCGGCAGGACATAGTGGTGGTG
GAGATTCCGAAGCTGGGGAAGGAGGCTGCCGTCAAGGCCATCAAGGAGTGGGGCCAGCCCAAGTCCAAGATCACC
CACCTGGTCTTCTGCACCACCAGCGGTGTCGACATGCCCGGCGCCGACTACCAGCTCACCAGGCTCCTCGGCCTC
CGCCCCTCCGTCAACCGCCTCATGATGTACCAGCAGGGCTGCTTCGCGGGCGGCACCGTGCTCCGCCTCGCCAAG
GACCTGGCCGAGAACAACCGCGGCGCGCGCGTCCTCGTGGTTTGCTCCGAGATCACCGCCGTCACCTTCCGCGGG
CCCTCGGAGTCCCACCTCGACAGCCTCGTCGGCCAGGCCTTGTTCGGGGACGGCGCTGCTGCCATCATCGTGGGG
GCCGACCCGGACCCCGCGATCGAGCGGCCCCTGTTCCAGATCGTCTCCGCCAGCCAGACCATCGTCCCGGACTCC
GAGGGCGCCATCGACGGCCACCTGAGGGAGGTCGGCCTCACGTTCCACCTTCTCAAGGACGTGCCGGGGCTGATA
TCCAAGAACATAGAGAAGAGCCTGGTGGAGGCGTTCAAGCCGCTAGGGATCGACGACTGGAACTCCATCTTCTGG
ATCGCGCACCCCGGCGGTCCGGCGATCCTCGACCAGGTGGAGGCCAAGATCGGACTGCAGAAGGAGAAGATGCAG
GCGACGAGGCGCGTGCTCAGCGAGTACGGCAACATGTCGAGCGCTTGCGTGCTGTTCATCCTGGACGAGATGAGG
AACCGGTCGGCGGCGGACGGCAAGGCGACCACCGGCGAGGGATTGAAGTGGGGAGTACTCTTCGGCTTCGGGCCC
GGGCTGACCGTGGAAACCGTCGTCTTGCACAGTATGCCAATCGTTGCAAACTGA

>GSMUA_Achr6P10910_001_ (MaCHS3)_Protein

MADLQEIRRSQRAGGPAAVLAIGTATPANVLYQADYPDYYFRITKSDHLTELKEKFKRMCDKSMIRKRYMHLNEE
ILKENPNICAYMAPSLDARQDIVVVEIPKLGKEAAVKAIKEWGQPKSKITHLVFCTTSGVDMPGADYQLTRLLGL
RPSVNRLMMYQQGCFAGGTVLRLAKDLAENNRGARVLVVCSEITAVTFRGPSESHLDSLVGQALFGDGAAAIIVG
ADPDPAIERPLFQIVSASQTIVPDSEGAIDGHLREVGLTFHLLKDVPGLISKNIEKSLVEAFKPLGIDDWNSIFW
IAHPGGPAILDQVEAKIGLQKEKMQATRRVLSEYGNMSSACVLFILDEMRNRSAADGKATTGEGLKWGVLFGFGP
GLTVETVVLHSMPIVAN

>GSMUA_Achr6T16370_001_(MaCHS4)_Genomic

CTGCGGTCGGAGACAGCGACGGCTCTTCAGATGGCCTGCCGCCACACCATCATCGCTGCCCCCGAACGGAACGCC
TCCGCACGTGCGACTGGCAGGCCATGAAGCGAACGAAAAGATACTCTGCCTGTTAAAAAGGATATTTTTATGATT
CTTTTAATATTTTTGATAATATATTATCATAATTTTAATTTATATACATTTTAAGTATTATGAAAATGATTTAGA
TATTTTTTATACAGTTATTGTTAATTATATAATTACGTGTATGAATTCATTGTTACTTTCAATAGAAATTGAAGC
TAGTTGTTTCTTTCACGGGTTACAGGACTTCAGCCGGTGGAAGGAAGGGTGACGGTGGCGTGGCGTGCAATGTGC
CACGTCTCCTACCAAACTTTCCCTGCTCCTCGTGGCTTCTCATCTGCCCGTCCCCATTCGACGTGCCTCGACTAT
AAATCTCCCGGCGCCGTCCGTCGCCGCACCGTATCATCTGCAGGACAAACCAGCGCTATACCTTGAGCTACTACT
CGCTACTGCTGCTGCAGCTCGTTTGTGGCTGTCGGTGAGGGTAGCGAGCGCAGGAACATCCATGGCCAAGGTGCA
GGAGATCAGGAGTTCGCAGAGGGCCGAGGGGCCTGCCACGGTGCTCGCCATCGGCACCGCCACCCCTGCCAACGT
CGTGTACCAGGCCGAATACCCGGACTACTACTTCCGCATCACCAAGAGCGAGCACCTCACTGAGCTCAAGGAGAA
GTTTAAGAGGATGTGTGGGTGCTCCTATTTCTCTTTGTACTATTTATCTCTTTACTATCTAAAGTACCATTGCTT
GATTAAAGCTGCTTTTGTCATTTCTATGAACATAGAAGATGACAAAAGCCTTAAGAAAAAGAAAACTACTATTTC
CTGAGAACAAAAGACATGCATCTAGAGACTATTGTTCTTCTCTGAAACTAGCTAGGAAGGACGGAGGTAAAGGCG
TGTGGTTGTGATCTTAGTAACGCACGCATGCTGATGCAAGTGACCTGGTTTCGTGTCTTTTGACTGCATGGTTGT
TCAGGTGACAAGTCTATGATCCGCAAACGTTACATGCACCTCAACGAGGAGATCCTGCAAGAGAACCCTAACATG
TGCGCGTACATGGCGCCGTCGCTGGACGCCCGGCAGGACATCGTGGTGGTGGAGGTGCCGAAGCTCGGGAAGGAG
GCGGCAGCCAAGGCCATCAAGGAGTGGGGGCAGCCCAAGTCCAAGATCACCCACCTGGTCTTCTGCACCACCAGC
GGGGTCGACATGCCCGGGGCCGACTACCAGCTCACCAAGCTCCTCGGCCTCCGCCCATCCGTCAACCGGTTCATG
ATGTACCAGCAGGGCTGTTTCGCGGGCGGCACGGTGCTCCGCCTGGCCAAGGACCTGGCGGAGAACAGCCGCGGC
GCCCGGGTGCTCGTGGTCTGCTCCGAGATCACTGCTGTCACCTTCCGCGGGCCCTCGGAGTCTCACCTCGACAGC
CTCGTGGGGCAGGCTCTGTTCGGCGACGGCGCCGCCGCCATCATCGTCGGCGCAGACCCGGACCCCGTCACGGAG
CGCCCTATCTTCCAGCTCGTCTCCGCCAGCCAGACCATCCTCCCCGACTCCGAAGGCGCCATCGACGGCCACCTG
CTGGAGGTCGGCCTCACCTTCCACCTGCTCAAGGACGTGCCCGGCCTCATCTCCAAGAATATCGAGAGGAGCCTA
GTGGAGGCGTTCAAGCCGCTGGGGATCAGCGACTGGAACTCCATGTTCTGGATCGTGCACCCCGGCGGACCAGCG
ATCCTCGACCAGGTGGAGGCCAAGCTGGGCCTTGAGAAGGAGAAGATGAAGGCGACGCGGCAGGTGCTGAGCGAG
TACGGGAACATGTCCAGCGCATGCGTGCTCTTCATCCTCGACGAGATGAGGAAGCGATCCGCCGAGGACGGAAAG
GCGACCACCGGGGAGGGGCTGGAGTGGGGGGTGCTCTTCGGGTTCGGCCCGGGGCTGACGGTGGAGACGGTAGTC
TTGCACAGCATCCCCATCGCTGCCCGATGAAAGGACGAGAGAGCCTGCCATGCATCCAAGCTAGCTTTTCCCGCT
GCTGTCACGGGAATCTGTCCTTCATGCTATGATGCAATTGTATCTATCTATATTCACATAAATAAATGCACGCGT
CTGTGCATGTTTCGTGGCGCTTACACATCTCTTCTCGTTATACTTTGTTGTCCGACCATTCCAAGCCGACGCATT
GCACGGCAACGGTCGACATGGGTTGAACCCATCTTCTAGTATTGAACCACGTGGCAGCTTACATAAGTACCTAAA
TGGGCTAAGCAAATACTGCCATGAGACGTAATCAATTTACTGCATGTCACTCGCAGTCGAGTCAGACGATACGGT
GACAACAACGTGACGACTGGACCTCTCGGTCGGCACGTGCTTCGATTGACGTGTTAGTTGGAGCTGCGAGCTTAG
TGTGGTATACCTACCTATCAAAACTTTCATTATAATCATAATTTATAAGCATAGATTATAATTAATGTGATAATT
GATGTTTATAGATCAAAAATCTATATCAATATGCCTAATATGATATTTCTATTGAGACATTCGGACTGAACGTCA
TATACACCCTCGAATATAATGAAAATAATAATAATATAATGGTATTATTATATATGCTTATTGTTCGACCTATTG
AATTAAAATTTAATAACTAGTGTTATATTAAATATTCTTAGTAACCTAAATGAGATATTTTACAGTATGCTTTAG
TAATTATCGAGAATAAGTAAAAATTAATTCATGATAATGATTATGTGGGGAGAAAAGGTGGCCAG

>GSMUA_Achr6T16370_001_(MaCHS4)_ Modified CDS

ATGGCCAAGGTGCAGGAGATCAGGAGTTCGCAGAGGGCCGAGGGGCCTGCCACGGTGCTCGCCATCGGCACCGCC
ACCCCTGCCAACGTCGTGTACCAGGCCGAATACCCGGACTACTACTTCCGCATCACCAAGAGCGAGCACCTCACT
GAGCTCAAGGAGAAGTTTAAGAGGATGTGTGACAAGTCTATGATCCGCAAACGTTACATGCACCTCAACGAGGAG
ATCCTGCAAGAGAACCCTAACATGTGCGCGTACATGGCGCCGTCGCTGGACGCCCGGCAGGACATCGTGGTGGTG
GAGGTGCCGAAGCTCGGGAAGGAGGCGGCAGCCAAGGCCATCAAGGAGTGGGGGCAGCCCAAGTCCAAGATCACC
CACCTGGTCTTCTGCACCACCAGCGGGGTCGACATGCCCGGGGCCGACTACCAGCTCACCAAGCTCCTCGGCCTC
CGCCCATCCGTCAACCGGTTCATGATGTACCAGCAGGGCTGTTTCGCGGGCGGCACGGTGCTCCGCCTGGCCAAG
GACCTGGCGGAGAACAGCCGCGGCGCCCGGGTGCTCGTGGTCTGCTCCGAGATCACTGCTGTCACCTTCCGCGGG
CCCTCGGAGTCTCACCTCGACAGCCTCGTGGGGCAGGCTCTGTTCGGCGACGGCGCCGCCGCCATCATCGTCGGC
GCAGACCCGGACCCCGTCACGGAGCGCCCTATCTTCCAGCTCGTCTCCGCCAGCCAGACCATCCTCCCCGACTCC
GAAGGCGCCATCGACGGCCACCTGCTGGAGGTCGGCCTCACCTTCCACCTGCTCAAGGACGTGCCCGGCCTCATC
TCCAAGAATATCGAGAGGAGCCTAGTGGAGGCGTTCAAGCCGCTGGGGATCAGCGACTGGAACTCCATGTTCTGG
ATCGTGCACCCCGGCGGACCAGCGATCCTCGACCAGGTGGAGGCCAAGCTGGGCCTTGAGAAGGAGAAGATGAAG
GCGACGCGGCAGGTGCTGAGCGAGTACGGGAACATGTCCAGCGCATGCGTGCTCTTCATCCTCGACGAGATGAGG

AAGCGATCCGCCGAGGACGGAAAGGCGACCACCGGGGAGGGGCTGGAGTGGGGGGTGCTCTTCGGGTTCGGCCCG
GGGCTGACGGTGGAGACGGTAGTCTTGCACAGCATCCCCATCGCTGCCCGATGA

>GSMUA_Achr6P16370_001_(MaCHS4)_Protein

MAKVQEIRSSQRAEGPATVLAIGTATPANVVYQAEYPDYYFRITKSEHLTELKEKFKRMCDKSMIRKRYMHLNEE
ILQENPNMCAYMAPSLDARQDIVVVEVPKLGKEAAAKAIKEWGQPKSKITHLVFCTTSGVDMPGADYQLTKLLGL
RPSVNRFMMYQQGCFAGGTVLRLAKDLAENSRGARVLVVCSEITAVTFRGPSESHLDSLVGQALFGDGAAAIIVG
ADPDPVTERPIFQLVSASQTILPDSEGAIDGHLLEVGLTFHLLKDVPGLISKNIERSLVEAFKPLGISDWNSMFW
IVHPGGPAILDQVEAKLGLEKEKMKATRQVLSEYGNMSSACVLFILDEMRKRSAEDGKATTGEGLEWGVLFGFGP
GLTVETVVLHSIPIAAR

>GSMUA_Achr10T12200_001_(MaCHS5)_Genomic

AGCTGACGGGAGGAGGAAGCTGGTGACCCGCGTGGCGTTCACTGTGCCTAGCCTCCCACCAAACCTTCTCTGCTC
CTCGTGGCTTCACATCTGTCCGTCCGTTCAACCTAACCAGCCACCGCTATATATCTCCCGCCTCCACCCCCTCTG
CCTCCTCATCTCCGGACCGAACCATCAACTCTTCGATCGACCTAGTACTTGGTCCTACACCCACTTCTAGCTCAT
GGCCAAGTTGGCAGAGATCAGGCAATCGCAGAGGGCAGAGGGTTCGGCGACGGTGCTCGCCATCGGCACCGCCAC
CCCTGTCAACGTCTTGTACCAGGCCGACTACCCAGACTACTACTTCCGCATCACCAAGAGCGAGCACCTCACTGA
ACTCAAGGAAAAGTTCAAGAGAATGTGTACGTGCCGTTTCTTTAATCTAAAGTGCCATTCATTTCTTTAGGTTCC
TTCTTCTCTAACAAATAAATGGGACCTCCGCCTTTATCTATGTTAGCAGAGAGACGAAAAAGAAAAGGTCTCCTC
TGACTGGTTTAGTTGTTCCTGCGATGATCACAACTGGTTCAATAAATAAAAAGTATAAAACATGCACATGATACG
TGAATTCACATGACATAGTTAATAAAAAAGACGATGTTGTAGTTGTGTAATCGTGATCTTTGCACTACATGCTGG
TTCAAGGGACAAGTGTGTGATCCAAAGATGAAGTCTTGGTTGTGGAGTTGTGATCCCTGTACTGCATGCTTGTTC
AGGTGACAAGTCGATGATCCGCAAACGTTACATGCACATCAACGAGGAGATCCTGAAGGAGAACCCTAACGTCTG
CGCGTACATGGCGCCGTCGCTGGACGCTCGGCAGGACATCGTGGTGGTGGAGGTGCCGAAGCTAGGCAAGGAGGC
GGCTGTCAAGGCCATCAAGGAATGGGGGCAGCCCAAGTCCAAGATCACCCATCTCGTCTTCTGCACCACCAGCGG
CGTCGACATGCCTGGCGCTGACTACCAGCTTACCAAACTGCTTGGCCTCCGACCCTCCGTCAACCGGTTCATGAT
GTACCAGCAGGGCTGCTTCGCGGGCGGCACGGTGCTCCGCATGGCCAAGGACCTGGCCGAGAATAACCGCGGCGC
CCGGGTGCTCGTGGTCTGCTCCGAGATCACCGCTGTCACCTTCCGCGGGCCCTCGGAGTCCCACCTCGACAGCCT
TGTCGGGCAGGCCCTGTTCGGCGACGGCGCCGCGGCCATCATCGTCGGCGCTGACCCCGACCCCGCCACAGAGAA
GCCGATTTTCCAGCTCGTCTCCGCCAGCCAGACCATCCTCCCCGACTCCGAGGGCGCCATCGACGGCCACCTGCG
GGAGGTCGGCCTCACCTTCCATCTGCTCAAGGACGTGCCGGGCCTCATCTCCAAGAACATCGAGCGGAGCCTGGC
GGAGGCGTTCAAGCCGCTGGGGATCAGCGACTGGAACTCCATATTCTGGATCGCGCACCCCGGCGGGCCGGCGAT
CCTCGACCAGGTGGAGGCCAAGCTCGGCTTGGAGAAGGAAAAGATGAAGGCAACGCGGGAGGTGCTGAAAGAGTA
CGGGAACATGTCGAGCGCCTGCGTGCTGTTCATCCTGGACGAGATGAGGAAGCGGTCGGCCGAGGACGGGAAGGC
GACCACCGGCGAGGGGCTTGAGTGGGGCGTCCTCTTCGGGTTCGGTCCCGGGCTCACGGTGGAGACCGTGGTCTT
GCACAGCATCCCCATCGCTGCTCACTGACGCAAGCTTTTCCTTCCATCACGTGAATCTCCCTGTAACTGGTTGTG
TGGCTACTGCTATTGATATCCGTCGGTGTGGATTTATTTGGCTATGAAGTTGTCTGTAACTACAACATCAAACCC
TACTATTTATCGTGCTGAAGGCGTGGGAGAAAACGTTATCTAGTGTTACGTTCTATGGTGCTTAATAAACTCCGT
TTCCTCAGACACGTTGTTGT

>GSMUA_Achr10T12200_001_(MaCHS5)_Modified CDS

ATGGCCAAGTTGGCAGAGATCAGGCAATCGCAGAGGGCAGAGGGTTCGGCGACGGTGCTCGCCATCGGCACCGCC
ACCCCTGTCAACGTCTTGTACCAGGCCGACTACCCAGACTACTACTTCCGCATCACCAAGAGCGAGCACCTCACT
GAACTCAAGGAAAAGTTCAAGAGAATGTGTGACAAGTCGATGATCCGCAAACGTTACATGCACATCAACGAGGAG
ATCCTGAAGGAGAACCCTAACGTCTGCGCGTACATGGCGCCGTCGCTGGACGCTCGGCAGGACATCGTGGTGGTG
GAGGTGCCGAAGCTAGGCAAGGAGGCGGCTGTCAAGGCCATCAAGGAATGGGGGCAGCCCAAGTCCAAGATCACC
CATCTCGTCTTCTGCACCACCAGCGGCGTCGACATGCCTGGCGCTGACTACCAGCTTACCAAACTGCTTGGCCTC
CGACCCTCCGTCAACCGGTTCATGATGTACCAGCAGGGCTGCTTCGCGGGCGGCACGGTGCTCCGCATGGCCAAG
GACCTGGCCGAGAATAACCGCGGCGCCCGGGTGCTCGTGGTCTGCTCCGAGATCACCGCTGTCACCTTCCGCGGG
CCCTCGGAGTCCCACCTCGACAGCCTTGTCGGGCAGGCCCTGTTCGGCGACGGCGCCGCGGCCATCATCGTCGGC

GCTGACCCCGACCCCGCCACAGAGAAGCCGATTTTCCAGCTCGTCTCCGCCAGCCAGACCATCCTCCCCGACTCC
GAGGGCGCCATCGACGGCCACCTGCGGGAGGTCGGCCTCACCTTCCATCTGCTCAAGGACGTGCCGGGCCTCATC
TCCAAGAACATCGAGCGGAGCCTGGCGGAGGCGTTCAAGCCGCTGGGGATCAGCGACTGGAACTCCATATTCTGG
ATCGCGCACCCCGGCGGGCCGGCGATCCTCGACCAGGTGGAGGCCAAGCTCGGCTTGGAGAAGGAAAAGATGAAG
GCAACGCGGGAGGTGCTGAAAGAGTACGGAACATGTCGAGCGCCTGCGTGCTGTTCATCCTGGACGAGATGAGG
AAGCGGTCGGCCGAGGACGGGAAGGCGACCACCGGCGAGGGGCTTGAGTGGGGCGTCCTCTTCGGGTTCG
GTCCCGGGCTCACGGTGGAGACCGTGGTCTTGCACAGCATCCCCATCGCTGCTCACTGA

>GSMUA_Achr10P12200_001_(MaCHS5)_Protein

MAKLAEIRQSQRAEGSATVLAIGTATPVNVLYQADYPDYYFRITKSEHLTELKEKFKRMCDKSMIRKRYMHINEE
ILKENPNVCAYMAPSLDARQDIVVVEVPKLGKEAAVKAIKEWGQPKSKITHLVFCTTSGVDMPGADYQLTKLLGL
RPSVNRFMMYQQGCFAGGTVLRMAKDLAENNRGARVLVVCSEITAVTFRGPSESHLDSLVGQALFGDGAAAIIVG
ADPDPATEKPIFQLVSASQTILPDSEGAIDGHLREVGLTFHLLKDVPGLISKNIERSLAEAFKPLGISDWNSIFW
IAHPGGPAILDQVEAKLGLEKEKMKATREVLKEYGNMSSACVLFILDEMRKRSAEDGKATTGEGLEWGVLFGFGP
GLTVETVVLHSIPIAAH

>GSMUA_Achr10T12260_001_(MaCHS6)_Genomic

TTGCTTGTCGGAGGGGTCTCGTCGAGTGATCCCGACGAGGGTCATTTTTGCAGGAAAACTCGGCCACCTCACAAA
GGCGAGCTGCCAGCCACCCCGAAGAAGGAGAGACGTAACTCAGCATACACGGTGCCCGGATCGGCATACAAAGAA
TGTTGATCAATACCCCGTCGTGAGAGTCTGATCGACCTCGGCATCCAGCTCAATCAACAGAAGACTCCCGACATT
GACCCATGGACCAAGCCGTGCTGACTCACCAGCAACATCACATCTGTTCACCAACAAATATCATTCGGTACGACC
TAATCATCCACGACACCATCGGAAAAAGAAAATAAACAGTAATAATAACCAGACTCAGCATGTTCGAAAGAGACA
TATCGAGATCGGCGAGCGTACCAGCTCGTCTAAGCCTTCGGCGGCGATGGAAACCCGTTGTCGCGGGGTTAAGAG
CGGCAACGGGAGACGGATCCCCAAATTGAATGGAAATTATATGGAACATAAACGTATTTCAGCCGTAGGATCGGG
CTACGAGCGACCGCACGCGTTAATCGGACTTCATCAAGAGTATGGAAAACATGTGGGATCACAGCACAGGTTGAC
ACGTGCTGTGCACGGACCAGCGGCCTGTGACGCGCTTCGGGGCCGGCGCACTCCAGCCTGTATCGGCTCGTTGTG
AAGCTTACTTATGTTAAAAGCAAAATTAATATTAATTTTGTAATGTCTTCTAAAGTTCTATGAAAAATACGTGTA
ATTTTAAAAATATATGTTTTATGTTTAAATTATGATGATTGTATCATGTTGGAATTTCTAATTAACATTTTTTCT
CATTTCAAAAAATTCAATGATATGCAATGTTCAACAAAAAAACAATACTTTTCGCGAGTACACAAACAGTTAAAA
TGGTTCCGACCAAATCTCTTGAAAAAAAAAAAAAAATCTCATAGTACACAAAGAGTGGAAGAACAGTAAATCAAA
TTATAATATTATTTATGAACGAATGCGACACCAATTGTGATGAAATAGAAGGTAGGTAGCAAAATTAGGTACGGA
GGACCAGTTGTTATAGTTAAATTAAGTAGCTTACCAAGTCTTTCATTTGTGGGTGGCTAAAGTTCTGGCTCCTGT
CGGGTCTCCCACATTCTTCATCAAAAGCACCTGGCAACCACCTGACACTTCCAACGTCATGAATTATCTGGCCTT
GGGCAGTCATGGGTGATTTGGCGTCCAAGGCGGATTAACCAAGTATGCGTCGTCCAAGTCATTTGACTAAGTGGA
TGCGGAGGATTTATTTCAAGGCCCACTTAGCAGATTTTACGTATGAACGACGGACGCGACGCACACCGGTGGAAG
GGAGGCGAAGTGCCTTCCCCTTGCGCGGTGTCACTGCTTCTCGAGTCTCGATCCGACCCTTCACGGCGAAGTGCA
TCTTCCGAGATCAGAGAAAGAGGGGCTCTTCCTCTACCCGTTGACGGGGCGCTCGATCGCCTTCTGGTCTGCGAC
AACCAGAAGGCCACGAGGTGTGCGCGCGGCAGATGAGAGATGAATAAATGCATGGAGTCGGAGTTTACAGGTTAA
GTTTTCAGCTGACGGAAAGAGGAGGTGGTCACCCGCATGTTGGTCACTGTGCCTAGCCTCCCACCAATCATTCTC
TGCTCCTCGTCGCTTCCCATCTGCTCGTCCGTTCAACCCAACCAGCCACCGCTATATATCTCCCGCCTCCTCATC
TCCGGACCGAACCATCAACTCTTCGATCGACCTAATACTTGGTCCTGCTCCCACTTCTAGCTCATGGCCAAGTTG
GCGGAGATCAGGCAATCGCAGAGGGCAGAGGGTTCGGCGACGGTGCTTGCCATCGGCACCGCCACCCCTGTCAAC
GTCATGTACCAGGCCGACTACCCGGACTACTACTTTCGCATCACCAAGAGCGAGCACCTCACTAAACTCAAGGAA
AAGTTCAAGAGAATGTGTACGTGCCGTTTCTTTAATCTAAAGTGCCATTCATTTCTTAGGTTCCCTCTTCCCTAA
CAAATAAATTCGACCTGCGCCTTTATATTTGTTACCAGAGAGACGAAAAAGAAAAGGCCTCCTCTGACTGGTTTA
GTTGTTCCTGCGATAATTACAACTGTTCAATAAATAAAAGAATAAAACATGAACATGATACGTGAATTCGCATGA
CATAGTTAATAAAAGAGACGAAGTTGTTCTTGTGTAATCGTGGTCTTTGCACTGCATGCTGGTTCGAGGGACAAG
TGTGTGATCCAAAGATGAAGTCCTAGTTTTGATCCCTGTACTGCATGCTTGTTCAGGTAACACGTCGATGATCCG
CAAACGTTATCTGCACCTCAACGAGGAGATCCTGAAGGAGAACCCTAACATCTGCGCGTACATGGCGCCGTCGCT
GGACGCTCGGCAGGACATCGTGGTGGTGGAGGTGCCGAAGCTAGGCAAGGAGGCGGCGGTCAAGGCCATCAAGGA

GTGGGGGCAGCCCAAGTCCAAGATCACCCATCTCGTCTTCTGCACCACCAGCGGCGTCGACATGCCTGGCGCCGA
CTTCCAGCTTACCAAACTCCTTGGGCTCCGACCCTCCGTCAACCGGTTCATGATGTACCAGCAGGGCTGCTTCGC
GGGCGGCACGGTGCTCCGCATGGCCAAGGACCTGGCCGAGAATAACCGCGGCGCCCGGGTGCTCGTGGTTTGCTC
CGAGATCACCGCTCTCAGCTTCCACGGGCCCTCGGAGTCCCACCTCGACAACCTTGTTGGGCAGGCCCTGTTCGG
CGACGGCGCCGCCGCCATCATCGTCGGCGCCGACCCCGACCCCGCAACGGAGCAGCCGATTTTCCAGCTAGTCTC
CGCCAGCCAGACCCTCCTCCCCGACTCCGAGGGCGCCGTCGACGGCCACCTGCGGGAGGTCGGCCTCATCTTCCA
TCTGCTCAAGGACGTGCCGGTCCTTATCTCCAAGAACATCGAGCGGAGCCTGGTGGAGGCATTCAAGCCGCTGGG
GATCAGCGACTGGAACTCCATATTCTGGATTGCGCACCCCGGAGGGCCCGCGATCCTCGACCAGGTAGAGGCCAT
GCTCGGCTTGGAGAAGGAGAAGATGAAGGCTACGCGGGAGGTGCTGAAAGAGTACGGGAACATGGCGAGCGGCAC
CGTGCTGTTCATCCTGGACGAGATGAGGAAACGGTCGGCCGAGGACGGAAAGGCGACCACCGGCGAGGGGCTGGA
GTGGGGCGTCCTCTTCGGGTTCGGTCCCGGGCTCACGGTGGAGACTGTGGTCTTGCACAGCATCCCCATCGCTGA
CAAGTGACGCAAGCTTTTCCTTCCATCACGTGAATCTCCCTGTAACTGGTTGTGTGGCTACTGCTATTGATATCC
GTCGGTGTGGATTTATTTGGCTATGAAGTTGTCTGTAACTACAACATCAAACCCTACTATTTATCGTGCTGAAGG
CGTGGGAGAAACGTCATGTAGTGTTATGTTCCATTGTGCTTAATAAACTCTGTTTCGTCAAACACGTTATTGTCG
TACTTCCTCAATGTACGTTTCTATGTGGGTCCCATAGTTCAAGCCTACAAGAGAGCCACGAAGGACGAGGAAGTC
TATGGGTTAGGGAACCTCCGCAAGTGTTTTTGAACCAAATATATATACTCAGTGTAATTT

>GSMUA_Achr10T12260_001_(MaCHS6)_Modified CDS

ATGGCCAAGTTGGCGGAGATCAGGCAATCGCAGAGGGCAGAGGGTTCGGCGACGGTGCTTGCCATCGGCACCGCC
ACCCCTGTCAACGTCATGTACCAGGCCGACTACCCGGACTACTACTTTCGCATCACCAAGAGCGAGCACCTCACT
AAACTCAAGGAAAAGTTCAAGAGAATGTGTAACACGTCGATGATCCGCAAACGTTATCTGCACCTCAACGAGGAG
ATCCTGAAGGAGAACCCTAACATCTGCGCGTACATGGCGCCGTCGCTGGACGCTCGGCAGGACATCGTGGTGGTG
GAGGTGCCGAAGCTAGGCAAGGAGGCGGCGGTCAAGGCCATCAAGGAGTGGGGGCAGCCCAAGTCCAAGATCACC
CATCTCGTCTTCTGCACCACCAGCGGCGTCGACATGCCTGGCGCCGACTTCCAGCTTACCAAACTCCTTGGGCTC
CGACCCTCCGTCAACCGGTTCATGATGTACCAGCAGGGCTGCTTCGCGGGCGGCACGGTGCTCCGCATGGCCAAG
GACCTGGCCGAGAATAACCGCGGCGCCCGGGTGCTCGTGGTTTGCTCCGAGATCACCGCTCTCAGCTTCCACGGG
CCCTCGGAGTCCCACCTCGACAACCTTGTTGGGCAGGCCCTGTTCGGCGACGGCGCCGCCGCCATCATCGTCGGC
GCCGACCCCGACCCCGCAACGGAGCAGCCGATTTTCCAGCTAGTCTCCGCCAGCCAGACCCTCCTCCCCGACTCC
GAGGGCGCCGTCGACGGCCACCTGCGGGAGGTCGGCCTCATCTTCCATCTGCTCAAGGACGTGCCGGTCCTTATC
TCCAAGAACATCGAGCGGAGCCTGGTGGAGGCATTCAAGCCGCTGGGGATCAGCGACTGGAACTCCATATTCTGG
ATTGCGCACCCCGGAGGGCCCGCGATCCTCGACCAGGTAGAGGCCATGCTCGGCTTGGAGAAGGAGAAGATGAAG
GCTACGCGGGAGGTGCTGAAAGAGTACGGGAACATGGCGAGCGGCACCGTGCTGTTCATCCTGGACGAGATGAGG
AAACGGTCGGCCGAGGACGGAAAGGCGACCACCGGCGAGGGGCTGGAGTGGGGCGTCCTCTTCGGGTTCGGTCCC
GGGCTCACGGTGGAGACTGTGGTCTTGCACAGCATCCCCATCGCTGACAAGTGA

>GSMUA_Achr10P12260_001_(MaCHS6)_Protein

MAKLAEIRQSQRAEGSATVLAIGTATPVNVMYQADYPDYYFRITKSEHLTKLKEKFKRMCNTSMIRKRYLHLNEE
ILKENPNICAYMAPSLDARQDIVVVEVPKLGKEAAVKAIKEWGQPKSKITHLVFCTTSGVDMPGADFQLTKLLGL
RPSVNRFMMYQQGCFAGGTVLRMAKDLAENNRGARVLVVCSEITALSFHGPSESHLDNLVGQALFGDGAAAIIVG
ADPDPATEQPIFQLVSASQTLLPDSEGAVDGHLREVGLIFHLLKDVPVLISKNIERSLVEAFKPLGISDWNSIFW
IAHPGGPAILDQVEAMLGLEKEKMKATREVLKEYGNMASGTVLFILDEMRKRSAEDGKATTGEGLEWGVLFGFGP
GLTVETVVLHSIPIADK

>GSMUA_Achr4T16830_001_(MaCHI1)_Genomic

AGACCCTACGAAGAACGGCAGGTTCGAGTGTCGGCTCGGACCTAGCGGTGTAGAATATTGTTGGCTGC
AGCGTGATCAGGTTTTAAATATTGTAGGTGCAAAATTGATCGACGAAGCCAAACACTCTGGAAGTTCT
TGTAAGGTGTGACGTGCGGCCGGTTCGAGGAATCTCCGGTCATCGGTGGCACAATGGTTATTGTGAGC
TAAAGCTGTGGGTGTTTATTTATCTTAATCTGCGCCAGTCAGTGCTCTGTCTATATAGTCGGGATCCT
CCTTCCATCTCGAATCACTTCGACGGCGAAGAAGAGGCCGAAGCATAAAATGAGCGGTGGAACAGGGT
CTCCGTTGCCCATGCTCGAGGTCGAAGGCGTCGTCTTCCCTCCGGTGGTCACGCCACCCGGTTCCACG
AAGGCCCACTTCCTCGGTGGCGCAGGTCTGCTCCACCCTCGGCAGTACTTGCTCTCCCCGTCTTGGCT
GCTGACGTGAACCGGTGTCACGCAGGTGTTCGGGGCCTGGAGATCGAAGGCAGGTTCGTCGCCTTCAC
GGCCATCGGCGTCTACCTCGAGGATGCTGCGGTGCAGTCGCTTGCCAGCAAGTGGAAGGGGAAGAGTG

CCGACGAGCTCGATGGCGCCGTCGAATTTTTCCGAGACATCTTCGGAGGTCAGACGACTTTATATATA
TATATATATATATATATATATATATATATATATATATATATCGCAGAAGGAAGCGGATCGTACGGGCA
TTAGAGTCGTTATTCTTGTGAAGCACATGAGATATTAGCATAAATATTTAGTATAGAATTCTCGCGTT
GGTTCTGAAATCCATGGAGACAGGTCCATTTGAGAAATTCACGAGAGTGACGCTGCTGAAGCCACTGA
CCGGGCAGCAGTACGCGGAGAAGGTGTCAGAGAACTGCACTGCACAGTGGAAGGCGGCCGGAGTCTAC
GCCGAAGCAGATGGCGCGGCCTTGGAGCAGTTCAAAGAGGCTTTCAGGGCCGAGACCTTCCCTCCCGG
CTCCTCCATTCTCTTCACTCATGCCCCTTCTGACTCTCTCCTTGTAGGTTCTCTCTCTCTCTCTCTCT
GCCCGCACATCTCTCATTCCCTTCGTATGTCCTACATCTCATTCTCTTCTTACGATCATGCTGCAGAT
CGCATTCTCCAAGGACGGGTCGATGCCGGAGGCAGGGATCGCCATGATACAGAACCAACCTCTCAGCC
AGGGGATTTTGGAGTCCATCATCGGTGAGAATGGCGTCTCCCCTGGAGCGAAAAGGAGCCTGGCACTG
CGGTTCTCGGAGCTCCTGAAGCAACACTGCGAGGCAGAAGAGACCAAGCTGGTGAATCCTGTAGCTGT
TATCGTCTAAGCGAGTCTGATCGTCTAAAAGGAGTCAGTCCAGTCTGAGTGGCAGTGATGGCCTCATG
TCTGAGGCCATGCTTGAGTTCTCTTTACATTTCTTTCAGAAAGTAGAACTTCATTAAGTAATAAAGAA
CACTAGTCTACCTATGTGTACGGTTAAGTGCTCAAATTTCATCATGGGTAATCATATTGTCAGATTAA
TTTACAATGATGAAGAGAGATCAATAGGCAATTTACGCTTGGACGAAGAAAATAAATCGAGGATTAAA
GTGACTTGTTGATATGCAACCTGACAGAACATAGCTCGCGAATAGCTTTTCTCCTCTG

>GSMUA_Achr4T16830_001_(MaCHI1)_Modified CDS

ATGAGCGGTGGAACAGGGTCTCCGTTGCCCATGCTCGAGGTCGAAGGCGTCGTCTTCCCTCCGGTGGT
CACGCCACCCGGTTCCACGAAGGCCCACTTCCTCGGTGGCGCAGGTGTTCGGGGCCTGGAGATCGAAG
GCAGGTTCGTCGCCTTCACGGCCATCGGCGTCTACCTCGAGGATGCTGCGGTGCAGTCGCTTGCCAGC
AAGTGGAAGGGGAAGAGTGCCGACGAGCTCGATGGCGCCGTCGAATTTTTCCGAGACATCTTCGGAGG
TCCATTTGAGAAATTCACGAGAGTGACGCTGCTGAAGCCACTGACCGGGCAGCAGTACGCGGAGAAGG
TGTCAGAGAACTGCACTGCACAGTGGAAGGCGGCCGGAGTCTACGCCGAAGCAGATGGCGCGGCCTTG
GAGCAGTTCAAAGAGGCTTTCAGGGCCGAGACCTTCCCTCCCGGCTCCTCCATTCTCTTCACTCATGC
CCCTTCTGACTCTCTCCTTATCGCATTCTCCAAGGACGGGTCGATGCCGGAGGCAGGGATCGCCATGA
TACAGAACCAACCTCTCAGCCAGGGGATTTTGGAGTCCATCATCGGTGAGAATGGCGTCTCCCCTGGA
GCGAAAAGGAGCCTGGCACTGCGGTTCTCGGAGCTCCTGAAGCAACACTGCGAGGCAGAAGAGACCAA
GCTGGTGAATCCTGTAGCTGTTATCGTCTAA

>GSMUA_Achr4P16830_001_(MaCHI1)_Protein

MSGGTGSPLPMLEVEGVVFPPVVTPPGSTKAHFLGGAGVRGLEIEGRFVAFTAIGVYLEDAAVQSLAS
KWKGKSADELDGAVEFFRDIFGGPFEKFTRVTLLKPLTGQQYAEKVSENCTAQWKAAGVYAEADGAAL
EQFKEAFRAETFPPGSSILFTHAPSDSLLIAFSKDGSMPEAGIAMIQNQPLSQGILESIIGENGVSPG
AKRSLALRFSELLKQHCEAEETKLVNPVAVIV

>GSMUA_Achr11T23630_001_(MaCHI2)_Genomic

ACTTGTGAGAGGATTTAGATGCTGAAAGAAACACATGCGTCGCTAAAAAGCCAAGAAATAAACGCTCA
TTATGTTCATGGACACCTCGAAACCCAACTAACCTATTGGGTGGTCACAAGTTGACGTTCTGGTACCT
ACCACCACGCAGGGCAGCAGCAGCAGCAGCATTCATGTCTGCAGCAGTCGCATTTATCGTCCGCTGCG
AGCAGAGTAGTTAGCACAAGCCTTTCACCCAACTACCACCTCTCTTCTTAAGACCCGTTCTTCACCCT
TCTCTCCTCCTCAGACACTGGTCTCCACCTCTCCTGCTTTCTCCTACTCCCCTCCTTCGGGTTCTCGT
CTTATTGTGTTCATCAGCGAGTAATTATTCTTCCTGTTTTCTTGGTTTCCAGAACGATTATTAGTACT
TGATCGCTTTGACATTGGCACTCTGTATATGGCAGTGGGATCGGAGACGGTGATGGTGGACCAAGTCC
CATTCGCTCCTGAGATCACGGTCACCAAACCATTAAGCCTCCTGGGCTATGGTAAGTCGGCACTCATT
TCGACCCATCATCCCTAACTTTCGACCCATTAAGCCTCCTGTACTCTTGTTGTTCTTGTGACGCAGGC
GTCACCGACATCGAGATTCATTTTCTACAGATCAAGTATAACGCGATTGGGATCTACATGGAGAAGGA
GATCATTCAGCATTTAGCGGATTGGAAGGGCAAAAAGGGATCCGAGCTAGCCGAAGATGATGTCTTCT
TTGATGCTGTCGTTTCAGGTGACAATTACATATGACCATTAGGTCAGAGCAAGATATGGCTCCCACAG

TCGGGTCTTTCTTGAAGGTTTGCAGTTCCTTGTATGCTTGCTGCAGACTATTTATCTTGATGACTCTA
CGTCGTCATGTGCAGCTCCAGTCGAAAAATACTTCAGAATCGTGGTGATTAAGGAGATCAAAGGTTCC
CAGTACGGTGTGCAACTCGAGAGCGCTGTGAGAGACAGATTGGCAGCCATCGACAAGTACGAAGAGGA
AGAAGAGGAAGCACTCGAAAAGGTCACCGAGTTTTTCCAGAAGAAGTACTTCAAGAAGGATTCTCTGA
TAACGTTTCATTTCCCTGCAGCTTCTCGCACTGCTGAGGTAAGTATCAGATCGGGATACACATATATA
ACCCTTCTCAACGCTGAGGAAAGAAGAAGAAAGAGGCATCAAGCCGAAACTGATATGTTTGGGTTTTC
CCTCTGCAGATATCCTTTGCGACGGAGGGAAAGGAGGAAGCGAAGGTGAAGGTGGAGAATCCAAACGT
GGCTGAGATGATCCAGAAATGGTACTTGGGTGGGACTAGGTCGGTGTCTCCAACCACTGTGAAGAGCT
TGGCTGACAACCTTGGGACGATGCTCGCTCAGTAGCTCTTTGGGTGACTAGTTTCTCGTA

>GSMUA_Achr11T23630_001_(MaCHI2)_Modified CDS

ATGCTGAAAGAAACACATGCGTCGCTAAAAAGCCAAGAAATAAACGCTCATTATGTTCATGGACACCT
CGAAACCCAACTAACCTATTGGGTGCACAAGCCTTTCACCCAACTACCACCTCTCTTCTTAAGACCCG
TTCTTCACCCTTCTCTCCTCCTCAGACACTGGTCTCCACCTCTCCTGCTTTCTCCTACTCCCCTCCTT
CGGGTTCTCGTCTTATTGTGTTCATCAGCGATGGGATCGGAGACGGTGATGGTGGACCAAGTCCCATT
CGCTCCTGAGATCACGGTCACCAAACCATTAAGCCTCCTGGGCTATGGCGTCACCGACATCGAGATTC
ATTTTCTACAGATCAAGTATAACGCGATTGGGATCTACATGGAGAAGGAGATCATTCAGCATTTAGCG
GATTGGAAGGGCAAAAAGGGATCCGAGCTAGCCGAAGATGATGTCTTCTTTGATGCTGTCGTTTCAGC
TCCAGTCGAAAAATACTTCAGAATCGTGGTGATTAAGGAGATCAAAGGTTCCCAGTACGGTGTGCAAC
TCGAGAGCGCTGTGAGAGACAGATTGGCAGCCATCGACAAGTACGAAGAGGAAGAAGAGGAAGCACTC
GAAAAGGTCACCGAGTTTTTCCAGAAGAAGTACTTCAAGAAGGATTCTCTGATAACGTTTCATTTCCC
TGCAGCTTCTCGCACTGCTGAGATATCCTTTGCGACGGAGGGAAAGGAGGAAGCGAAGGTGAAGGTGG
AGAATCCAAACGTGGCTGAGATGATCCAGAAATGGTACTTGGGTGGGACTAGGTCGGTGTCTCCAACC
ACTGTGAAGAGCTTGGCTGACAACCTTGGGACGATGCTCGCTCAGTAG

>GSMUA_Achr11P23630_001_(MaCHI2)_Protein

MLKETHASLKSQEINAHYVHGHLETQLTYWVHKPFTQLPPLFLRPVLHPSLLLRHWSPPLLLSPTPLL
RVLVLLCSSAMGSETVMVDQVPFAPEITVTKPLSLLGYGVTDIEIHFLQIKYNAIGIYMEKEIIQHLA
DWKGKKGSELAEDDVFFDAVVSAPVEKYFRIVVIKEIKGSQYGVQLESAVRDRLAAIDKYEEEEEEAL
EKVTEFFQKKYFKKDSLITFHFPAASRTAEISFATEGKEEAKVKVENPNVAEMIQKWYLGGTRSVSPT
TVKSLADNLGTMLAQ

>GSMUA_Achr2T02770_001_(MaF3H1)_Genomic

CTCTCCCCCTCGTTAAAAGCACATGGAGATGCCTCACCATCCCAATCCAGCTTAAAGTGCAGGCAGCA
CCAGAGATCGAAGATGGCTCCCGGCACCGCAGTCCTTCCCACCAACGAGCAGACCTTGAGGGCGAGCT
TCGTCCGGGACGAGGACGAGCGTCCCAAGGTGGCTTACAACCAGTTCAGCCTTGACATCCCCGTGATC
TCGCTCTCCGGGATTGATGACGACGCCGCGGGCGGGAAGAGGGCGGAGATCCGCCGCAAGGTCGTGGA
GGCCTGCGAGGACTGGGGTATATTCCAGGTGGTGGACCACGGGGTCGACGCCGGCCTTATCTCCGACA
TGACTAGGCTGGCCAAAGAGTTCTTCGCGCTGTCCCCGGAGGAGAAGCTCTTGTTCGACATGTCCGGT
GGGAAGAAGGGAGGGTTCATCGTCTCCAGCCACCTCCAGGTATACCAGTCACCGCTCTCGGATACTCG
ATTCGCATGCATATGAATGAGGCCGTTTACTTGATGTGTTGCTCAGGGCGAGGCAGTTCAGGACTGGA
GAGAGATCGTGACGTACTTCTCATACCCAATTCGGGCTCGGGACTACTCGCGATGGCCGGACAAGCCG
GAAGGGTGGAGATCCGTGGTGGAGTCGTACAGCGAGAAGCTTATGGGCTTGGCGTGCAAGCTCTTGGA
GGTGTTGTCTGAGGCTATGGGGCTCGACAAGGAGGCCCTCACCGAGGCCTGCATCGACATGGATCAGA
AGGTTGTCGTCAACTACTACCCCAAGTGCCCCCAGCCGGACCTCACTCTTGGCCTCAAACGACACACC
GATCCCGGCACCATCACCCTCCTCCTCCAAGACCAAGTCGGCGGCCTCCAAGCCACCAAGGATGGCGG
CAAGACATGGATCACGGTTCAGCCTGTGGAGGGAGCTTTTGTGGTCAACCTCGGGGACCATGGCCATG
TGAGTGCAGCTCCTCATCAAGTCAGCTGACTGTCTCTCTTTATGATCTCTTCAAAGAAGAGCCAGAAT
CTAAATCCCCTTTTTCTGAGGCAGTTTCTGAGCAATGGGAGGTTCAAGAATGCTGACCACCAGGCTGT
GGTGAACTCAAACTACAGCAGGCTCTCCATCGCCACGTTTCAGAACCCCGCGCCGGAGGCAATCGTGT
ATCCGCTGGCAGTAAGGGAGGGAGAGAAGCCGATTCTGGAGGAGCCCATCACGTTTGCCGAGATGTAC

CGCAGGAAGATGAGCCGAGACCTCGAGCTCGCCAAGCTCAAGAAACTGGCCAAGACGGAGCAGAAGCA
GCAGCCGGAGCTACTGGAGAAGACTAAGGACATCAACTTGGCCAAGGCTACAGGCCTCGATGAGATCT
TGGCTTAATACATATGTGTAGGTCTTTGTCATCTTCCATGTCTGTCCTACTTTAGCTTCCTTGTCTGG
TTTGTTGGTCGAAGCTGTCATAAGTTTTCCCGTGATATGGACTATGAATCTATCATCCTTTGTTATTT
CACTTTGTTTGATAAGATCTGCTGTGCCTTTCTGCATCTTCGTATATAAAGAACATTCAAGGAAAACT
GAAGGAATTCTTTCTGAATCAAGGATAGAAATCTATGGAATTCGAGCATATATCCT

>GSMUA_Achr2T02770_001_(MaF3H1)_Modified CDS

ATGGCTCCCGGCACCGCAGTCCTTCCCACCAACGAGCAGACCTTGAGGGCGAGCTTCGTCCGGGACGA
GGACGAGCGTCCCAAGGTGGCTTACAACCAGTTCAGCCTTGACATCCCCGTGATCTCGCTCTCCGGGA
TTGATGACGACGCCGCGGGCGGGAAGAGGGCGGAGATCCGCCGCAAGGTCGTGGAGGCCTGCGAGGAC
TGGGGTATATTCCAGGTGGTGGACCACGGGGTCGACGCCGGCCTTATCTCCGACATGACTAGGCTGGC
CAAAGAGTTCTTCGCGCTGTCCCCGGAGGAGAAGCTCTTGTTCGACATGTCCGGTGGGAAGAAGGGAG
GGTTCATCGTCTCCAGCCACCTCCAGGGCGAGGCAGTTCAGGACTGGAGAGAGATCGTGACGTACTTC
TCATACCCAATTCGGGCTCGGGACTACTCGCGATGGCCGGACAAGCCGGAAGGGTGGAGATCCGTGGT
GGAGTCGTACAGCGAGAAGCTTATGGGCTTGGCGTGCAAGCTCTTGGAGGTGTTGTCTGAGGCTATGG
GGCTCGACAAGGAGGCCCTCACCGAGGCCTGCATCGACATGGATCAGAAGGTTGTCGTCAACTACTAC
CCCAAGTGCCCCCAGCCGGACCTCACTCTTGGCCTCAAACGACACACCGATCCCGGCACCATCACCCT
CCTCCTCCAAGACCAAGTCGGCGGCCTCCAAGCCACCAAGGATGGCGGCAAGACATGGATCACGGTTC
AGCCTGTGGAGGGAGCTTTTGTGGTCAACCTCGGGGACCATGGCCATTTTCTGAGCAATGGGAGGTTC
AAGAATGCTGACCACCAGGCTGTGGTGAACTCAAACTACAGCAGGCTCTCCATCGCCACGTTTCAGAA
CCCCGCGCCGGAGGCAATCGTGTATCCGCTGGCAGTAAGGGAGGGAGAGAAGCCGATTCTGGAGGAGC
CCATCACGTTTGCCGAGATGTACCGCAGGAAGATGAGCCGAGACCTCGAGCTCGCCAAGCTCAAGAAA
CTGGCCAAGACGGAGCAGAAGCAGCAGCCGGAGCTACTGGAGAAGACTAAGGACATCAACTTGGCCAA
GGCTACAGGCCTCGATGAGATCTTGGCTTAA

>GSMUA_Achr2P02770_001_(MaF3H1)_Protein

MAPGTAVLPTNEQTLRASFVRDEDERPKVAYNQFSLDIPVISLSGIDDDAAGGKRAEIRRKVVEACED
WGIFQVVDHGVDAGLISDMTRLAKEFFALSPEEKLLFDMSGGKKGGFIVSSHLQGEAVQDWREIVTYF
SYPIRARDYSRWPDKPEGWRSVVESYSEKLMGLACKLLEVLSEAMGLDKEALTEACIDMDQKVVVNYY
PKCPQPDLTLGLKRHTDPGTITLLLQDQVGGLQATKDGGKTWITVQPVEGAFVVNLGDHGHFLSNGRF
KNADHQAVVNSNYSRLSIATFQNPAPEAIVYPLAVREGEKPILEEPITFAEMYRRKMSRDLELAKLKK
LAKTEQKQQPELLEKTKDINLAKATGLDEILA

>GSMUA_Achr7T16780_001_(MaF3H2)_Genomic

GTCTAAGACCTTCTCATTCCCACACAGTTCAGATCGTTCAAGTGGGACAAGATGGCCCCCGGCACTGC
ATTGCTTCCCACGAACGAGGCGACCCTGAGGGCGAGCTTCGTGCGGGACGAGGACGAGCGACCCAAGG
TGGCCTATAACCAATTCAGCAGCGACATCCCGGTGATCTCGCTCGCCGGGGTTGACGACGAGGACGGG
GCCACCGGCGGGCGGAGGGCGGAGATCCGCCGCAAGATCGTGGAGGCCTGCGAGGACTGGGGCTTATT
CCAGGTGCTGGACCATGGAGTGGACGCCGGCATGATCTCCGAGATGACCCGGCTCGCCCGGGATTTCT
TCGCGCTACCCCCCGAGGAGAAGCTCCGGTTCGACATGTCCGGCGGAAAAAAGGGAGGCTTCATCGTG
TCCAGCCATCTCCAGGTAGAACATGTTTGACGAAGCCTTCTCACGAAGTTGGTGGATTCCATGGGCTA
AGAACTAATTCGATTGATGTCACGCAGGGTGAGGCAGTGCAGGACTGGCGAGAGATCGTGACTTACTT
CTCGTACCCGATCGGAGCTCGGGACTACTCGAGGTGGCCGGATAAGCCAGAAGGGTGGAGGTCGGTGG
TGGAGTCATACAGCGAGAAGCTGATGGGGTTGGCGTGCAAGCTGCTGGAGGTGTTGTCGGAGGCAATG
GGGCTCGACAAGGAAGCCCTCACCGATGCCTGCGTAGACATGGATCAGAAGGTGGTGGTCAACTACTA
CCCCAAGTGCCCCCAGCCCGACCTCACCCTCGGCCTCAAACGCCACACCGATCCCGGTACCATCACCC
TCCTCCTCCAAGACCAAGTCGGCGGACTCCAAGCCACCAAGGACGGCGGCAAGACGTGGATCACAGTT
CAGCCCGTGGAGGGAGCTTTCGTCGTCAACCTCGGGGACCATGGCCATGTGAGTATCAGCAAACGCCT
TCTTTCTGCGAATGGCATACAGATCTGACATGCTTCTCTCTGAGACGGGCGCAGTATCTGAGCAACGG
GAGGTTCAAGAACGCGGATCATCAGGCGGTGGTGAACTCGAGCTGCAGCAGGCTGTCGATCGCCACGT
TTCAGAACCCAGCGCCGGAGGCGATCGTGTATCCCCTGGCGATAAGGGAGGGAGACAAGCCGATCCTG

GACGAGCCCATCACGTTCAGCGAGATGTACCGTAGGAAGATGAGCCGAGATCTCGAGCTTGCCAACCT
CAAGAACCTGGCCAGGGCGGAGCATCAGCGGCTGGAGTTGCCGGAGAATGCCAACGACATCAATGTAG
CAAAGGCGAAATGCCTAAATGAGATCCTAGCTTAATATATATGCTATAGGTCTATGCCATCTCTCACG
TCCGAGCCACATAGTTTCCCGTCCGTGCGACGGTGAATGCTGTAATAAGATTTCGAGTACTCAGATAT
CTATAAAAAAATGTTCCGCAATTAAATTAAGATATCAATTACATCTAAAATATGATATAATATACTCA
CATA

>GSMUA_Achr7T16780_001_(MaF3H2)_Modified CDS

ATGGCCCCCGGCACTGCATTGCTTCCCACGAACGAGGCGACCCTGAGGGCGAGCTTCGTGCGGGACGA
GGACGAGCGACCCAAGGTGGCCTATAACCAATTCAGCAGCGACATCCCGGTGATCTCGCTCGCCGGGG
TTGACGACGAGGACGGGGCCACCGGCGGGCGGAGGGCGGAGATCCGCCGCAAGATCGTGGAGGCCTGC
GAGGACTGGGGCTTATTCCAGGTGCTGGACCATGGAGTGGACGCCGGCATGATCTCCGAGATGACCCG
GCTCGCCCGGGATTTCTTCGCGCTACCCCCCGAGGAGAAGCTCCGGTTCGACATGTCCGGCGGAAAAA
AGGGAGGCTTCATCGTGTCCAGCCATCTCCAGGGTGAGGCAGTGCAGGACTGGCGAGAGATCGTGACT
TACTTCTCGTACCCGATCGGAGCTCGGGACTACTCGAGGTGGCCGGATAAGCCAGAAGGGTGGAGGTC
GGTGGTGGAGTCATACAGCGAGAAGCTGATGGGGTTGGCGTGCAAGCTGCTGGAGGTGTTGTCGGAGG
CAATGGGGCTCGACAAGGAAGCCCTCACCGATGCCTGCGTAGACATGGATCAGAAGGTGGTGGTCAAC
TACTACCCCAAGTGCCCCCAGCCCGACCTCACCCTCGGCCTCAAACGCCACACCGATCCCGGTACCAT
CACCCTCCTCCTCCAAGACCAAGTCGGCGGACTCCAAGCCACCAAGGACGGCGGCAAGACGTGGATCA
CAGTTCAGCCCGTGGAGGGAGCTTTCGTCGTCAACCTCGGGGACCATGGCCATTATCTGAGCAACGGG
AGGTTCAAGAACGCGGATCATCAGGCGGTGGTGAACTCGAGCTGCAGCAGGCTGTCGATCGCCACGTT
TCAGAACCCAGCGCCGGAGGCGATCGTGTATCCCCTGGCGATAAGGGAGGGAGACAAGCCGATCCTGG
ACGAGCCCATCACGTTCAGCGAGATGTACCGTAGGAAGATGAGCCGAGATCTCGAGCTTGCCAACCTC
AAGAACCTGGCCAGGGCGGAGCATCAGCGGCTGGAGTTGCCGGAGAATGCCAACGACATCAATGTAGC
AAAGGCGAAATGCCTAAATGAGATCCTAGCTTAA

>GSMUA_Achr7P16780_001_(MaF3H2)_Protein

MAPGTALLPTNEATLRASFVRDEDERPKVAYNQFSSDIPVISLAGVDDEDGATGGRRAEIRRKIVEAC
EDWGLFQVLDHGVDAGMISEMTRLARDFFALPPEEKLRFDMSGGKKGGFIVSSHLQGEAVQDWREIVT
YFSYPIGARDYSRWPDKPEGWRSVVESYSEKLMGLACKLLEVLSEAMGLDKEALTDACVDMDQKVVVN
YYPKCPQPDLTLGLKRHTDPGTITLLLQDQVGGLQATKDGGKTWITVQPVEGAFVVNLGDHGHYLSNG
RFKNADHQAVVNSSCSRLSIATFQNPAPEAIVYPLAIREGDKPILDEPITFSEMYRRKMSRDLELANL
KNLARAEHQRLELPENANDINVAKAKCLNEILA

>GSMUA_Achr3T07030_001_(MaFLS1)_Genomic

GGATAAGTACTGCTACCAACAACAAAGGTTGAAACTAACCTCCTCTCCCGTATTTATAACACCCTCCG
CCGCTTCTCAAGTCCGACCACCACAACCACTTTCTTTGCTGCTTCCTCAGCAAAGACCGCAGGATGGA
GGTGGAGAGGGTGCAGGCCATCGCGTCCCTGAGCGTGGCCACCAACGACATACCGCCGGAGTTCGTGA
GGTCGGAGCACGAGCAGCCGGGTATCACCACGTACCGCGGCCCGGTCCCGGAGATCCCGGTGATCGAC
CTCGAAGACGGGGACGAAGGCCGGGTGACGCGCGCCATCGCGGAGGCCAGCCAGGAGTGGGGCATCTT
CCAGCTGGTGAACCACGGCATCCCCGGGGAGGTGATCCGGGCGCTGCAGCGCGTGGGCAGGGAGTTCT
TCGAGCTGCCACCGGAGGAGAAGGAGAAGTACGCGGCGGCGCCGGGGAGCCTCCAGGGCTACGGAACC
AAGCTGCAGAAGGACTTGGAAGGCAAGAAGGCGTGGGTGGACTTCCTCTTCCACAACATCTGGCCGCC
GACGCACGTCGACCACCGCGCATGGCCGGAGAATCCAGTGGATTACAGGTGCCCGCTCCTACCTCTCC
TTGCACGACACTACGCTTGGTCCAATATTGTCTAATATACTGTTCTCTCGCTTATCTCATGCAGCACT
TATATGTGTCACCAGCACTTGTATGGTCTCTCTCTTCTGCCTATACGACACTACCTTTCCTTCCTCTT
TCTAGGACTGTTTAATTTGATGTATTATAAATCTATAACCCAATTTAGATTGTATTCTTAAATCTTTT
TAGATCTCTAATAATACTTTCGATATTTTTGTTAGAAGTAAAATCTTTAATTATGAGGAAGTGTGAGG
ACTCTAATTTTTGCGTGTACTTTATAGATATAATATTTCAAGTAGACTCTCTCTTTAATATAATATTA
AAAATATTTTATCTAATTCAACAAAAGTCAATTATTTGTTTAAAGTTGATAGAAATAGATTAATCTTA

GCATAATTAATTATTACGAGAAAAATTCTTTTAAACACCAAATCAAAATCATTATCTCTATGTATATT
TCTTAGAATTTAGATCTAAGACACTTCCCTATCTCCTGTCTTCATGTTAAATTTGAATATATATATAT
ATATATATATATATATATATATATATATATATATATATATATATATATATATATATATAAAATCATTTCT
GTGGAATATTTGTCTTAAACACCATTCCTGTGGGGGGGAGTGACATGTCACGAGGAACTCAGGTTTCG
TCTTCTCCTCTCACGTTAAGTATTCATCCACTTCCATCACATTAAATTATGTGCAGGAGAGTTCATCT
AACCTAATTATTTCAGGAAGGCAAATGAGGAGTACGCCAAACATTTGGTGGGATTGGTGGAGAAGATG
TTGGTAAGCCTGTCCAAGGGACTGGGGCTGGAGGCCGACGTCCTCAAGCACGCAGTGGGAGGGGACGA
CTTGGAGTTCCTCCTCAAGATCAACTACTACCCGCCGTGCCCGAGACCCGACCTCGCCCTCGGCGTGG
TGGCTCACACCGACATGTCCGCCATCACCATCCTGATCCCCAACGACGTCCCCGGCCTCCAGGTCTTC
AAGGACGACCACTGGTTCGACGCCAAGTACGTCCCCGACGCCATCATCGTCCACATCGGGGACCAGAT
CGAGGTATGCAGACCTCAAACTATGCTTCCTCATCAATTTCATGTGCTGATGACGAGTGGTTGCAGAA
ACTGAGCAACGGCAGGTACAAGAGCGTGCTGCACCGGACGACGGTGAACAAGGAGAAGGCGAGGATGT
CGTGGCCTGTGTTCTGCTCCCCGCCGGGCGAGACGGTCATTGGGCCTCTGCCGCAGCTCGTCAGCGAC
GAACAGCCCGCTCAGTACAAGACGAAGAAGTACAAGGACTATGCTTTCTGCAAGCTGAACAAGCTTCC
GCAGTGAGGTCGCACCGGGTTCTTCTTGCATGACGGTATTGCTGTACTTTGCCTTATGTGTGGGTCCC
TTTTAAGCTCATATAGTGGTCTCTGTCCACTTTGAGTTGGCATAACTCCGTTGGAGCAGAAGGTGGAT
CCCGCTCCATCCTTCTTATCTAATAAACGAATATTTGGATGTAAGCCATCAGCGGGAAAATAAATCTT
TAGTTTTAGTACCTTTTCCGTAGTGTCCCTTTTAAGCTCATTATTTTTTTTTCCCATTGTAAATATAT
ATAAATATATATATAGTAATTTATAATGGAAAAAATAA

>GSMUA_Achr3T07030_001_(MaFLS1)_Modified CDS

ATGGAGGTGGAGAGGGTGCAGGCCATCGCGTCCCTGAGCGTGGCCACCAACGACATACCGCCGGAGTT
CGTGAGGTCGGAGCACGAGCAGCCGGGTATCACCACGTACCGCGGCCCGGTCCCGGAGATCCCGGTGA
TCGACCTCGAAGACGGGGACGAAGGCCGGGTGACGCGCGCCATCGCGGAGGCCAGCCAGGAGTGGGGC
ATCTTCCAGCTGGTGAACCACGGCATCCCCGGGGAGGTGATCCGGGCGCTGCAGCGCGTGGGCAGGGA
GTTCTTCGAGCTGCCACCGGAGGAGAAGGAGAAGTACGCGGCGGCGCCGGGGAGCCTCCAGGGCTACG
GAACCAAGCTGCAGAAGGACTTGGAAGGCAAGAAGGCGTGGGTGGACTTCCTCTTCCACAACATCTGG
CCGCCGACGCACGTCGACCACCGCGCATGGCCGGAGAATCCAGTGGATTACAGGAAGGCAAATGAGGA
GTACGCCAAACATTTGGTGGGATTGGTGGAGAAGATGTTGGTAAGCCTGTCCAAGGGACTGGGGCTGG
AGGCCGACGTCCTCAAGCACGCAGTGGGAGGGGACGACTTGGAGTTCCTCCTCAAGATCAACTACTAC
CCGCCGTGCCCGAGACCCGACCTCGCCCTCGGCGTGGTGGCTCACACCGACATGTCCGCCATCACCAT
CCTGATCCCCAACGACGTCCCCGGCCTCCAGGTCTTCAAGGACGACCACTGGTTCGACGCCAAGTACG
TCCCCGACGCCATCATCGTCCACATCGGGGACCAGATCGAGAAACTGAGCAACGGCAGGTACAAGAGC
GTGCTGCACCGGACGACGGTGAACAAGGAGAAGGCGAGGATGTCGTGGCCTGTGTTCTGCTCCCCGCC
GGGCGAGACGGTCATTGGGCCTCTGCCGCAGCTCGTCAGCGACGAACAGCCCGCTCAGTACAAGACGA
AGAAGTACAAGGACTATGCTTTCTGCAAGCTGAACAAGCTTCCGCAGTGA

>GSMUA_Achr3P07030_001_(MaFLS1)_Protein

MEVERVQAIASLSVATNDIPPEFVRSEHEQPGITTYRGPVPEIPVIDLEDGDEGRVTRAIAEASQEWG
IFQLVNHGIPGEVIRALQRVGREFFELPPEEKEKYAAAPGSLQGYGTKLQKDLEGKKAWVDFLFHNIW
PPTHVDHRAWPENPVDYRKANEEYAKHLVGLVEKMLVSLSKGLGLEADVLKHAVGGDDLEFLLKINYY
PPCPRPDLALGVVAHTDMSAITILIPNDVPGLQVFKDDHWFDAKYVPDAIIVHIGDQIEKLSNGRYKS
VLHRTTVNKEKARMSWPVFCSPPGETVIGPLPQLVSDEQPAQYKTKKYKDYAFCKLNKLPQ

>GSMUA_Achr8T23330_001_(MaFLS2)_Genomic

AGGCAACAGTACCTTTGTATGTCATCTCCTCTCCTTGTACCGGCTCAAGGATCCCTTACTGTGTCACC
AGGGAATGGCGCAAGTCAAGGTGCAAGCCATTGCCTCCATGTTACACTCCAAGGACACCATCCCTCCC
GAGTTCGTGCGCGTGGAGGACGAGCAGCCCGGCGCCACCACGTACCGCGGCCCCGCCCCCGCCGTCCC
GGTCATCGACCTCGCCGACACTGACCGGGTGGTGGACGCGATCGCCGACGCGAGCCGGGAGTGGGGGA
TCTTCCAGCTGGTGAACCACGGAATCCCGGCCGCTGTGATCGGGGAGCTGCAGCGCGTGGGGAGGGAG

TTCTTCGAGTTGCCGCAAGAGGAGAAGGAGTCTTACGCGGCGGATCCTCGTTCCGGGAGCATCGAGGG
TTACGGCACGCAGATCCAGAAGGATCCCAACGGCAAGAAGGCTTGGGGCGACTACTTGTTCCACAACG
TCTGGCCTCCTTCTCGCATCAACCATGGAATGTGGCCCCGTCAGCCTTCTTCGTATAGGTATGCTTAC
TCTTTCCTTCTCTTCCCAACTCACATCTACGTGTGCATGAGTCCTCTCTCTCTCTCTCTCTCTCTCTC
TCTCTCTCACACACACACACACACACACATCTACGTGTGAATGAGTCCTCTCTCTCTCTGTCTCTCTC
TCTCTTCACCTCATGATACAGGGAGGCTAACGAGGAATACACCAAGTACTTGCTGGGAATCTTGGACA
AGATACTGGACAGCCTTTCATTAGGGTTAGGGCTCGAAGGGAGTGCTCTGAAGAAGGCATTGGGAGGG
GAGGAGATGGACATGCTGCTCAAGATCAACTACTACCCGCCGTGCCCCCGGCCGGACCTGGCGCTCGG
GGTGGTGGCTCATAGCGACTTGTCGGCGGTCACTATACTCGTCCCCAGCGACGTCCCCGGCCTGCAAA
TCTCCAAGGATGACCGCTGGATCGACATCGATTACGTTCCCGGTGCCCTCATCATCCACATTGGCGAT
CAGATTGAGGTAGCTGCTACAATCCGGCGAGCAGACATTTCATGTTGTAACTTCTTGTTTCTTGCTGT
AGATTTTGAGCAATGGGAAGTACAAGAGCGTATTGCACAGGGCGACCGTGAACAAGGAGAAAGCAAGG
ATATCATGGCCGGTGTTCTGCTCGCCGCCGCCGGAGATGACGGTCGGCCCTCTGCCCCAGTTCGTGAG
TGATCAAAACCCTGCGAAATATAAGACGAAGAAGTACAAGGACTACCAGTACTGCAAGTTGAACAAGT
TGCCACAGTAAGACGATGATGAACCCAACATAGTCCGGCGAGCTTCAT

>GSMUA_Achr8T23330_001_(MaFLS2)_Modified CDS

ATGGCGCAAGTCAAGGTGCAAGCCATTGCCTCCATGTTACACTCCAAGGACACCATCCCTCCCGAGTT
CGTGCGCGTGGAGGACGAGCAGCCCGGCGCCACCACGTACCGCGGCCCCGCCCCCGCCGTCCCGGTCA
TCGACCTCGCCGACACTGACCGGGTGGTGGACGCGATCGCCGACGCGAGCCGGGAGTGGGGGATCTTC
CAGCTGGTGAACCACGGAATCCCGGCCGCTGTGATCGGGGAGCTGCAGCGCGTGGGGAGGGAGTTCTT
CGAGTTGCCGCAAGAGGAGAAGGAGTCTTACGCGGCGGATCCTCGTTCCGGGAGCATCGAGGGTTACG
GCACGCAGATCCAGAAGGATCCCAACGGCAAGAAGGCTTGGGGCGACTACTTGTTCCACAACGTCTGG
CCTCCTTCTCGCATCAACCATGGAATGTGGCCCCGTCAGCCTTCTTCGTATAGGGAGGCTAACGAGGA
ATACACCAAGTACTTGCTGGGAATCTTGGACAAGATACTGGACAGCCTTTCATTAGGGTTAGGGCTCG
AAGGGAGTGCTCTGAAGAAGGCATTGGGAGGGGAGGAGATGGACATGCTGCTCAAGATCAACTACTAC
CCGCCGTGCCCCCGGCCGGACCTGGCGCTCGGGGTGGTGGCTCATAGCGACTTGTCGGCGGTCACTAT
ACTCGTCCCCAGCGACGTCCCCGGCCTGCAAATCTCCAAGGATGACCGCTGGATCGACATCGATTACG
TTCCCGGTGCCCTCATCATCCACATTGGCGATCAGATTGAGATTTTGAGCAATGGGAAGTACAAGAGC
GTATTGCACAGGGCGACCGTGAACAAGGAGAAAGCAAGGATATCATGGCCGGTGTTCTGCTCGCCGCC
GCCGGAGATGACGGTCGGCCCTCTGCCCCAGTTCGTGAGTGATCAAAACCCTGCGAAATATAAGACGA
AGAAGTACAAGGACTACCAGTACTGCAAGTTGAACAAGTTGCCACAGTAA

>GSMUA_Achr8P23330_001_(MaFLS2)_Protein

MAQVKVQAIASMLHSKDTIPPEFVRVEDEQPGATTYRGPAPAVPVIDLADTDRVVDAIADASREWGIF
QLVNHGIPAAVIGELQRVGREFFELPQEEKESYAADPRSGSIEGYGTQIQKDPNGKKAWGDYLFHNVW
PPSRINHGMWPRQPSSYREANEEYTKYLLGILDKILDSLSLGLGLEGSALKKALGGEEMDMLLKINYY
PPCPRPDLALGVVAHSDLSAVTILVPSDVPGLQISKDDRWIDIDYVPGALIIHIGDQIEILSNGKYKS
VLHRATVNKEKARISWPVFCSPPPEMTVGPLPQFVSDQNPAKYKTKKYKDYQYCKLNKLPQ

>GSMUA_Achr10T25240_001_(MaFLS3)_Genomic

CCAGACCGGCAATCTCCCTTCTTATCTTAGCTTATCACAATCCACCAACTCGAGCGTAAGAAACGGCC
TCAGGCTAGTGCGTAGCACCCACGCAAGATGGAGGTGGAGAGGGTTCAAGTCATCGCGTCCGTCTGCG
CCGCCGACGGTGTTATGCCTCCGGAGTTCATACGGTCGGAGCACGAGCAGCCGGGCATCACCACCTAC
CGCGGCCCCGCGCCGGAGATCCCCGTCATCGACCTCGCGGGCGCCGACCGGGATCGGTTGACGATCGC
CGTCGCCGAGGCCAGCCGGGAGTGGGGAATCTTCCAACTGCTGAACCACGGGATCCCGAGGGAGGTGA
TCCGTGAGCTGCAGCGCGTCGGCAAGGAATTCTTCGAGTTGCCGCAGGAGGAGAAGGAGATGTACGCG
ATGGAATTCAAACCGGGGAGCTCGGAAGGATATGGGACTAAGCTGCAGAGGGAGTTGGAGGGCAAAAA
GGCCTGGGTTGACTTCTTCTTCCACTACGTATCGCCGCCGGCTCGCGTCAACCACGCCATCTGGCCCA

AGAACCCTTCTGATTACAGGTAAAGTGATCAGTTCTCTCTCTTTCTCACTGTTCTATGTGTATATCAA
GCTATCTTTACTGGAGGCAATTTCTACATTGAATCTAGGGAATTGTTTTCTTGTGTGTTGATTGAGAT
GATGACTCGTAGTAGGTGCCTCCGGCAGAAGGCAATGGTTATGTTTGTCGAGGATGTTGACTCGCCGG
AGATTTGGTAGAATTGGCCTGCGGAAGGAGACGATGTGGACGGGGCTTTGCCTTTAGAATATGGACCT
CGTGCTTCACGCATAGCATCAATTTTGTTTGTCTCATTCGTATCGTTGCATATGATACCACTTAGTAC
TACAGCCTTACTGAGTTAATTAGTTTATCAATTTCGTTTGGTCTAAACCACTGATCAAAATATTTAAG
TTGAAGTTTATAGTAGTATACTCATCAGACCCTTATAAATTAATTTTAATCTTATCCATTTTCGATGT
GAAACTAATATATTACAAGAGCCATGGAGAGGAGAGAGGACTACCGATCCAAACCTATGAATGACTGA
AGCTTGATGAGTCCATGATTCGATGATAGCAGCATCGCTAGAAAAATATTGAAACATAAGGAGAGATT
GAAATGAGGATTAAAAGAATAGTCCTACGATAACAAATATAAATATAGTAAGATAATATATATTGAGT
TGGATCCCCAAATCAAATAGCTTAAGTTTTTTATTGAGTTGATATCTGACTTGATTATCTAACCAAT
TTGATTCAATTTCATAGTTAATGGATTTCTAATATGATATCCGGAAAAAAAATTAGAGAGTAAAAAAA
AATATTCATATGTTGAAAAAGATTAAGATTAAGATTGTGTAAAAGATATCATAGATATGAGATGTCGG
AGGGATGAATCAGCCGCTGTTTTGACAGGAAAGCAAACGAGGAATATGCCAAACACCTGGTGGGACTG
GTGGACAAGATGCTGACGACCCTGTCGAGGGGACTGGGACTGGAGGAGCATGTCCTCAAGGGAGCACT
CGGTGGAGATGGACTGGAGCTACGCCTAAAGATGAACTACTACCCACCGTGTCCTCGGCCTGACCTGG
CTCTCGGCGTGGTGGCGCACACTGACATGTGTGCTATCACCTTCCTCGTCCCTAACCTTGTGCCGGGT
TTGCAGGTCTTCAAAGATGAGCACTGGATTGACGTCAACTTCATTCCTAATGCTGTCATCGTCCACAT
CGGTGATCAGATCGAGGTCACTCAGAAAGAACAAACTATATACATATACCATTCATCTTCTTTCTCAT
CTATTGGTGTCGTGTTATGTAGATTTTGAGCAATGGAACATACAAGAGCGTGCTGCACAGAACGACCG
TGAACAAAGAGAAGGTGAGGATGTCTTGGCCAGTGTTCTGCGCGCCTCCTGGTGAGATGGTAATCGGC
CCTCTGCAACAGCTCGTCGGCGATGAGAGCCCAGCCAAGTATAAGCCGAAGAAGTACAAAGACTACGC
CTACTGCAAGCTCAACAAGCTTCCGCAATAAATCTCCCTACAATTCGATCTTCCTATCTCTCTATGTT
TGAATTTAATTGGGTTGCCCAATTCCTCAGCATTGCCGAGAATAAACGAGTCTGGTTTCATACCACTC
ATGGCTGTAACTTGTGATACAGCTGCTTGAGTAAATGCGTGATGGCTGATTCTG

>GSMUA_Achr10T25240_001_(MaFLS3)_Modified CDS

ATGGAGGTGGAGAGGGTTCAAGTCATCGCGTCCGTCTGCGCCGCCGACGGTGTTATGCCTCCGGAGTT
CATACGGTCGGAGCACGAGCAGCCGGGCATCACCACCTACCGCGGCCCCGCGCCGGAGATCCCCGTCA
TCGACCTCGCGGGCGCCGACCGGGATCGGTTGACGATCGCCGTCGCCGAGGCCAGCCGGGAGTGGGGA
ATCTTCCAACTGCTGAACCACGGGATCCCGAGGGAGGTGATCCGTGAGCTGCAGCGCGTCGGCAAGGA
ATTCTTCGAGTTGCCGCAGGAGGAGAAGGAGATGTACGCGATGGAATTCAAACCGGGGAGCTCGGAAG
GATATGGGACTAAGCTGCAGAGGGAGTTGGAGGGCAAAAAGGCCTGGGTTGACTTCTTCTTCCACTAC
GTATCGCCGCCGGCTCGCGTCAACCACGCCATCTGGCCCAAGAACCCTTCTGATTACAGGAAAGCAAA
CGAGGAATATGCCAAACACCTGGTGGGACTGGTGGACAAGATGCTGACGACCCTGTCGAGGGGACTGG
GACTGGAGGAGCATGTCCTCAAGGGAGCACTCGGTGGAGATGGACTGGAGCTACGCCTAAAGATGAAC
TACTACCCACCGTGTCCTCGGCCTGACCTGGCTCTCGGCGTGGTGGCGCACACTGACATGTGTGCTAT
CACCTTCCTCGTCCCTAACCTTGTGCCGGGTTTGCAGGTCTTCAAAGATGAGCACTGGATTGACGTCA
ACTTCATTCCTAATGCTGTCATCGTCCACATCGGTGATCAGATCGAGATTTTGAGCAATGGAACATAC
AAGAGCGTGCTGCACAGAACGACCGTGAACAAAGAGAAGGTGAGGATGTCTTGGCCAGTGTTCTGCGC
GCCTCCTGGTGAGATGGTAATCGGCCCTCTGCAACAGCTCGTCGGCGATGAGAGCCCAGCCAAGTATA
AGCCGAAGAAGTACAAAGACTACGCCTACTGCAAGCTCAACAAGCTTCCGCAATAA

>GSMUA_Achr10P25240_001_(MaFLS3)_Protein

MEVERVQVIASVCAADGVMPPEFIRSEHEQPGITTYRGPAPEIPVIDLAGADRDRLTIAVAEASREWG
IFQLLNHGIPREVIRELQRVGKEFFELPQEEKEMYAMEFKPGSSEGYGTKLQRELEGKKAWVDFFFHY
VSPPARVNHAIWPKNPSDYRKANEEYAKHLVGLVDKMLTTLSRGLGLEEHVLKGALGGDGLELRLKMN
YYPPCPRPDLALGVVAHTDMCAITFLVPNLVPGLQVFKDEHWIDVNFIPNAVIVHIGDQIEILSNGTY
KSVLHRTTVNKEKVRMSWPVFCAPPGEMVIGPLQQLVGDESPAKYKPKKYKDYAYCKLNKLPQ

>GSMUA_Achr10T25250_001_(MaFLS4)_Genomic

AATCCATCAACGTGAACAAAAGAACCAGGTTCTTGCTAGTAGCAGCTCCGACCTTGACCAACACAAGA
TGGAGATGGAGAGGGTTCAAGCCATCGCGTCCGTCTGTGCAGCCAACGGCGCCATGCCGCCAGAGTTC
ATACGGTCGGAGCACGAGCAGCCGGGCATCACCACCTACCGCGGCCCCGCCCCGGAGATCCCGGTCAT
CGACCTCGCGGGCGACAACCAGGACCAGTTGACGATCGCCGTGGCCGAGGCCAGCCGGGAGTGGGGAA
TCTTCCAACTGCTGAACCACGGGATTCCGGGGGAGGTGATACGTGAGCTGCAGCGCGTCGGCATGGAA
TTCTTCGAGCTACCGCAGGAGGAGAAGGAGAAGTACGCGATGGTGCCCGGATCGGGGAGCTTGGAAGG
ATATGGCACCAAGCTGCAGAAGGAGTTGGAGGGCAAGAAGGCCTGGGTTGATTTCTTCTTCCACTACG
TATCGCCGCCGTCTCGCGTCAACCACGCCATCTGGCCCAAGAAACCTGCCGATTATAGGTAAAGTAAT
TATGTATACATATATATGTATATAGTGAGTTTATATATGGAATTATTCTCTTTTATGTGTTGTTTGAG
GTGGTGACTAATTGGAGTTGGTGATCAGATTGAGGATTAATTTTATGTTAGTTGAGGATGATGCTTCG
TGTAGACTTTCAAGGAAATTGGCGTAGGGAAGGAGACAATGTGCATGGTGTATTTTATATGGGCAAAG
CAAGGTCATTGCTTAAGAATATGAGCCTCGCATTCCACGGCGAGCCATATTTTAAATGCCACTTCCAC
ATCACCGCCATGTGCTTCATCCAGTCAAACCTCAGTATGTCTTGAATTATCTGACTCATCTAGGTAAC
CTGTTTTGACCTTTGCGGTTTTGACAGGCAAGTAAACGAGCACTACGGCAAACACCTGGCGTGTCTTG
TGGACAGGATGCTGATGGCGCTGTCGAGGGGACTGGGACTGGGAGATCATGTCCTCAAGGAAGCACTT
GGCGGAGATGGACTGGAGCAACTCCTAAAGATCAACTACTACCCGCCGTGTCCTCGGCCTGACCTGGC
TCTCGGGGTGGTGGCTCACACTGACATGTCCGCTATCACCATTCTCGTCCCCAACCATGTCCCTGGTT
TGCAGGTCTTCAACGATGAGCACTGGATCGACGTCAACTACGTTCCTGATGCTGTCATCGTCCACATC
GGTGATCAGATCGAGGTCACCAAAATAACTAAAAGTATACATATCTCATTCATTCTTCTTCCTACTCA
TCTATTGGCGTTGTGTTACGTAGATTTTGAGCAATGGGATATACAAGAGCGCGTTGCACAGAACAACC
GTGAACAAAGAGAAGGTCAGGATGTCGTGGCCAGTCTTCTGCTCTCCTCCTGGTGAGATGGTAATTGG
CCCTCTGCAACAGCTCGTCGGTGATGAGAGCCCTCCCAAGTACAAGGCCAAGAAGTACAAAGACTACG
CCTACTGCAAGCTCAACAAGCTTCCACAGTAAACGTTGTTACGTTACATGCTTTTCCCGTTCCTCGTC
ATCGTT

>GSMUA_Achr10T25250_001_(MaFLS4)_Modified CDS

ATGGAGATGGAGAGGGTTCAAGCCATCGCGTCCGTCTGTGCAGCCAACGGCGCCATGCCGCCAGAGTT
CATACGGTCGGAGCACGAGCAGCCGGGCATCACCACCTACCGCGGCCCCGCCCCGGAGATCCCGGTCA
TCGACCTCGCGGGCGACAACCAGGACCAGTTGACGATCGCCGTGGCCGAGGCCAGCCGGGAGTGGGGA
ATCTTCCAACTGCTGAACCACGGGATTCCGGGGGAGGTGATACGTGAGCTGCAGCGCGTCGGCATGGA
ATTCTTCGAGCTACCGCAGGAGGAGAAGGAGAAGTACGCGATGGTGCCCGGATCGGGGAGCTTGGAAG
GATATGGCACCAAGCTGCAGAAGGAGTTGGAGGGCAAGAAGGCCTGGGTTGATTTCTTCTTCCACTAC
GTATCGCCGCCGTCTCGCGTCAACCACGCCATCTGGCCCAAGAAACCTGCCGATTATAGGCAAGTAAA
CGAGCACTACGGCAAACACCTGGCGTGTCTTGTGGACAGGATGCTGATGGCGCTGTCGAGGGGACTGG
GACTGGGAGATCATGTCCTCAAGGAAGCACTTGGCGGAGATGGACTGGAGCAACTCCTAAAGATCAAC
TACTACCCGCCGTGTCCTCGGCCTGACCTGGCTCTCGGGGTGGTGGCTCACACTGACATGTCCGCTAT
CACCATTCTCGTCCCCAACCATGTCCCTGGTTTGCAGGTCTTCAACGATGAGCACTGGATCGACGTCA
ACTACGTTCCTGATGCTGTCATCGTCCACATCGGTGATCAGATCGAGATTTTGAGCAATGGGATATAC
AAGAGCGCGTTGCACAGAACAACCGTGAACAAAGAGAAGGTCAGGATGTCGTGGCCAGTCTTCTGCTC
TCCTCCTGGTGAGATGGTAATTGGCCCTCTGCAACAGCTCGTCGGTGATGAGAGCCCTCCCAAGTACA
AGGCCAAGAAGTACAAAGACTACGCCTACTGCAAGCTCAACAAGCTTCCACAGTAA

>GSMUA_Achr10P25250_001_(MaFLS4)_Protein

MEMERVQAIASVCAANGAMPPEFIRSEHEQPGITTYRGPAPEIPVIDLAGDNQDQLTIAVAEASREWG
IFQLLNHGIPGEVIRELQRVGMEFFELPQEEKEKYAMVPGSGSLEGYGTKLQKELEGKKAWVDFFFHY
VSPPSRVNHAIWPKKPADYRQVNEHYGKHLACLVDRMLMALSRGLGLGDHVLKEALGGDGLEQLLKIN
YYPPCPRPDLALGVVAHTDMSAITILVPNHVPGLQVFNDEHWIDVNYVPDAVIVHIGDQIEILSNGIY
KSALHRTTVNKEKVRMSWPVFCSPPGEMVIGPLQQLVGDESPPKYKAKKYKDYAYCKLNKLPQ

>GSMUA_Achr3T30350_001_(MaF3'H)_Genomic

AGAAAGGAATGGAAGGTAACGCAGGTTACCTCACGGTGAGCTGACTGTTGACTGCACCCCCCTTACAT
GGCCAATGTCCTTTTAAATAAGAGAAGCGGCTCCGAGTGCAAGAGAGAAGCATACTGCAGCATGGATC
TTATTCTACTTGGCCTCTTCACCCTCGGCCTCTCATCCCTAATCCTCTTCTTCCTCCACCTCAAGCAG
TCGAGGAGGAAGCCCCTGCCGCCCGGTCCCCGCGGCTGGCCGATCCTCGGCAACCTCCCCCAGCTGGG
GCCGAAGCCTCACCGCACGCTGCACGCGCTGGCCAAGGTCCACGGCCCGCTCTTCCGCCTCCGCTTCG
GCTCCGTGGACGTCGTCGTCGCCGCCTCCGCGGCCGTCGCTTCCCAGCTGCTCCGCGCGCACGACGCC
ATCTTCTGTGACCGGCCGCCCAACTCCGGCGCCGAGCACGTCGCCTACAACTACCAGGACCTCGTGTT
CGCGCCATACGGCCCCCGCTGGCGTATGCTCCGCAAGCTCTGTTCCGTCCACCTCTTCAGCGCCAAGG
CGCTCGACGACCTCCGGTGGGTACGGCAGGGCGAGGTGGGGCTCCTGGTGCACGCGCTCCGCGCTTGC
GGCGATGCGCCGGTCAACCTGGGCTACGCCGTGAACGTCTGCGCCACCAACGCGCTGGCCAGGGCCAC
CATGGGGCGGCGGGTGTTCGAGGAGGACGGTAGCCGGGAGGGCGCTGGGGAGTTCAAGGAGATGGTGG
TGGAGCTGATGAGGCTGGCGGGGGAGTTCAACGTCGGCGACTTCGTCCCGTGGCTGAACTGGCTCGAC
CCCCAGGGGGTGGTAGCCAGGATGAAGAGGCTGCACCGCAGGTACGACGAGTTCCTCGACGGGATCAT
CGCGGAGCACCGCCGCCGCGCGGAGGCTGCCGAGGGGGAGGACGACCCGAGCGGCCGCGGAAGGGACC
TGTTGAGCGTGTTGATAGCGTTGACCGAGAGGCCGGACGGTGAAGGCGATGGCGGAAAGCTCACCGAC
ACCAACATTAAGGCCCTACTATTGGTACGTATTACGATACTGCCACTCCTTCACTATTCACAGAGGGG
ACGGACACGTCATAGTGTTAATGAAGCTTCCATGACAGAACCTGTTCACAGCGGGGACGGACACGTCA
TCGAGCACGGTGGAGTGGGCGTTGGCGGAGCTCATCCGGCACCCGGACGTCCTCAAGCAAGCCCAGCG
GGAGCTCGACTCGGTGGTCGGCCGATCCCGGCTCGTCACCGAGTCCGACCTCCCCAACCTGCGCTTCC
TGCAAGCCGTCATCAAGGAGACGTTCCGGCTCCACCCGTCCACGCCGCTGTCCCTCCCGCGCGTGGCG
TCGGAGGCGTGCGAGGTGGGCGGCTACCAGATTCCCCGGGGCGCGACGCTGCTCGTCAACATCTGGGC
CATCACCCACGACCCGGCGTCGTGGCCGAACCCGCTCGAGTTCAACCCGGCCAGGTTCCTCCCCGGCG
GCGGGCACGAGAGCGTCGACCTCAGGGGGCAAGACTTCGAGCTCATCCCCTTCGGAGCCGGGCGGAGG
ATCTGCGCCGGGATGAGCCTGGGGATCAGGATGGTGCAGTTCATGACGGCCACCCTGGTGCACGCCTT
TGACTGGAGTCTGCCGGAGGGGCAGAAGCCGGAGAAGCTCGACATGGAGGAAGCCTATGGGCTGACGC
TGCAGCGGGCCGTGCCTTTGATGGTGCATCCTCGTCCCAGGCTGACCTCTGCGGCGTACGAGGCCGGT
TGTTGAATCGTACCACCGAATCCGCCTTGCGTTCAATAAGAGATTCGCGAAGTGACATCAGCGAAGCA
TCAACAATGCTTGCCCGCGTGTGTGTTGTTTAGTAGGGTGCATGGGCTCACCAGAGCTTCCATGGGAA
CGTTATATGATGTCGATGATATGTTTAATGAGATTTCCAATTGCAATGTTAGGACTCTTTTTCCCCAT
TTCACTCCTACCATTTTAGATATTAAAGGATGGTAGGAGTGCAACGAGGTTCACATGATACTCGCTCT
TGGAAGAGGC

>GSMUA_Achr3T30350_001_(MaF3'H)_Modified CDS

ATGGATCTTATTCTACTTGGCCTCTTCACCCTCGGCCTCTCATCCCTAATCCTCTTCTTCCTCCACCT
CAAGCAGTCGAGGAGGAAGCCCCTGCCGCCCGGTCCCCGCGGCTGGCCGATCCTCGGCAACCTCCCCC
AGCTGGGGCCGAAGCCTCACCGCACGCTGCACGCGCTGGCCAAGGTCCACGGCCCGCTCTTCCGCCTC
CGCTTCGGCTCCGTGGACGTCGTCGTCGCCGCCTCCGCGGCCGTCGCTTCCCAGCTGCTCCGCGCGCA
CGACGCCATCTTCTGTGACCGGCCGCCCAACTCCGGCGCCGAGCACGTCGCCTACAACTACCAGGACC
TCGTGTTCGCGCCATACGGCCCCCGCTGGCGTATGCTCCGCAAGCTCTGTTCCGTCCACCTCTTCAGC
GCCAAGGCGCTCGACGACCTCCGGTGGGTACGGCAGGGCGAGGTGGGGCTCCTGGTGCACGCGCTCCG
CGCTTGCGGCGATGCGCCGGTCAACCTGGGCTACGCCGTGAACGTCTGCGCCACCAACGCGCTGGCCA
GGGCCACCATGGGGCGGCGGGTGTTCGAGGAGGACGGTAGCCGGGAGGGCGCTGGGGAGTTCAAGGAG
ATGGTGGTGGAGCTGATGAGGCTGGCGGGGGAGTTCAACGTCGGCGACTTCGTCCCGTGGCTGAACTG
GCTCGACCCCCAGGGGGTGGTAGCCAGGATGAAGAGGCTGCACCGCAGGTACGACGAGTTCCTCGACG
GGATCATCGCGGAGCACCGCCGCCGCGCGGAGGCTGCCGAGGGGGAGGACGACCCGAGCGGCCGCGGA

AGGGACCTGTTGAGCGTGTTGATAGCGTTGACCGAGAGGCCGGACGGTGAAGGCGATGGCGGAAAGCT
CACCGACACCAACATTAAGGCCCTACTATTGAACCTGTTCACAGCGGGGACGGACACGTCATCGAGCA
CGGTGGAGTGGGCGTTGGCGGAGCTCATCCGGCACCCGGACGTCCTCAAGCAAGCCCAGCGGGAGCTC
GACTCGGTGGTCGGCCGATCCCGGCTCGTCACCGAGTCCGACCTCCCCAACCTGCGCTTCCTGCAAGC
CGTCATCAAGGAGACGTTCCGGCTCCACCCGTCCACGCCGCTGTCCCTCCCGCGCGTGGCGTCGGAGG
CGTGCGAGGTGGGCGGCTACCAGATTCCCCGGGGCGCGACGCTGCTCGTCAACATCTGGGCCATCACC
CACGACCCGGCGTCGTGGCCGAACCCGCTCGAGTTCAACCCGGCCAGGTTCCTCCCCGGCGGCGGGCA
CGAGAGCGTCGACCTCAGGGGGCAAGACTTCGAGCTCATCCCCTTCGGAGCCGGGCGGAGGATCTGCG
CCGGGATGAGCCTGGGGATCAGGATGGTGCAGTTCATGACGGCCACCCTGGTGCACGCCTTTGACTGG
AGTCTGCCGGAGGGGCAGAAGCCGGAGAAGCTCGACATGGAGGAAGCCTATGGGCTGACGCTGCAGCG
GGCCGTGCCTTTGATGGTGCATCCTCGTCCCAGGCTGACCTCTGCGGCGTACGAGGCCGGTTGTTGA

>GSMUA_Achr3P30350_001_(MaF3'H)_Protein

MDLILLGLFTLGLSSLILFFLHLKQSRRKPLPPGPRGWPILGNLPQLGPKPHRTLHALAKVHGPLFRL
RFGSVDVVVAASAAVASQLLRAHDAIFCDRPPNSGAEHVAYNYQDLVFAPYGPRWRMLRKLCSVHLFS
AKALDDLRWVRQGEVGLLVHALRACGDAPVNLGYAVNVCATNALARATMGRRVFEEDGSREGAGEFKE
MVVELMRLAGEFNVGDFVPWLNWLDPQGVVARMKRLHRRYDEFLDGIIAEHRRRAEAAEGEDDPSGRG
RDLLSVLIALTERPDGEGDGGKLTDTNIKALLLNLFTAGTDTSSSTVEWALAELIRHPDVLKQAQREL
DSVVGRSRLVTESDLPNLRFLQAVIKETFRLHPSTPLSLPRVASEACEVGGYQIPRGATLLVNIWAIT
HDPASWPNPLEFNPARFLPGGGHESVDLRGQDFELIPFGAGRRICAGMSLGIRMVQFMTATLVHAFDW
SLPEGQKPEKLDMEEAYGLTLQRAVPLMVHPRPRLTSAAYEAGC

>GSMUA_Achr2T00210_001_(MaF3'5'H1)_Genomic

CACCATCGTCTACGTACCACCACAATCTCCATGGCTCTCGATATTGTTCTCGTCGCCGGCATCCTCTT
GAGCGTTCTTGTCCACCTTCTCCTCCGCCGCAGACTCCATTCCATCCGCCGACTCCCGCTCCCTCCTG
GTCCAGCGGGCATCCCAATCCTCGGCTCGCTGCCGCAAATCGGTCCCATGCCCCACGCCTCCCTCGCT
AACCTCGCCGCGCGTTACGGCCCCATCATGTACCTCAGGATGGGCACTACGGGGGTCGTAGTTGCGTG
CTCCGCTGGCGCCGCCCGCTCCTTTCTCAAGGCGCTTGACCTTCAGTTCGCCAACCGCCCCTCGCCCA
TCAGCGGGAAGGACGTCACCTACGACGGCCAGGACTTCGTGTTCGCCAACTACGGGCCTCGGTGGAAC
CTCCTCCGCAAGCTCACTAACCTCCACTTTCTCGGCAGCAAGGCGCTGACTATGTGGGCGCCGGTTCG
CCGCGACGAAATCGGTCGCATGCTCCGCGCCATGCTCGAGTCGAGCCGGAACTCGCGGCCGGTGATGG
TGTCGGAGGCAATGGTGTGCGCCAGCGCCAACATCATCGGGCAGGTAATGCTCTCGCGGCGGGTGTTT
GAATCGCAGGGAGAGGAATCAAAGCAGTTCAAGGACGCCATCACGGAACTGCTGGCTTGGTCGGGGAA
GTTCAGCATCGGCGACTTTGTGCCAGCGATCGCGTGGATGGACCTTCAGGGAGTGCAGCGGAAGCTGC
GCCGGGTGCATGTGAAGTTAGACACTCTGATCACGGCGCTCATGGCGGAGCACGAGGCGACGGCGCAC
GAGCGCGAGGGGAGGCCGGACGTGCTGGATCTTGTGATGGCCAATAGGGTTGACGCTGATGGGGTGTC
GCTTTCTGATGTCAACATAAAGGGCTTTATCTCTGTGAGCTCTCTTTCCCTCTCTCTCCTTCCCCCAC
TTCTTCGCTACGGACAGCCAATTAGCGACGTCCGCGAATTCTATTGACCTCCATCTCCAAAACGGTCC
TCCTGTTTTCTCTTAGTATCAGTCATCGTTCTCATTTGACCAAGAATGATGGTCGATACCCTCTCAGA
ACAGGTGTTGGATCCTGCAACCAAACCAGCACTAAATATACATATAGATAGAGAGCTACAAAACGTAA
ACAGATGAGTTCCGCATGAATTCCTACGTTGTCTGCTCGATTATGGCACCATTATTCGAGTCAGATAA
GATTCTCCCTGGAGGGGAAAAGATATTACCTAATGCGCACTTTATTGGAGGCTCTCCTGTTTAATAAG
TTCTTCATGCATGTTTGCTACCACACAAATACCTCAGCAACTTCTTCTCCTGGCAGGATATGTTTATT
GCCGGAACTGATACATCATCCATCATAATCGAATGGGCGCTCGCAGAAATGCTAAGGAACCCAACCAT
CCTGCAGCGAGCTCAAGATGAGATGGACCAAGTAATCGGGAAGAACCGTAGGCTTGCAGAGTCTGACA
TACCAAGCCTTCCCTACTTACGAGCCATCTGCAAAGAGGCATTACGATTGCACCCTTCCACGCCGCTC
AGCCTCCCACACTACACCTTCGAAGCATGCGAGGTGGACGGCTACCACATCCCCCCAAACACACGGCT
CATAGTCAACGTATGGGCCATTGGGAGAGACCCCGACGTGTGGGAGCATCCCCTGGAGTTCAACCCGG
AGAGGTTCTTAAGCGGTAGAACCGCCAAGATTGAGCCACTGGGGAACGACTTCGAGCTGATACCTTTC
GGCGCCGGGAGAAGGATCTGCGTGGGAATGCATGCAGGTCTCATCATGCTACAATATGGGCTCGGGTC
TTTGTTGCACTCATTTCATTGGAAGCTTGCCGATGACGTCGAGGAGCTCGACATGAAGGAGAAATTTG

GCGCCGCGCTTCCGAAGGCAGTGCCTCTCGAGGCTGTAGTTAGCCCACGCCTCCTCGAAAGTGCCTAT
ATGTGATCTGTGTAGGTTAAACATGTATAATATGCATTAGGTATATTATACACAACCTAAAAAATAAG

>GSMUA_Achr2T00210_001_(MaF3'5'H1)_Modified CDS

ATGGCTCTCGATATTGTTCTCGTCGCCGGCATCCTCTTGAGCGTTCTTGTCCACCTTCTCCTCCGCCG
CAGACTCCATTCCATCCGCCGACTCCCGCTCCCTCCTGGTCCAGCGGGCATCCCAATCCTCGGCTCGC
TGCCGCAAATCGGTCCCATGCCCCACGCCTCCCTCGCTAACCTCGCCGCGCGTTACGGCCCCATCATG
TACCTCAGGATGGGCACTACGGGGGTCGTAGTTGCGTGCTCCGCTGGCGCCGCCCGCTCCTTTCTCAA
GGCGCTTGACCTTCAGTTCGCCAACCGCCCCTCGCCCATCAGCGGGAAGGACGTCACCTACGACGGCC
AGGACTTCGTGTTCGCCAACTACGGGCCTCGGTGGAACCTCCTCCGCAAGCTCACTAACCTCCACTTT
CTCGGCAGCAAGGCGCTGACTATGTGGGCGCCGGTTCGCCGCGACGAAATCGGTCGCATGCTCCGCGC
CATGCTCGAGTCGAGCCGGAACTCGCGGCCGGTGATGGTGTCGGAGGCAATGGTGTGCGCCAGCGCCA
ACATCATCGGGCAGGTAATGCTCTCGCGGCGGGTGTTTGAATCGCAGGGAGAGGAATCAAAGCAGTTC
AAGGACGCCATCACGGAACTGCTGGCTTGGTCGGGGAAGTTCAGCATCGGCGACTTTGTGCCAGCGAT
CGCGTGGATGGACCTTCAGGGAGTGCAGCGGAAGCTGCGCCGGGTGCATGTGAAGTTAGACACTCTGA
TCACGGCGCTCATGGCGGAGCACGAGGCGACGGCGCACGAGCGCGAGGGGAGGCCGGACGTGCTGGAT
CTTGTGATGGCCAATAGGGTTGACGCTGATGGGGTGTCGCTTTCTGATGTCAACATAAAGGGCTTTAT
CTCTGATATGTTTATTGCCGGAACTGATACATCATCCATCATAATCGAATGGGCGCTCGCAGAAATGC
TAAGGAACCCAACCATCCTGCAGCGAGCTCAAGATGAGATGGACCAAGTAATCGGGAAGAACCGTAGG
CTTGCAGAGTCTGACATACCAAGCCTTCCCTACTTACGAGCCATCTGCAAAGAGGCATTACGATTGCA
CCCTTCCACGCCGCTCAGCCTCCCACACTACACCTTCGAAGCATGCGAGGTGGACGGCTACCACATCC
CCCCAAACACACGGCTCATAGTCAACGTATGGGCCATTGGGAGAGACCCCGACGTGTGGGAGCATCCC
CTGGAGTTCAACCCGGAGAGGTTCTTAAGCGGTAGAACCGCCAAGATTGAGCCACTGGGGAACGACTT
CGAGCTGATACCTTTCGGCGCCGGGAGAAGGATCTGCGTGGGAATGCATGCAGGTCTCATCATGCTAC
AATATGGGCTCGGGTCTTTGTTGCACTCATTTCATTGGAAGCTTGCCGATGACGTCGAGGAGCTCGAC
ATGAAGGAGAAATTTGGCGCCGCGCTTCCGAAGGCAGTGCCTCTCGAGGCTGTAGTTAGCCCACGCCT
CCTCGAAAGTGCCTATATGTGA

>GSMUA_Achr2P00210_001_(MaF3'5'H1)_Protein

MALDIVLVAGILLSVLVHLLLRRRLHSIRRLPLPPGPAGIPILGSLPQIGPMPHASLANLAARYGPIM
YLRMGTTGVVVACSAGAARSFLKALDLQFANRPSPISGKDVTYDGQDFVFANYGPRWNLLRKLTNLHF
LGSKALTMWAPVRRDEIGRMLRAMLESSRNSRPVMVSEAMVCASANIIGQVMLSRRVFESQGEESKQF
KDAITELLAWSGKFSIGDFVPAIAWMDLQGVQRKLRRVHVKLDTLITALMAEHEATAHEREGRPDVLD
LVMANRVDADGVSLSDVNIKGFISDMFIAGTDTSSIIIEWALAEMLRNPTILQRAQDEMDQVIGKNRR
LAESDIPSLPYLRAICKEALRLHPSTPLSLPHYTFEACEVDGYHIPPNTRLIVNVWAIGRDPDVWEHP
LEFNPERFLSGRTAKIEPLGNDFELIPFGAGRRICVGMHAGLIMLQYGLGSLLHSFHWKLADDVEELD
MKEKFGAALPKAVPLEAVVSPRLLESAYM

>GSMUA_Achr8T05810_001_(MaF3'5'H2)_Genomic

ACCGAGTCTCCACTATGGTCCGGAGTCGATGTGTAGTGACTGTAATCATTCAATGGTGTGGTGTGTGC
TGTGTACATTATGATGTCCCTGTATATCATATCGTTTGCTTGTCTTTATATATGTACATGTATATGTA
TGTATATGTATATATATATGTATGTATATATATGTAAATATATATATATACATATGTATGTATATATG
TATATATATATATGTATGTATATATGTGTGTGTATATATATCGACAGCTCGGCACTAAGCATCACATA
ACATAATCAATTTGCTTGTGGCTGTTTGCTGAGCACCACCATGCAAATCGACCCCTTCATCGCGGCAA
CCGCCGCGCTCTGCCTCGTCATCCACCACCTCCTCCGCCGCTTCCTTCACAGGTCGCCCTCTCGCCTC
CCCCTCCCGCCCGGCCCCCGAGGCTACCCTGTCCTCGGCGCTTTGCCCCTCGTCGGCCTCCAGGCGCA
CACCGGCCTAGCCCGCCTGGCCCAGCGCTACGGCCCCATTATGTACCTCAAGATGGGAAGCTGCGGCT
GCGTCGTGGCATCTGACGCCGGCGCTGCCCGCGCCTTCCTCAAGGCCCACGACGCGCAGTTCGCCAAC
CGCCCGAACGTCATCAGCGCCATGGACGTCACCTATCACCGCCAGAACATGGTGTTCGCCGACTACGG

CCCCAAGTGGAAGCTCCTCCGCAAGCTCTGCAGCCTCCACCTCCTGGGCGGCAAGGCGCTTGCCGACT
GGGCCCCGGTGCGCCGCGCCGAGTTCGGCCACATGGTCGTGTCCATGCACCGAGCGGCCGCGGAGGGC
CGCCCTGTGGTGCTGCCGGAGATGCTGGTGTGCGCCCTGGCCAACATCATTGGCCAGATAGTGGTGAG
CAAGCGGGTGTTCGACGTCCAGGGCATCGAGTCGAACCATTACAAGGACATGATCGTGGAGCTGCTGA
CTGGGGGCGGGCTGTTCAACATCGGCGACTTCGTGCCGGCTATCGCGTGGATGGACCTGCAGCGGGTA
CAGGCGAAGATGCGGAGGGTGCATGTTCGGTTCGACGCCATGGTGACGAAGCTGTTGGAGGAGCACGA
GACCACCAAGGAGGAGCGCAGAGGGAGGCCGGACTTCATCGACACTATCATGGCCAACAGGGAGGGAG
AGGACGGCGAGACCATCACGGACGTCAACGTCAAGGCTATCATCTTTGTGAGTTCTCCTTGAGCTTTC
CACTCCCAAGATTAGGTGTCTGATTAAGGTGCTCGACCAAATGCCCATCGAATAACTTACAAATCTAC
TCCACTCCAGCATGCGTCACCATCCATCTTTAGATTAGGTTCCGAAGGCTATTCTTGAATTGGCCGGT
GGGGCGAGGAGGACACCATCAGCACTCCTCGAGATTCTAACCTTCGTTTGATCAAACCTTGGTTGCTG
ATCACGTCTGCACTCTGCATGCAGGACCTGTTCACAGCCGGCACCGACACGTCGGCGGTCATCGTGGA
GTGGGCGCTGGCGGAGATGCTGAAGAACCCGAGCATCCTCCGCCGCCTGCAATCCGAGATCGACTACG
TGGTGGGCCGAGGCCGCATGGTCCAGGAATCCGACCTGCCCAAGCTCCCCTACCTGCACGCGGTGTGC
AAGGAAGCGCTGCGCCTGCACCCGTCCACACCTCTCGGCCTCCCCCACTTCTCCTTCGAGGAGTGCGA
CGTGAACGGCTACTACATCCCCGGCAACACCCGCCTCCTCGTCAACATCTGGGCCATCGGGCGCGACC
CCGCCGCGTGGGACGACCCCCTCGCGTTCGACCCCGACCGCTTCGTCTCCGGCAAGGCGGCCAAGATC
GACCCGCAGGGCAACGACTTCGAGCTGATACCGTTCGGGGCCGGGCGGCGGATCTGCGCGGGGAAGCT
CGTGGGCATGGTGTTCGTGCAGTACATGCTGGCGACGTTGGTGCACTCGTTCGACTGGAAGCTGCCGG
AGGGGGAGGAGCTCGACATGGAGGAGAAGTTTGGGTTGGCGCTCCCCAAGGCTGTGCCTGTCAAGGCG
CTTCTGAGCCCACGCTTGGCGACTGAAGCATACATTTGATCCGCCCACGCACTCATGCATGGCTTTCC
CTTCTGTACTGTTTTGCTTCTTGTTTGGATTTGTCTCTTTGGTTTTGATGCATGTATATGTTCGACTA
CAAAACATCTTCATGGAAAATGTTAGATAAACGTTACCATATAGAATCCACGGCATTCTCAGAACACA
TGCATCGGTGGCATAAAGAGGATCCAATAGCCTGTTTGTTCTCAGATAGGAACACAACATCACCCTTT
GCACGGCATTCTCATAAACATATCAACAGCTTCAATTCGCAAGAAACA

>GSMUA_Achr8T05810_001_(MaF3’5’H2)_Modified CDS

ATGCAAATCGACCCCTTCATCGCGGCAACCGCCGCGCTCTGCCTCGTCATCCACCACCTCCTCCGCCG
CTTCCTTCACAGGTCGCCCTCTCGCCTCCCCCTCCCGCCCGGCCCCCGAGGCTACCCTGTCCTCGGCG
CTTTGCCCCTCGTCGGCCTCCAGGCGCACACCGGCCTAGCCCGCCTGGCCCAGCGCTACGGCCCCATT
ATGTACCTCAAGATGGGAAGCTGCGGCTGCGTCGTGGCATCTGACGCCGGCGCTGCCCGCGCCTTCCT
CAAGGCCCACGACGCGCAGTTCGCCAACCGCCCGAACGTCATCAGCGCCATGGACGTCACCTATCACC
GCCAGAACATGGTGTTCGCCGACTACGGCCCCAAGTGGAAGCTCCTCCGCAAGCTCTGCAGCCTCCAC
CTCCTGGGCGGCAAGGCGCTTGCCGACTGGGCCCCGGTGCGCCGCGCCGAGTTCGGCCACATGGTCGT
GTCCATGCACCGAGCGGCCGCGGAGGGCCGCCCTGTGGTGCTGCCGGAGATGCTGGTGTGCGCCCTGG
CCAACATCATTGGCCAGATAGTGGTGAGCAAGCGGGTGTTCGACGTCCAGGGCATCGAGTCGAACCAT
TACAAGGACATGATCGTGGAGCTGCTGACTGGGGGCGGGCTGTTCAACATCGGCGACTTCGTGCCGGC
TATCGCGTGGATGGACCTGCAGCGGGTACAGGCGAAGATGCGGAGGGTGCATGTTCGGTTCGACGCCA
TGGTGACGAAGCTGTTGGAGGAGCACGAGACCACCAAGGAGGAGCGCAGAGGGAGGCCGGACTTCATC
GACACTATCATGGCCAACAGGGAGGGAGAGGACGGCGAGACCATCACGGACGTCAACGTCAAGGCTAT
CATCTTTGACCTGTTCACAGCCGGCACCGACACGTCGGCGGTCATCGTGGAGTGGGCGCTGGCGGAGA
TGCTGAAGAACCCGAGCATCCTCCGCCGCCTGCAATCCGAGATCGACTACGTGGTGGGCCGAGGCCGC
ATGGTCCAGGAATCCGACCTGCCCAAGCTCCCCTACCTGCACGCGGTGTGCAAGGAAGCGCTGCGCCT
GCACCCGTCCACACCTCTCGGCCTCCCCCACTTCTCCTTCGAGGAGTGCGACGTGAACGGCTACTACA
TCCCCGGCAACACCCGCCTCCTCGTCAACATCTGGGCCATCGGGCGCGACCCCGCCGCGTGGGACGAC
CCCCTCGCGTTCGACCCCGACCGCTTCGTCTCCGGCAAGGCGGCCAAGATCGACCCGCAGGGCAACGA
CTTCGAGCTGATACCGTTCGGGGCCGGGCGGCGGATCTGCGCGGGGAAGCTCGTGGGCATGGTGTTCG
TGCAGTACATGCTGGCGACGTTGGTGCACTCGTTCGACTGGAAGCTGCCGGAGGGGGAGGAGCTCGAC
ATGGAGGAGAAGTTTGGGTTGGCGCTCCCCAAGGCTGTGCCTGTCAAGGCGCTTCTGAGCCCACGCTT
GGCGACTGAAGCATACATTTGA

>GSMUA_Achr8P05810_001_(MaF3'5'H2)_Protein

MQIDPFIAATAALCLVIHHLLRRFLHRSPSRLPLPPGPRGYPVLGALPLVGLQAHTGLARLAQRYGPI
MYLKMGSCGCVVASDAGAARAFLKAHDAQFANRPNVISAMDVTYHRQNMVFADYGPKWKLLRKLCSLH
LLGGKALADWAPVRRAEFGHMVVSMHRAAAEGRPVVLPEMLVCALANIIGQIVVSKRVFDVQGIESNH
YKDMIVELLTGGGLFNIGDFVPAIAWMDLQRVQAKMRRVHVRFDAMVTKLLEEHETTKEERRGRPDFI
DTIMANREGEDGETITDVNVKAIIFDLFTAGTDTSAVIVEWALAEMLKNPSILRRLQSEIDYVVGRGR
MVQESDLPKLPYLHAVCKEALRLHPSTPLGLPHFSFEECDVNGYYIPGNTRLLVNIWAIGRDPAAWDD
PLAFDPDRFVSGKAAKIDPQGNDFELIPFGAGRRICAGKLVGMVFVQYMLATLVHSFDWKLPEGEELD
MEEKFGLALPKAVPVKALLSPRLATEAYI

>GSMUA_Achr8T08630_001_(MaF3'5'H3)_Genomic

CCGTTGCCCACGGCTACCAGTCTCCAAGTGACACACAGATCTATATAGGTCTCCCGTGCCACCATCGC
TCCCACAAGACAGGGTTGATTCCACCTTCCACCATGGCGACGGTCTTCCTGCGCGAGCTCTTTCTATG
CGTCGCGCTCTTTTATGCTCTTCGGTACGTGGTTCGGCGTCTCCTCCCTCGTCGTGCTCTACCACCGG
GGCCGCGAGGGTTCCCCGTCGTCGGTGCGCTCCCGCTGCTGGGAAGCACGCCGCACGTCGCGTTGGCC
CGCATGGCCAAGCGGTACGGCCCGGTCATGCACCTCAAAATGGGGCAGTTCGGCGTGGTCGTGGCTTC
TACTCCGAGTGCGGCTCGCGTCTTCCTCAAGACCCTCGATACCTACTTCTCCAACCGCCCCGTCGACG
CCGCCCCCATCAGGCTCGCCTACGAGGGCCAGGACCTTGTTTTTGCGGAATACGGCCCCAAGTGGAAG
CTGCTGCGCAAACTGTGCAACCTGGAGATGCTCGGCAACAAGGCGCTCGACGCATGGAGCGGCGTGCG
GCGCGACGAGGTCGGCCACATGCTGCGGTCCATGAAAGCGTCGGGACGTAAGCGGGAGAGGGTGATGC
TGGGGGAGATGCTCATTTACACCATGGCGAACATGATCGGGCGGGTGATACTGAGTCGCCGGGTGTTC
GAGACGAAGGACACGCAGGCGAACGAGTTCAAGGACATGGTGGTGGAGCTCATGACTTTGGCGGGGCA
GGTGAACATCGGCGACTTCATCCCGGCGGTGGCGTGGATGGACCTGCAGGGGTTGGAGCGACGGATGA
AGAAGCTTCACAACAAGTTCGATTCGGTGCTCAGTAGGATGGTGGCCGAGCACGAGGCGACCAAAAGC
GACCGTGAAGGGAGACCCGATTTGCTGGACACCGTGCTGACCAACCGGGACGGTTCGGACGAGGTGAA
GCTGACGAACGATAACATCAAGGCCCTGCTTTTGGTAACGAAACCTTTCTGCTCCTCGTCCACAAAAG
CGCTTCTTTGACACGGCATCACGATGTGGTTTTCCTGGTTGCAGAATCTGTTCACTGCAGGAACGGAC
ACCTCGACAGGGACGATCGAATGGGCGATGGCGGAGATGCTACTGAACCCGAGCATCCTCAAGCGGGC
GCAAGCGGAGATGGACAGAGTCATCGGCCGGGACCGGCGACTGGAGGAATCCGACATACCAAACCTCC
CGTACCTGCAAGCCATATGCAAGGAATCGTTCCGGAAGCACCCGTCCACCCCTCTCAACCTGCCGCGC
ATCTCGACCCAGGCGTGCGAGGTCAACGGCTACTACATCCCCAAGAACACCAAGCTGTTGGTCAACAT
ATGGGCGATCGGGAGGGACCCGGACGTGTGGGAAAACCCGCTGGAGTTCAATCCCGACCGGTTCATGA
CGGCGGAGGGATCCAAGATGGACCCGCGCGGCAACGACTTCGAGCTCATCCCCTTCGGCGCCGGGCGA
AGGATCTGCGCCGGCGTGCGCATGGGGGTCGTGCTCGTCCAGTATATGTTGGGTTCTTTGATCCACGC
CTTCAACTGGAATCTGCCGGAAGGGGAGAACCTCGATATGGGCGAGACGTTTGGGATCGCGTTGCAGA
AGACCGTGCCGGTGGCGGCCATGGTGAGCCCTCGATTGGCAGCGAGCATCTACGAGTAATCGACGACG
TGTGGAGATGATCAGAACAGTGGATGAGTCCGTCACCTTTAAGAATAAGGTTGGAAGATAGTGGTCGA
TCGGGTGTGTAAGACTAGTCTGTTGCGACACTGCGTCGAATTTACCATATGAAATCTATAAGATTTGA
AGGCATCTTTTCTTCTATGTTATGATGTTTTATGATACCTATGATATAATTTTGATGTATGAGA

>GSMUA_Achr8T08630_001_(MaF3'5'H3)_Modified CDS

ATGGCGACGGTCTTCCTGCGCGAGCTCTTTCTATGCGTCGCGCTCTTTTATGCTCTTCGGTACGTGGT
TCGGCGTCTCCTCCCTCGTCGTGCTCTACCACCGGGGCCGCGAGGGTTCCCCGTCGTCGGTGCGCTCC
CGCTGCTGGGAAGCACGCCGCACGTCGCGTTGGCCCGCATGGCCAAGCGGTACGGCCCGGTCATGCAC
CTCAAAATGGGGCAGTTCGGCGTGGTCGTGGCTTCTACTCCGAGTGCGGCTCGCGTCTTCCTCAAGAC
CCTCGATACCTACTTCTCCAACCGCCCCGTCGACGCCGCCCCCATCAGGCTCGCCTACGAGGGCCAGG
ACCTTGTTTTTGCGGAATACGGCCCCAAGTGGAAGCTGCTGCGCAAACTGTGCAACCTGGAGATGCTC
GGCAACAAGGCGCTCGACGCATGGAGCGGCGTGCGGCGCGACGAGGTCGGCCACATGCTGCGGTCCAT
GAAAGCGTCGGGACGTAAGCGGGAGAGGGTGATGCTGGGGGAGATGCTCATTTACACCATGGCGAACA

TGATCGGGCGGGTGATACTGAGTCGCCGGGTGTTCGAGACGAAGGACACGCAGGCGAACGAGTTCAAG
GACATGGTGGTGGAGCTCATGACTTTGGCGGGGCAGGTGAACATCGGCGACTTCATCCCGGCGGTGGC
GTGGATGGACCTGCAGGGGTTGGAGCGACGGATGAAGAAGCTTCACAACAAGTTCGATTCGGTGCTCA
GTAGGATGGTGGCCGAGCACGAGGCGACCAAAAGCGACCGTGAAGGGAGACCCGATTTGCTGGACACC
GTGCTGACCAACCGGGACGGTTCGGACGAGGTGAAGCTGACGAACGATAACATCAAGGCCCTGCTTTT
GAATCTGTTCACTGCAGGAACGGACACCTCGACAGGGACGATCGAATGGGCGATGGCGGAGATGCTAC
TGAACCCGAGCATCCTCAAGCGGGCGCAAGCGGAGATGGACAGAGTCATCGGCCGGGACCGGCGACTG
GAGGAATCCGACATACCAAACCTCCCGTACCTGCAAGCCATATGCAAGGAATCGTTCCGGAAGCACCC
GTCCACCCCTCTCAACCTGCCGCGCATCTCGACCCAGGCGTGCGAGGTCAACGGCTACTACATCCCCA
AGAACACCAAGCTGTTGGTCAACATATGGGCGATCGGGAGGGACCCGGACGTGTGGGAAAACCCGCTG
GAGTTCAATCCCGACCGGTTCATGACGGCGGAGGGATCCAAGATGGACCCGCGCGGCAACGACTTCGA
GCTCATCCCCTTCGGCGCCGGGCGAAGGATCTGCGCCGGCGTGCGCATGGGGGTCGTGCTCGTCCAGT
ATATGTTGGGTTCTTTGATCCACGCCTTCAACTGGAATCTGCCGGAAGGGGAGAACCTCGATATGGGC
GAGACGTTTGGGATCGCGTTGCAGAAGACCGTGCCGGTGGCGGCCATGGTGAGCCCTCGATTGGCAGC
GAGCATCTACGAGTAA

>GSMUA_Achr8P08630_001_(MaF3’5’H3)_Protein

MATVFLRELFLCVALFYALRYVVRRLLPRRALPPGPRGFPVVGALPLLGSTPHVALARMAKRYGPVMH
LKMGQFGVVVASTPSAARVFLKTLDTYFSNRPVDAAPIRLAYEGQDLVFAEYGPKWKLLRKLCNLEML
GNKALDAWSGVRRDEVGHMLRSMKASGRKRERVMLGEMLIYTMANMIGRVILSRRVFETKDTQANEFK
DMVVELMTLAGQVNIGDFIPAVAWMDLQGLERRMKKLHNKFDSVLSRMVAEHEATKSDREGRPDLLDT
VLTNRDGSDEVKLTNDNIKALLLNLFTAGTDTSTGTIEWAMAEMLLNPSILKRAQAEMDRVIGRDRRL
EESDIPNLPYLQAICKESFRKHPSTPLNLPRISTQACEVNGYYIPKNTKLLVNIWAIGRDPDVWENPL
EFNPDRFMTAEGSKMDPRGNDFELIPFGAGRRICAGVRMGVVLVQYMLGSLIHAFNWNLPEGENLDMG
ETFGIALQKTVPVAAMVSPRLAASIYE

>GSMUA_Achr9T17300_001_(MaF3’5’H4)_Genomic

TTCATGTCTGTACATTTTATTTCTTGTATAGTTTAATGTTAAAGATTGCTATGAGCGCAGAAAAGTAA
AAAATTGATGTTCACATTTTTTTACTCTCTGAGCGGCTATTTGTAGCACTTCCGGGCTTCATCACGAG
GAGAAGACTCTCATAGATTGCACCATCGGCAGCGGTATGGCGATCGATCCCTACCTCGTGGCCGCCAC
CGTGCTCTGCCTCCTCGTCCACCTCCTCCTCCACCGCTTCCTCCGCAAGTCGCCTTCCCGCTTCCCCT
ACCCGCCGGGCCCCCGGGGGCTCCCCATCCTCGGCTCGCTTCTCCTCGTCGGGGCCAGCGCGCACTCC
AGCCTCGCCCGCCTCGCCGAGCGCTACGGCCCCATCATGTTCCTCCGGCTGGGCTCTCATGGCTGCGT
CGTGGCCTCCAACGCCGACGCTGCACGCGCCTTCCTCAAGACCGTCGACGCCCAGTTCGCCAACCGCC
CCGACCCCATCAGCGCCCGCGACGTCAGCTACCAGCGCCAGAACCTGGCGATGGCCGACTACACCCCG
ACGTGGAAGCTCCTCCGCAAGATGTGCAGCCTGCACCTCCTCGGCGGCAAGGCCTTCGTCGACTGGGC
CCCTGTCCGCCGGGACGAGTTCCGGCGCATGGCCCGCTCCCTGCACGGCCTGGCCGAGGCCGGCGAGC
CGGTGGAGCTGATGGACGTGCTGGTGTGCACGCTGGCCAACGTCGTCGGGTTGATCTTGGTGAGCAGG
CGGGTGTTCGACGCCCACGGGGAGGAGTCCAACAAGTTCAAGGACATATTGGTGGACATGCTGACCGG
CGGCGCGCAGTTCAACATCGGCGACTTCTTCCCGTCGATCGCGTGGATGGACCTGCAGGGGATACAGA
AGAAGATGTTGAGCGTGCACTTGAGGTTCGACGCCATGGTGACGAAGCTGTTCGAGGAACACGAGGCG
GCCAAGGGGGAGCGGCAGGGGAGGCTGGACTTCATCGACAAGGTCATGGCCAACAAGGTGACGGAGGA
CGGGGAGACCATCTCGGAAGTCAACGTTAAAGCACTCATCTTCGTAAGTTTTCCGATGGGAATCATCT
CTTCAAATTACTGCTTTGTTATCTTCGATGAACAGTTTCTCTGGGTCAACGGTATTATCCATTGTTTG
TCGAGTTCATAACAACATGATCACCACCAGATTCCGGCCTCCTCCTATATCTTCGTTTGATAGCAACT
TTTTCACCTTTCCACGCTCGTGCTTTGGGCTTTCTTGGACACTTATCGGCTGTGTCTCGTCGTATACA
CATCAAGAATGAAGATGGTAGTGGAATGTCAACGCCGTAATGAAGTGTTTGATACGACTTTTGTTCTG
TTCGATACTTAGCCTATTAATGAAGTCAGGCTCTTCTGCGTACACTTGTCCGGGCAAGATTGAGCAAA
ACGAGCAGAAAGTAAGGGAGTGATTTAGCCAATGACTTAGTTTTCAATACGATGTTGGTGGCAGGGTA

GATAGATATGATAAGATTGACAGCTTGGATCATTTGGGTGTCTGTTTTGAGGCTCTCTTGATTCCATT
CCATGTAATGCATTTCTCTGAGTTGATCTGACTCGAACAGTGTGCATAACAAGCTGTGTTGATGCAGG
ACCTGTTCACGGCCGGCACCGATACCTCCGCCATCATCGTCGAGTGGGCCATGGCGGAAATGCTCAAG
AACCCGGCGATCCTCAGCCGCGCGCAGGCGGAGTTGGACGATGTGGTCGGCCGCGACCGCCTGCTGGA
GGAAACCGACCTGCCGAAGCTCGCATACCTGCAGGCGGTATGCAAGGAGGCGATGCGGCTGCACCCGT
CCACCCCTCTCAGCCTCCCCCACTTCTCCCACGAGGACTGCGAGGTGAACGGCTACTACATCCCCAAG
AACACCCGGCTCCTCGTCAACATCTGGGCCATCGGGCGGGACCCGGAGGTGTGGGAGGAGCCGCTGGT
GTTCGACCCCGACCGCTTCATCACCGGCAAGGGCGCGAGGTACGATCCGCAGGGGAACGACTTCGAGT
TCATCCCCTTCGGGGCGGGGAGAAGGGTGTGCGCGGGGAAGCTGGTGGGCATGGTGTTCGTTCAGTAC
CTGTTGGGCATGCTAGTGCACGCCTTCGACTGGAGCCTTCCCGACGGCGAGGAGCTCAACATGGACGA
GAAGTTTGGCCTGGCTCTTCCCAAGGCTGTGCCTCTCAAAGTTTTTCTGCGCCCGCGCCTGTCGCCGG
CGGCCTACGCCTGATCTCCCTCTCTATCGATCTATCTATCTATCTCTCGCTCTCTCACCTCAGAAATT
TTAAGGTAGCTGTATAAGAACGTCGCTTCTCTACCTGTGATACGATTGCCGGCAAAACTATTGTCGAT
ATGTGTAGCTACATCAAAAGACTGAGACTGGGATGGCTTGAAGAACCATAGAATATGAATGAATACAC
CTGCATCGTTACACGACAGATGTTGGCAGCTAGACTTGCTTTGATGACAATCATCATGTTGTTGTTGT
TGTAGATCAAGATGGACTGAGGCCTGAACTGGCTGCATTGGCTTTTGGCATGCTTGAGAACATTTGGA
TTACTGACGACTTGTTCATCGTTCTTAAAAAATTAATTGAAAATATCACTTGTGTAACAATATATTAA
TAAAGATCTCAAAATAGATATTCTAATGTCATCTTAAA

>GSMUA_Achr9T17300_001_(MaF3’5’H4)_Modified CDS

ATGGCGATCGATCCCTACCTCGTGGCCGCCACCGTGCTCTGCCTCCTCGTCCACCTCCTCCTCCACCG
CTTCCTCCGCAAGTCGCCTTCCCGCTTCCCCTACCCGCCGGGCCCCCGGGGGCTCCCCATCCTCGGCT
CGCTTCTCCTCGTCGGGGCCAGCGCGCACTCCAGCCTCGCCCGCCTCGCCGAGCGCTACGGCCCCATC
ATGTTCCTCCGGCTGGGCTCTCATGGCTGCGTCGTGGCCTCCAACGCCGACGCTGCACGCGCCTTCCT
CAAGACCGTCGACGCCCAGTTCGCCAACCGCCCCGACCCCATCAGCGCCCGCGACGTCAGCTACCAGC
GCCAGAACCTGGCGATGGCCGACTACACCCCGACGTGGAAGCTCCTCCGCAAGATGTGCAGCCTGCAC
CTCCTCGGCGGCAAGGCCTTCGTCGACTGGGCCCCTGTCCGCCGGGACGAGTTCCGGCGCATGGCCCG
CTCCCTGCACGGCCTGGCCGAGGCCGGCGAGCCGGTGGAGCTGATGGACGTGCTGGTGTGCACGCTGG
CCAACGTCGTCGGGTTGATCTTGGTGAGCAGGCGGGTGTTCGACGCCCACGGGGAGGAGTCCAACAAG
TTCAAGGACATATTGGTGGACATGCTGACCGGCGGCGCGCAGTTCAACATCGGCGACTTCTTCCCGTC
GATCGCGTGGATGGACCTGCAGGGGATACAGAAGAAGATGTTGAGCGTGCACTTGAGGTTCGACGCCA
TGGTGACGAAGCTGTTCGAGGAACACGAGGCGGCCAAGGGGGAGCGGCAGGGGAGGCTGGACTTCATC
GACAAGGTCATGGCCAACAAGGTGACGGAGGACGGGGAGACCATCTCGGAAGTCAACGTTAAAGCACT
CATCTTCGACCTGTTCACGGCCGGCACCGATACCTCCGCCATCATCGTCGAGTGGGCCATGGCGGAAA
TGCTCAAGAACCCGGCGATCCTCAGCCGCGCGCAGGCGGAGTTGGACGATGTGGTCGGCCGCGACCGC
CTGCTGGAGGAAACCGACCTGCCGAAGCTCGCATACCTGCAGGCGGTATGCAAGGAGGCGATGCGGCT
GCACCCGTCCACCCCTCTCAGCCTCCCCCACTTCTCCCACGAGGACTGCGAGGTGAACGGCTACTACA
TCCCCAAGAACACCCGGCTCCTCGTCAACATCTGGGCCATCGGGCGGGACCCGGAGGTGTGGGAGGAG
CCGCTGGTGTTCGACCCCGACCGCTTCATCACCGGCAAGGGCGCGAGGTACGATCCGCAGGGGAACGA
CTTCGAGTTCATCCCCTTCGGGGCGGGGAGAAGGGTGTGCGCGGGGAAGCTGGTGGGCATGGTGTTCG
TTCAGTACCTGTTGGGCATGCTAGTGCACGCCTTCGACTGGAGCCTTCCCGACGGCGAGGAGCTCAAC
ATGGACGAGAAGTTTGGCCTGGCTCTTCCCAAGGCTGTGCCTCTCAAAGTTTTTCTGCGCCCGCGCCT
GTCGCCGGCGGCCTACGCCTGA

>GSMUA_Achr9P17300_001_(MaF3’5’H4)_Protein

MAIDPYLVAATVLCLLVHLLLHRFLRKSPSRFPYPPGPRGLPILGSLLLVGASAHSSLARLAERYGPI
MFLRLGSHGCVVASNADAARAFLKTVDAQFANRPDPISARDVSYQRQNLAMADYTPTWKLLRKMCSLH
LLGGKAFVDWAPVRRDEFRRMARSLHGLAEAGEPVELMDVLVCTLANVVGLILVSRRVFDAHGEESNK
FKDILVDMLTGGAQFNIGDFFPSIAWMDLQGIQKKMLSVHLRFDAMVTKLFEEHEAAKGERQGRLDFI
DKVMANKVTEDGETISEVNVKALIFDLFTAGTDTSAIIVEWAMAEMLKNPAILSRAQAELDDVVGRDR
LLEETDLPKLAYLQAVCKEAMRLHPSTPLSLPHFSHEDCEVNGYYIPKNTRLLVNIWAIGRDPEVWEE

PLVFDPDRFITGKGARYDPQGNDFEFIPFGAGRRVCAGKLVGMVFVQYLLGMLVHAFDWSLPDGEELN
MDEKFGLALPKAVPLKVFLRPRLSPAAYA

>GSMUA_Achr11T05200_001_(MaF3’5’H5)_Genomic

TTCGTCGAGGTCGTTCCATAAGTCCACCATTACCGCCATGGATATGGCAACAACCTTCCT
ACGTGAGCTTTTCCTATGCGTTGCGCTCTACTACATCCTTCGCCACGTCGTCTCCCGCCT
CCTGTCGTGTTTTTCTCCTCTCCCTCCGGGACCGCGAGGCTTCCCGGTGGTGGGTGCCCT
CCCGCTGCTGGGAAGCGCGCCGCACGTCACACTGGCCGGCATGGCCAAGCGCTACGGCCC
CGTCATGCACCTCAAAATGGGGCAGTTCGGGATGGTGGTGGCGTCTACTCCGGACGCCGC
CCGAGTCTTCCTCAAGACTCTCGATCCTTACTTCTCGAACCGCCCCGTCGACGCCGCCTC
CGTCCGCCTCGCCTACGGTGGTCAGGACCTTGTTTTCGCCGAGTACGGCCCCAAGTGGAA
GTTGCTGCGCAAACTCTGCAAATTGGAGATGCTCGGCAACAAGGCGCTCGAGTCATGGGC
GGCGGTGCGGCGCGACGAGGTCGGCCGCATGTTGCGATCCATGCATGCATCGGGGCGGAA
GGGAGAGCCGGTCATGCTAGGGGGGATGCTAATCTATACCATAGCCAACATGATCGGGCG
GGTGATACTGAGCGGGCGGGTGTTCGAGACGAAGGGCTCGGAGGCGAACGAGTTCAAGGA
CATGGTGGTGGAACTCATGACATTAGCGGCGCAAGTCAACATCAGTGACTTCTTGCCGGC
GGTGGCTTGGATGGACCTCCAGGGGCTGGAGGCGCGGATGAAGAAGCTGCACAAGAAGTT
CGATCGGGTTCTCAGCAGGATGGTGGCGGAGCACGAGGCGTCAAAGGGTGAACGTGAGGG
GAGACCGGACTTGCTTGACGCAGTGATGGCGATCCGGGACGGGCCAGCGGAGGAGAAGCT
GACCAACGATAACGTCAAGGCTCTTCTTTTGGTAAAGACCACCATTCCCGCTCATGCAAA
CCACGACGCATGCACAGTCTAATTTCTGGCTTCTGGCCGTGTCAGAATCTGTTCACCGCA
GGCACCGACACCTCGAAGGGCACCATAGAATGGGCAATGGCCGAAATGCAACTGAACCCA
AGCATCCTCAGGCGAGCCCAGGCCGAGATGGACGGAATCATCGGCCGAAACCGGCGTCTG
GAGGAATCCGACATACCAAACCTCCCGTACCTTCAAGCCATATGCAAGGAATCATTCCGG
AAGCACCCATCCACGCCTCTCAACCTCCCACGGATCTCAACCCAGGCGTGCGAGGTCAAC
GGCTATTACATCCCGAAGAACACGAAGCTCTTCGTCAACGTATGGGCCATCGGGAGGGAC
GCGGACGTGTGGGAGCACCCACTAGAGTTCAACCCCGACCGCTTCATGACGGCGAAGGGC
GCCAAGATAGACCCTCGTGGCAATGACTTCGAGCTCATACCGTTCGGCGCCGGGCGGAGG
ATCTGCGCCGGTGTGCGCGCGGGGGTTGTGCTCGTGCAGTACATGTTGGGTTCGCTGATC
CACTCGTTCGACTGGAGTCTACCCGAAGGGTCAAAGCCCGACATGGGCGAGACGTTTGGG
ATCGCACTGCAAAAGACTGTGCCGGTGGCGGCCATGGTGAGCCCTCGATGTGCTCCCAGC
GTCTACGACTAGTTGAAACCCCATGCATGTTGCCTCAACTTGGAAGACGGTAGTGAGTCA
GTCTGGTACGAGACATCTCCACGGAATGCT

>GSMUA_Achr11T05200_001_(MaF3’5’H5)_Modified CDS

ATGGCAACAACCTTCCTACGTGAGCTTTTCCTATGCGTTGCGCTCTACTACATCCTTCGCCACGTCGT
CTCCCGCCTCCTGTCGTGTTTTTCTCCTCTCCCTCCGGGACCGCGAGGCTTCCCGGTGGTGGGTGCCC
TCCCGCTGCTGGGAAGCGCGCCGCACGTCACACTGGCCGGCATGGCCAAGCGCTACGGCCCCGTCATG
CACCTCAAAATGGGGCAGTTCGGGATGGTGGTGGCGTCTACTCCGGACGCCGCCCGAGTCTTCCTCAA
GACTCTCGATCCTTACTTCTCGAACCGCCCCGTCGACGCCGCCTCCGTCCGCCTCGCCTACGGTGGTC
AGGACCTTGTTTTCGCCGAGTACGGCCCCAAGTGGAAGTTGCTGCGCAAACTCTGCAAATTGGAGATG
CTCGGCAACAAGGCGCTCGAGTCATGGGCGGCGGTGCGGCGCGACGAGGTCGGCCGCATGTTGCGATC
CATGCATGCATCGGGGCGGAAGGGAGAGCCGGTCATGCTAGGGGGGATGCTAATCTATACCATAGCCA
ACATGATCGGGCGGGTGATACTGAGCGGGCGGGTGTTCGAGACGAAGGGCTCGGAGGCGAACGAGTTC
AAGGACATGGTGGTGGAACTCATGACATTAGCGGCGCAAGTCAACATCAGTGACTTCTTGCCGGCGGT
GGCTTGGATGGACCTCCAGGGGCTGGAGGCGCGGATGAAGAAGCTGCACAAGAAGTTCGATCGGGTTC
TCAGCAGGATGGTGGCGGAGCACGAGGCGTCAAAGGGTGAACGTGAGGGGAGACCGGACTTGCTTGAC
GCAGTGATGGCGATCCGGGACGGGCCAGCGGAGGAGAAGCTGACCAACGATAACGTCAAGGCTCTTCT
TTTGAATCTGTTCACCGCAGGCACCGACACCTCGAAGGGCACCATAGAATGGGCAATGGCCGAAATGC
AACTGAACCCAAGCATCCTCAGGCGAGCCCAGGCCGAGATGGACGGAATCATCGGCCGAAACCGGCGT
CTGGAGGAATCCGACATACCAAACCTCCCGTACCTTCAAGCCATATGCAAGGAATCATTCCGGAAGCA

CCCATCCACGCCTCTCAACCTCCCACGGATCTCAACCCAGGCGTGCGAGGTCAACGGCTATTACATCC
CGAAGAACACGAAGCTCTTCGTCAACGTATGGGCCATCGGGAGGGACGCGGACGTGTGGGAGCACCCA
CTAGAGTTCAACCCCGACCGCTTCATGACGGCGAAGGGCGCCAAGATAGACCCTCGTGGCAATGACTT
CGAGCTCATACCGTTCGGCGCCGGGCGGAGGATCTGCGCCGGTGTGCGCGCGGGGGTTGTGCTCGTGC
AGTACATGTTGGGTTCGCTGATCCACTCGTTCGACTGGAGTCTACCCGAAGGGTCAAAGCCCGACATG
GGCGAGACGTTTGGGATCGCACTGCAAAAGACTGTGCCGGTGGCGGCCATGGTGAGCCCTCGATGTGC
TCCCAGCGTCTACGACTAG

>GSMUA_Achr11P05200_001_(MaF3’5’H5)_Protein

MATTFLRELFLCVALYYILRHVVSRLLSCFSPLPPGPRGFPVVGALPLLGSAPHVTLAGMAKRYGPVM
HLKMGQFGMVVASTPDAARVFLKTLDPYFSNRPVDAASVRLAYGGQDLVFAEYGPKWKLLRKLCKLEM
LGNKALESWAAVRRDEVGRMLRSMHASGRKGEPVMLGGMLIYTIANMIGRVILSGRVFETKGSEANEF
KDMVVELMTLAAQVNISDFLPAVAWMDLQGLEARMKKLHKKFDRVLSRMVAEHEASKGEREGRPDLLD
AVMAIRDGPAEEKLTNDNVKALLLNLFTAGTDTSKGTIEWAMAEMQLNPSILRRAQAEMDGIIGRNRR
LEESDIPNLPYLQAICKESFRKHPSTPLNLPRISTQACEVNGYYIPKNTKLFVNVWAIGRDADVWEHP
LEFNPDRFMTAKGAKIDPRGNDFELIPFGAGRRICAGVRAGVVLVQYMLGSLIHSFDWSLPEGSKPDM
GETFGIALQKTVPVAAMVSPRCAPSVYD

>GSMUA_Achr11T15630_001_(MaF3’5’H6)_Genomic

AGAACTCTGCCGTTGACTTGTATGATGTACTGTATCGTGTCCTGCCACCCATGACAAGCAAATCGATC
AGATATAGTGCTGTCGTTATACATGTTTGCAGGCATCACATGTAGGCGCTGTCCGCAAGGCGTGGGCT
AACTGCGGTCTTGAGAGGCACTGCCTTGGGGAGCACGAGGCCAAACCGCTCCTCCATGTTGAGCTCTT
CCACGTCGTCGGCGAGCTTCCAGTCGAAGGAGTGCACCAAGGAGCCGAGGGTGTACTGCACAAAGACG
AGCCCGAGCTGCATCCCTGCGCAGATCCTTCGGCCGGCACCGAAGGGAATCAGCTCGAAGTCGTTGCC
CAGCGGCTCGATGTGTGCGTTTTTGCCGCTCAAGAACCTCTCCGGCTTGAACTCCAGAGGGTCCTCCC
ACACGTTGGGGTCCCTCCCTATGGCCCAGATGTTGATGAGGAGCCTGGTTTTCTCGGGGATGTAGTAG
CCGTCAACCTCGCACGATTCGAACGAGTAGTGTGGGATGCCCAGCGGCGTGGAAGGATGCAGCCGCAG
CGCCTCTTTGCAGATGGCTCGCAGGTAGGGGAGTTTGGGTATGTCGGACTCTTCCAGCCTCCGGCTTT
TGCCGATCACTTGGTCCATCTCAGCTTGAGCCTTCTTCAGTATGGTTGGGTTCTTCAGCATCTCCGCC
AGCGCCCACTCGATTGTGACGGAGGATGTATCGGTTCCAGCGACGAACATGTCCTACGAGGAAAAGGA
CAAGGTTACATCTCTGTCAAATCATGACACTTGTGGTGATGCGTGAGGAGAGACGAGCATGATGTGTT
TGTAAACGCCGGGGATAGTTGGTTGTTCATATGATACTAATATGTTATGGGAATTATTGTGGAGGGGA
GAAGGAGCTCACGAAGATGAAGCCTCGGATGTTGACGTCGGAAAGCGACACGCCATCGGCGTCCACCT
TGTTGGCCAGCAAAAGGTCGAGCACGTCCGGCCTCCCCTTGCGCTCGTGCGCCGTCTCCTGGTGCTCG
GCCACGAACTCGGAGAGGATGGCGTCCATCCTCAGGTGGGCGCGGCGCAGGTGGCGCTGCACCCCCTG
CAGATCCATCCACGCGATGGCCGGCACGAAGTCGCTGATGTTGAACAACCCCACCCCTGTCAGCATCT
CCGTGACCGCGTGCTTGAACTTGCTCGACTCCTCGCCCTGCGCCGCGAACACCCGCCGCGACAGCACC
ACCTGGCCGATTATGTTGGCGTTGGAGCACACCACCGCCTCCTGGACGAACACCGGCCGGGAGTTTTT
GCTCGACTCGCGCATGGAGCGGAGCAGGCGGCCGACCTCGTCGGCCCGGACCTGTGCCTGTTCCGCGA
TTGCCTTGGGGCCGAGAAGGTGGAGGTTAGAGAACCTGCGGAGAAGCTTCCACCGAGGCCCGTAGTTG
GAGAACACGAAGTTCTGGCCGTCGTAGGTGACGTCCTTCCCGCTGATGGTGGAGGGGCGGTTGGCGAA
CTGGAGGTCGCGCGCCTTGAGGAAGGACCGAGCGGCGCCAGCCGTGGAGGCCACCACGACCCGCACGG
TGCCCATCTTGAGGTGCATGATGGGGCCGTAGCGCTTGGCGAGTTCAGCCAGCGAGGCGTGGGCCATG
GGACCTATCTGCGGCAGCGCGCCGAGCACCGGAATGCCCTTGGGCCCGGGGGGAAGCGGCAACCGTGG
GTTCGATCGTAGCCTGCGGCGGAGGAGGAGGTGGACGACAACGCTCAAGAGAATGCCGACGATGAGCA
CAGTGTCAACAGCCATGGTGACGGTGGTGCTGCGTTTGCTTGGCTCAATAAAGGTCCGTCTGATGCTT
CATATATATACACATGGAGAACATGCACGGAGGA

>GSMUA_Achr11T15630_001_(MaF3’5’H6)_Modified CDS

ATGGCTGTTGACACTGTGCTCATCGTCGGCATTCTCTTGAGCGTTGTCGTCCACCTCCTCCTCCGCCG
CAGGCTACGATCGAACCCACGGTTGCCGCTTCCCCCCGGGCCCAAGGGCATTCCGGTGCTCGGCGCGC
TGCCGCAGATAGGTCCCATGGCCCACGCCTCGCTGGCTGAACTCGCCAAGCGCTACGGCCCCATCATG

CACCTCAAGATGGGCACCGTGCGGGTCGTGGTGGCCTCCACGGCTGGCGCCGCTCGGTCCTTCCTCAA
GGCGCGCGACCTCCAGTTCGCCAACCGCCCCTCCACCATCAGCGGGAAGGACGTCACCTACGACGGCC
AGAACTTCGTGTTCTCCAACTACGGGCCTCGGTGGAAGCTTCTCCGCAGGTTCTCTAACCTCCACCTT
CTCGGCCCCAAGGCAATCGCGGAACAGGCACAGGTCCGGGCCGACGAGGTCGGCCGCCTGCTCCGCTC
CATGCGCGAGTCGAGCAAAAACTCCCGGCCGGTGTTCGTCCAGGAGGCGGTGGTGTGCTCCAACGCCA
ACATAATCGGCCAGGTGGTGCTGTCGCGGCGGGTGTTCGCGGCGCAGGGCGAGGAGTCGAGCAAGTTC
AAGCACGCGGTCACGGAGATGCTGACAGGGGTGGGGTTGTTCAACATCAGCGACTTCGTGCCGGCCAT
CGCGTGGATGGATCTGCAGGGGGTGCAGCGCCACCTGCGCCGCGCCCACCTGAGGATGGACGCCATCC
TCTCCGAGTTCGTGGCCGAGCACCAGGAGACGGCGCACGAGCGCAAGGGGAGGCCGGACGTGCTCGAC
CTTTTGCTGGCCAACAAGGTGGACGCCGATGGCGTGTCGCTTTCCGACGTCAACATCCGAGGCTTCAT
CTTCGACATGTTCGTCGCTGGAACCGATACATCCTCCGTCACAATCGAGTGGGCGCTGGCGGAGATGC
TGAAGAACCCAACCATACTGAAGAAGGCTCAAGCTGAGATGGACCAAGTGATCGGCAAAAGCCGGAGG
CTGGAAGAGTCCGACATACCCAAACTCCCCTACCTGCGAGCCATCTGCAAAGAGGCGCTGCGGCTGCA
TCCTTCCACGCCGCTGGGCATCCCACACTACTCGTTCGAATCGTGCGAGGTTGACGGCTACTACATCC
CCGAGAAAACCAGGCTCCTCATCAACATCTGGGCCATAGGGAGGGACCCCAACGTGTGGGAGGACCCT
CTGGAGTTCAAGCCGGAGAGGTTCTTGAGCGGCAAAAACGCACACATCGAGCCGCTGGGCAACGACTT
CGAGCTGATTCCCTTCGGTGCCGGCCGAAGGATCTGCGCAGGGATGCAGCTCGGGCTCGTCTTTGTGC
AGTACACCCTCGGCTCCTTGGTGCACTCCTTCGACTGGAAGCTCGCCGACGACGTGGAAGAGCTCAAC
ATGGAGGAGCGGTTTGGCCTCGTGCTCCCCAAGGCAGTGCCTCTCAAGACCGCAGTTAGCCCACGCCT
TGCGGACAGCGCCTACATGTGA

>GSMUA_Achr11P15630_001_(MaF3’5’H6)_Protein

MAVDTVLIVGILLSVVVHLLLRRRLRSNPRLPLPPGPKGIPVLGALPQIGPMAHASLAELAKRYGPIM
HLKMGTVRVVVASTAGAARSFLKARDLQFANRPSTISGKDVTYDGQNFVFSNYGPRWKLLRRFSNLHL
LGPKAIAEQAQVRADEVGRLLRSMRESSKNSRPVFVQEAVVCSNANIIGQVVLSRRVFAAQGEESSKF
KHAVTEMLTGVGLFNISDFVPAIAWMDLQGVQRHLRRAHLRMDAILSEFVAEHQETAHERKGRPDVLD
LLLANKVDADGVSLSDVNIRGFIFDMFVAGTDTSSVTIEWALAEMLKNPTILKKAQAEMDQVIGKSRR
LEESDIPKLPYLRAICKEALRLHPSTPLGIPHYSFESCEVDGYYIPEKTRLLINIWAIGRDPNVWEDP
LEFKPERFLSGKNAHIEPLGNDFELIPFGAGRRICAGMQLGLVFVQYTLGSLVHSFDWKLADDVEELN
MEERFGLVLPKAVPLKTAVSPRLADSAYM

>GSMUA_AchrUn_randomT12120_001_(MaF3’5’H7)_Genomic

TAATGTCTCTCTTAGAGTGATGATGCAATTTTTATCTCCTTTTGTAGATCGTGTTGTTTTCATATGGA
GAGTGTTTGGTGGTTTCGTGGGATTTGTTGGTGAGATCCCATTGTGTGCACTTCTCTCTATAAATCTG
CAAGACAACACCCAACCACCGGTCTCTCCTCTCATTCTGTCCACTTAGTAGACGCACCACCGGCGCAT
CTCCATGGCTCTCGATATTGTTCTCGTCGCCGGCATCCTCTTGAGCGTCCTTGTCCACCTTCTCCTCC
GCCGCAGACTCCAATCCATCCGCCGGCTCCCGCTCCCTCCTGGTCCAGCGGGCATCCCAATCCTCGGC
TCGCTACCGCAGATCGGCCCCATGCCCCACGCCTCGCTCGCTAGCCTCGCCGTACGTTACGGCCCCAT
TATGTACCTCAAGATGGGCACCGGAGGGGTCGTGGTCGCCTCCTCCGCCAGTTCTGCCAGCTCCTTTC
TCAAGGGCCTCGACCTTCAGTTCGCCAACCGCCCCTTCGCCATCAGCCGGAAGGACGTCACCTACGAC
TGCCAGAACTTCGTGTTCGCCAACTACGGGCCTCGCTGGAAGCTCTTCCGCAAGCTCTGTAACCTCCA
CTTCCTAGGCAGCAAGGCGCTGGCCGACTGGGCACCGATTCGCCGCGACGAGATCGGTCGCGTCCTCC
GCTCCATGCTCGAGTCGAGCCGGAACTCGCGGCCGGTGGCGGTGGCGGTGTCGGAGGCACTGGTGTGC
GCCAACGCCAACATCATCGGGCAGGTGCTGCTTTCGCGGCGGGTGTTCGAATCGCAGGGGGAGGAGTC
GAACCAGTTCAAGGACGCGATCACGGAGCTGCTGACGTGGTCGGGGAAGTTCAACATCGGCGACTTCG
TGCCGGCGATCGCGTGGATGGACGTGCAGGGGGTGCAGCGGCACATGCGTCAGCTGCATATCAAGATA
GACGCTCTGATCACGGCTCTCTTGGCGGAGCACGAGGCGACGGCGCACGAGCGCAAGGGGAGGCCGGA
CGTGGTGGATCTTGTGATGGCCAACAGAGTGGACGCCGATGGCGTGTCGCTTTCTGACGTCAACATCA
AGGCCTTTATCTCTGTGAGCTCTCCGTACTCCCCGTCATCACTATAGTATATAGAGAGAGACTGACTG
CTATATCATTTATGGTTCTTTGGTCAAATTATTGTTCTTTGGTTACAGTTTATTTCTTCTTTATTCTT
TCTTTCCCAATCCTTTCCTTCTTTACTGCTTATTCAGTTGACTAACAAAATACCATTACCTCTGCAAC
TCCTTATATCTTGTAGGATATGTTTATCGCCGGAACTGATACATCCTCCGTCATAATCGAGTGGGCGC
TCGCAGAAATGCTAAGGAAGCCAACCATCCTGCAGCGAGCTCAAGATGAGATGGACAGAGTAATCGGG

AAGACCCGTAGGCTTGAAGAGTCTGACATACCAAATCTTCCCTTCCTACGGGCCATATCCAAAGAGGT
GTTACGATTGCACCCTTCGACGCCGCTGAGCCTTCCACACTACACGCCCGAAGCATGTGAGGTGGACG
GCTACTACATCCCCAAAGGCACACGTCTTCTGGTCAACATATGGGCCATCGGAAGAGATCCCAATGTG
TGGGAAGATCCACTGGAGTTTAAACCAGAGAGGTTCTTGAGCGGTAGGAACGCCAACATCGAGCCACT
GGGTCACGACTTCGAGTTGATTCCATTCGGTGCTGGGCGAAGGATCTGCGTGGGGATGCATGCAGGAC
TGCTCATGGTACAGTACGTGCTGGGGACTTTGGTGCACTCGTTCAACTGGAAGCTTGTCGATGACATC
CAAGTGCTGGACATGGAGGAGAAGTTTGGGTTGGTGCTCCCAAAGAAGGTGTCCCTTAAGGCGATAGT
TAGCCCACGCCTCGTGGGAAGTGCCTATATGTGATGTGGGCAAAGAAGTAGAAGAGCCTATCATGGGA
TATATGCATGCATGTATGAGAACCTGGTTTATTTCTTAGGATGGATATCTATATTGTAAATAAATGGA
TACAGTACTTTCTTTATTTAGCTTGAATCCCTACCAATCTTAATTATCAAATTTATTATAAGTTTAAA
TTGATGAAATATAAGAAGATTTAGGAAATGTAAATTAAATTAGTTTAGTCTTTAATTGTCTGATTGGT
GTTTAGTTACTTGCAACTGGAAATTCCTTCAAGTGATAGTTAAAGGCAAAAGCAAGTAAAGTTGTTTG
GATGGTTGCTTGCTTAGTTCTTACAAGAAGTTGCAATCAAGATACAGGAAGCAGCCATTGATGCCTGG
AGAGGAAGGAAGAGGTGGTCGACTTCATCGAGATTTGCACTAAGCAGCAGTAGCTGTTATAATTTTGG

>GSMUA_AchrUn_randomT12120_001_(MaF3’5’H7)_Modified CDS

ATGGCTCTCGATATTGTTCTCGTCGCCGGCATCCTCTTGAGCGTCCTTGTCCACCTTCTCCTCCGCCG
CAGACTCCAATCCATCCGCCGGCTCCCGCTCCCTCCTGGTCCAGCGGGCATCCCAATCCTCGGCTCGC
TACCGCAGATCGGCCCCATGCCCCACGCCTCGCTCGCTAGCCTCGCCGTACGTTACGGCCCCATTATG
TACCTCAAGATGGGCACCGGAGGGGTCGTGGTCGCCTCCTCCGCCAGTTCTGCCAGCTCCTTTCTCAA
GGGCCTCGACCTTCAGTTCGCCAACCGCCCCTTCGCCATCAGCCGGAAGGACGTCACCTACGACTGCC
AGAACTTCGTGTTCGCCAACTACGGGCCTCGCTGGAAGCTCTTCCGCAAGCTCTGTAACCTCCACTTC
CTAGGCAGCAAGGCGCTGGCCGACTGGGCACCGATTCGCCGCGACGAGATCGGTCGCGTCCTCCGCTC
CATGCTCGAGTCGAGCCGGAACTCGCGGCCGGTGGCGGTGGCGGTGTCGGAGGCACTGGTGTGCGCCA
ACGCCAACATCATCGGGCAGGTGCTGCTTTCGCGGCGGGTGTTCGAATCGCAGGGGGAGGAGTCGAAC
CAGTTCAAGGACGCGATCACGGAGCTGCTGACGTGGTCGGGGAAGTTCAACATCGGCGACTTCGTGCC
GGCGATCGCGTGGATGGACGTGCAGGGGGTGCAGCGGCACATGCGTCAGCTGCATATCAAGATAGACG
CTCTGATCACGGCTCTCTTGGCGGAGCACGAGGCGACGGCGCACGAGCGCAAGGGGAGGCCGGACGTG
GTGGATCTTGTGATGGCCAACAGAGTGGACGCCGATGGCGTGTCGCTTTCTGACGTCAACATCAAGGC
CTTTATCTCTGATATGTTTATCGCCGGAACTGATACATCCTCCGTCATAATCGAGTGGGCGCTCGCAG
AAATGCTAAGGAAGCCAACCATCCTGCAGCGAGCTCAAGATGAGATGGACAGAGTAATCGGGAAGACC
CGTAGGCTTGAAGAGTCTGACATACCAAATCTTCCCTTCCTACGGGCCATATCCAAAGAGGTGTTACG
ATTGCACCCTTCGACGCCGCTGAGCCTTCCACACTACACGCCCGAAGCATGTGAGGTGGACGGCTACT
ACATCCCCAAAGGCACACGTCTTCTGGTCAACATATGGGCCATCGGAAGAGATCCCAATGTGTGGGAA
GATCCACTGGAGTTTAAACCAGAGAGGTTCTTGAGCGGTAGGAACGCCAACATCGAGCCACTGGGTCA
CGACTTCGAGTTGATTCCATTCGGTGCTGGGCGAAGGATCTGCGTGGGGATGCATGCAGGACTGCTCA
TGGTACAGTACGTGCTGGGGACTTTGGTGCACTCGTTCAACTGGAAGCTTGTCGATGACATCCAAGTG
CTGGACATGGAGGAGAAGTTTGGGTTGGTGCTCCCAAAGAAGGTGTCCCTTAAGGCGATAGTTAGCCC
ACGCCTCGTGGGAAGTGCCTATATGTGA

>GSMUA_AchrUn_randomP12120_001_(MaF3’5’H7)_Protein

MALDIVLVAGILLSVLVHLLLRRRLQSIRRLPLPPGPAGIPILGSLPQIGPMPHASLASLAVRYGPIM
YLKMGTGGVVVASSASSASSFLKGLDLQFANRPFAISRKDVTYDCQNFVFANYGPRWKLFRKLCNLHF
LGSKALADWAPIRRDEIGRVLRSMLESSRNSRPVAVAVSEALVCANANIIGQVLLSRRVFESQGEESN
QFKDAITELLTWSGKFNIGDFVPAIAWMDVQGVQRHMRQLHIKIDALITALLAEHEATAHERKGRPDV
VDLVMANRVDADGVSLSDVNIKAFISDMFIAGTDTSSVIIEWALAEMLRKPTILQRAQDEMDRVIGKT
RRLEESDIPNLPFLRAISKEVLRLHPSTPLSLPHYTPEACEVDGYYIPKGTRLLVNIWAIGRDPNVWE
DPLEFKPERFLSGRNANIEPLGHDFELIPFGAGRRICVGMHAGLLMVQYVLGTLVHSFNWKLVDDIQV
LDMEEKFGLVLPKKVSLKAIVSPRLVGSAYM

>GSMUA_Achr3T31110_001_(MaDFR1)_Genomic

ATTGTTGATCTACCGTGTCGGAGAATAATCTTAGAGCCACACCTGACGTTATTACGTTTGACTTGCCA
TCACTCTGTGCAACCACAGCTTGACCGTTAGATGCGCCACGTTCCACCTACCGTACCGTCCATTCTTC
CCCCAGTGGTTGCACGCAAGCAGGTAGGCTGACTTCGTATATACATGTCCGAGCGACGCAGGCGAAGA
CGACACTCATCTCAGAGAGAGAGAGAGAGAGAGATGAAGGGGCCGGTGGTGGTGACTGGGGCCTCGGG
ATACATCGGGACATGGCTGGTGATGAAGCTTCTGCAGAAGGGGTACGTGGTCAGGGCCACCGTGCGAG
ACCCCAGTCAGTAGCTGCTCTTTTCGTCTCTTTCTTCTCTCTCTTGTGCGTTTTGTTCGAGCTTCGTT
TGATCCATGTCGGAGTTGGGGAAGATCTAACGTCGTTGCATGCAGCGAACCAGAAGAAGATCAAGCCG
CTGGTGGAGCTGCCGGGATCGGTTGAGCGGCTCACCATCTGGCGAGCGGACTTGGAAGAAGAAGGAAG
CTTCGATGAGGCGGTCAAGGGATGCGAGGGAGTGTTCCATGTGGCCACTCCCATGGATTTCGAGTCCA
AAGACCCTGAGGTAATCTCACGGTACTTTAGAATTCGAACCTTACTTAGATCACGAGCATACGATGCG
TCGTCGGCGGACTCTTTTGCAGAACGAGATCATCAAGCCCACGGTCGACGGAGTGCTGAGCATCATGA
GGTCCTGCAAGGAAGCCGGGACGGTCAGGCGCGTCGTCTTCACTTCCTCCGCCGGCACCGTCAACGTG
CAGGAGCAGCAACAGCCCCAGTACGACGAGAGCTCCTGGAGCGACATCGACTTCTGCCGCCGCGTCAA
GATGACCGGATGGGTAACCTTGCTTCCCATCTTCTTCTCTCGCCTTGCATGCGCTCCGCTTTCGGCTT
CTCAAAGGGATTTCTCTTGTTTACAGATGTACTTCGTGTCCAAATCTCTGGCTGAGAAGGCAGCGTAC
GAGTTTGCGAGGGAGAATGGCATCGACCTCATCAGCATCATCCCGACTCTTGTCGTCGGCCCCTTCAT
CACCACGACCATGCCGCCCAGCATGATCACCGCGTTGTCACTCATCACAGGTTAGTGACAGCAACCTC
TCTCTCTGAGACCTTTCCGCAGAGGAGAATTAGATCGAGCACGCATGCAATGCATTACAGGAAACGAA
GCTCACTATTCGATCTTGAAGCAAGTTCAACTGGTTCACTTGGACGACCTGTGTGATACCCACGTCTT
CCTGTACGAGCACCCCGATGCAAATGGGAGATACATCTGCTCCTCTCATGACGCCACCATATACGATC
TCGCCAAGATGTTCCGAGCGCGATACCCCCAGTACAACATCCCTCAGCAGTAAGTCCTAATCTCCCAT
GGCCATCAAGAGAAAGCCCGATTCAGACAACAAGATCTTACGCTTGCAGGTTCGAAGGGATCGATGAA
ACGATCGAACGGGTGCACTTCTCCTCCAAGAAACTCACCGACCTGGGTTACAAGTTCCGGTACACGAT
GGAGGACATGTTCGATGCCGCCATCGAGTCGTGCTGGGAGAAGCATCTGATACCACTCCGGACAGCAG
AGGAACAGTGCAGTGAGGTGGGCAAACCTCTGCCTCTGGCAACAGAGACACTGAGTGAGTTCAGTGAA
GAGAAGGTCCTGATGGCTTAGGTGGAGACACTCCGAGATGCACCAGACATTGCATGATTTCTTCTCAT
CACAGGAACAAGTCATGTCCTGTGAAGGTTGACTAATTTGTCTTGGAGTGGCCAAAAACCAGAGATAT
ATTTAGCTGCCTCAATTTGATTGTAATCTCTTTGATCGATCTAATAATAATATTTTTAATCGATAATA
ATAAATTAATTTTAATAAATAATAATCTTATATCAAGTGAAAATTTATAATAAATT

>GSMUA_Achr3T31110_001_(MaDFR1)_Modified CDS

ATGAAGGGGCCGGTGGTGGTGACTGGGGCCTCGGGATACATCGGGACATGGCTGGTGATGAAGCTTCT
GCAGAAGGGGTACGTGGTCAGGGCCACCGTGCGAGACCCCACGAACCAGAAGAAGATCAAGCCGCTGG
TGGAGCTGCCGGGATCGGTTGAGCGGCTCACCATCTGGCGAGCGGACTTGGAAGAAGAAGGAAGCTTC
GATGAGGCGGTCAAGGGATGCGAGGGAGTGTTCCATGTGGCCACTCCCATGGATTTCGAGTCCAAAGA
CCCTGAGAACGAGATCATCAAGCCCACGGTCGACGGAGTGCTGAGCATCATGAGGTCCTGCAAGGAAG
CCGGGACGGTCAGGCGCGTCGTCTTCACTTCCTCCGCCGGCACCGTCAACGTGCAGGAGCAGCAACAG
CCCCAGTACGACGAGAGCTCCTGGAGCGACATCGACTTCTGCCGCCGCGTCAAGATGACCGGATGGAT
GTACTTCGTGTCCAAATCTCTGGCTGAGAAGGCAGCGTACGAGTTTGCGAGGGAGAATGGCATCGACC
TCATCAGCATCATCCCGACTCTTGTCGTCGGCCCCTTCATCACCACGACCATGCCGCCCAGCATGATC
ACCGCGTTGTCACTCATCACAGGAAACGAAGCTCACTATTCGATCTTGAAGCAAGTTCAACTGGTTCA
CTTGGACGACCTGTGTGATACCCACGTCTTCCTGTACGAGCACCCCGATGCAAATGGGAGATACATCT
GCTCCTCTCATGACGCCACCATATACGATCTCGCCAAGATGTTCCGAGCGCGATACCCCCAGTACAAC
ATCCCTCAGCAGTTCGAAGGGATCGATGAAACGATCGAACGGGTGCACTTCTCCTCCAAGAAACTCAC
CGACCTGGGTTACAAGTTCCGGTACACGATGGAGGACATGTTCGATGCCGCCATCGAGTCGTGCTGGG
AGAAGCATCTGATACCACTCCGGACAGCAGAGGAACAGTGCAGTGAGGTGGGCAAACCTCTGCCTCTG
GCAACAGAGACACTGAGTGAGTTCAGTGAAGAGAAGGTCCTGATGGCTTAG

>GSMUA_Achr3P31110_001_(MaDFR1)_Protein

MKGPVVVTGASGYIGTWLVMKLLQKGYVVRATVRDPTNQKKIKPLVELPGSVERLTIWRADLEEEGSF
DEAVKGCEGVFHVATPMDFESKDPENEIIKPTVDGVLSIMRSCKEAGTVRRVVFTSSAGTVNVQEQQQ
PQYDESSWSDIDFCRRVKMTGWMYFVSKSLAEKAAYEFARENGIDLISIIPTLVVGPFITTTMPPSMI
TALSLITGNEAHYSILKQVQLVHLDDLCDTHVFLYEHPDANGRYICSSHDATIYDLAKMFRARYPQYN

IPQQFEGIDETIERVHFSSKKLTDLGYKFRYTMEDMFDAAIESCWEKHLIPLRTAEEQCSEVGKPLPL
ATETLSEFSEEKVLMA

>GSMUA_Achr4T10640_001_(MaDFR2)_Genomic

AGGCACTTCAGGGAACACACCTAAAGTTCATACGACTTCCTCTTCTTCGTTCGTTGGAGCTTGAAAAT
TAGATGAGCCACGTTCCAATCTACCACCATCCTCGTTCTTCTTCCTCCTCCACTGAGAGAAAGCGAGA
TGGAACAGCCACCATCTCTCCCTCTGGTTTCAGTCTTTAAATACATCTGAAGGAGTAAGGCTCGGCAT
ATCAGAATCATCAGTTTAATCTCAGAGAGGGTGAGAGAGAGAGAGAGATGAAGGGATCGGTGGTGGTG
ACCGGAGCCTCGGGATACGTCGGATCATGGCTAGTTATGAAGCTTCTGCAGAATGGATACATCGTCAG
GGCCACCGTGAGAGACCCCTGTCAGTATAGTATATTTCTCTTTGCATCTCGCTCCCTTGCACATGAAG
AGATCAAGCAGGAACAGCAGAGACTTGAATGGTTTGTTGTCTTGATGCAGCGAATCAGAAGAAGGTCA
AGCCTCTGCTAGATCTTCCGGGATCGGCTGAGCGGCTCTCGATCTGGAGAGCAGACTTGGACGAGGAA
GGAAGCTTCGCTGAGGTGATCAAGGGATGTGAAGGAGTGTTCCATGTGGCCACCCCCATGGACTTCGA
GTCCAAAGACCCTGAGGTAGCTCCATTAATCCTCTTCTCTTTGCAGCATGCAGCCTCTCTCTCTCTCT
CTCTCTNNNNNNNNNNNNNNNNNNNNNNNNNNNNNNNNNNNNNNNNNNNNNNNNNNNNNNNNNNNNNN
NNNNNNNNNNNNNNNNNNNNNNNNNNNNNNNNNNNNNNNNNNNNNNNNNNNNNNNNNNNNNNNNNNNN
NNNNNNNNNNNNNNNNNNNNNNNNNNNNNNNNNNCTCTCTCTCTCTCTCTCTCTCTCTCTCTCTCTCT
CTCTCTCTCTCTCTATGTATATGCTCAAGTCCAAGTTGCTTGTAGAATGAGATCATCAAGCCCACCGT
TAATGGGGTTTTGAGCATCATGAGGGCCTGCAAGGAAGCCGGCACGGTCAAGCGCGTCGTGTTCACTT
CCTCGGCGGCACCGTCAACGTGCAGGAGCACCAGCAGCCGGAGTACGACGAGAGCTCCTGGAGCAAC
ATGGAGTTCTGCCGCCGCGTCAAGATGACAGGATGGGTACGTACGTATGTCACTCCCATCTTCTGCTT
GTAGCAAAATTTGAATGGGGGAGAATAAAAGGAAATGATCTTTTACTGCAGATGTATTTCGTGTCCAA
AACACTGGCTGAGAAAGCGGCGTGGGAGTTCGCGAAGGAGAACGGCATTCACTTCATCAGCATCATCC
CGACTCTGGTGGTCGGTCCTTTCATCACCACCACCATGCCGCCTAGTATGATCACTGCGCTGTCACTC
ATCACAGGTACTTGCAGAAACATTTAGCAGCGGTACTTTCGACAGGATCATCAGCCCTGCATGTCTTA
GCTGTTGACTGCATCATCCATGCATGCAAGCAGGAAACGAAGCTCACTACTCGATCTTGAGGCAAGTT
CAACTAGTCCACTTGGACGACCTGTGTGACACCCACATCTTCCTGTACGAGCACCCGAACGCACAAGG
AAGATACATCTGCTCCTCCCATGACGCCACCATATACGACCTCGCAAAGATGTTCAAGGAACGATACC
CCCAGTACATCATCCCTCAAAAGCAAGTGCTCTCATACTCACCCAAACCACTTGAGATGCAAGCAAGA
AAAGATCTATCTAAATAAGAAAGCTATTCGTTGTAGGTTTGAAGGGATCGACGAAGACATCTTAAGGG
TGCACTTCTCCTCGAAGAAGCTCATGGAGCTGGGGTACAAGTTCCAGTACACGATGGAGGACATGTTC
GACGAAGCAATCCGGTCGTGCTGCGAGAAGAAGCTGATACCTTTCCGTACAGCCGAAGGCCATGGCAG
CGAGATGGTGAAGAAGAAACCTGTGAACTCAGCATCAGAGAGAGTGAGTGAGTTCAGTGAAAAGGAAG
TCCTGATTGCTTAGGTAGCGATCTCTGCCGTATCAGATTCTTGTGCTGCATCAGCACAGATGCATGAT
TTAGTTTCATTGGCAGAATGGGATTCTGAGTCTCCTTGATGAACTTTTATCTTAAGAAGATTGAGTTG
CTTGTCTTTGCAGACTGCTGATTCCATTCTTTTCTTGATTGGATCGATGAAGACCCATGTCTGAGCTT
TGATCATAGCCTACCGCAATGTTCACCAAAATAGAGACCATAAACAAACGTTTATATATTATACATAT
ATTTTTTATCAGGATTTATCACTAACATTATATATTTATCACTCAGGAAATAATAAATAAGATTTAGT
AATATATCGTACAAAAAAAAAGGCGACAAAAAAAAAACCAAGAAAAAGTCCACAAATTTAATGACAGGG
ACGATGTCCACATCGAGTCAGAGCATAGGCTCCAAAAACCCTGTTTCCTGTGTCGGAGGAGACAGGTG
GTCGCACCTGAACGAGAGAGTTTGATCGGCCTGCGTCGATCGGGTGTACAGTCTCACGCACAAAGCGC
CCACGGTGCACCAATAGCTGGCGTCCAGCTCTAAGTTCACACATTGATGACGACGCCAACCCGACGCG
AGTCGCCCGGCGTTTTCTTCGACCATCGTCATCCGATTCCCAACCCGAGAAGGAGTTCGTCGCGTTGC
AGAAATATGCGAAACGAG

>GSMUA_Achr4T10640_001_(MaDFR2)_Modified CDS

ATGAAGGGATCGGTGGTGGTGACCGGAGCCTCGGGATACGTCGGATCATGGCTAGTTATGAAGCTTCT
GCAGAATGGATACATCGTCAGGGCCACCGTGAGAGACCCCTCGAATCAGAAGAAGGTCAAGCCTCTGC
TAGATCTTCCGGGATCGGCTGAGCGGCTCTCGATCTGGAGAGCAGACTTGGACGAGGAAGGAAGCTTC
GCTGAGGTGATCAAGGGATGTGAAGGAGTGTTCCATGTGGCCACCCCCATGGACTTCGAGTCCAAAGA

CCCTGAGAATGAGATCATCAAGCCCACCGTTAATGGGGTTTTGAGCATCATGAGGGCCTGCAAGGAAG
CCGGCACGGTCAAGCGCGTCGTGTTCACTTCCTCGGCGGGCACCGTCAACGTGCAGGAGCACCAGCAG
CCGGAGTACGACGAGAGCTCCTGGAGCAACATGGAGTTCTGCCGCCGCGTCAAGATGACAGGATGGAT
GTATTTCGTGTCCAAAACACTGGCTGAGAAAGCGGCGTGGGAGTTCGCGAAGGAGAACGGCATTCACT
TCATCAGCATCATCCCGACTCTGGTGGTCGGTCCTTTCATCACCACCACCATGCCGCCTAGTATGATC
ACTGCGCTGTCACTCATCACAGGAAACGAAGCTCACTACTCGATCTTGAGGCAAGTTCAACTAGTCCA
CTTGGACGACCTGTGTGACACCCACATCTTCCTGTACGAGCACCCGAACGCACAAGGAAGATACATCT
GCTCCTCCCATGACGCCACCATATACGACCTCGCAAAGATGTTCAAGGAACGATACCCCCAGTACATC
ATCCCTCAAAAGTTTGAAGGGATCGACGAAGACATCTTAAGGGTGCACTTCTCCTCGAAGAAGCTCAT
GGAGCTGGGGTACAAGTTCCAGTACACGATGGAGGACATGTTCGACGAAGCAATCCGGTCGTGCTGCG
AGAAGAAGCTGATACCTTTCCGTACAGCCGAAGGCCATGGCAGCGAGATGGTGAAGAAGAAACCTGTG
AACTCAGCATCAGAGAGAGTGAGTGAGTTCAGTGAAAAGGAAGTCCTGATTGCTTAG

>GSMUA_Achr4P10640_001_(MaDFR2)_Protein

MKGSVVVTGASGYVGSWLVMKLLQNGYIVRATVRDPSNQKKVKPLLDLPGSAERLSIWRADLDEEGSF
AEVIKGCEGVFHVATPMDFESKDPENEIIKPTVNGVLSIMRACKEAGTVKRVVFTSSAGTVNVQEHQQ
PEYDESSWSNMEFCRRVKMTGWMYFVSKTLAEKAAWEFAKENGIHFISIIPTLVVGPFITTTMPPSMI
TALSLITGNEAHYSILRQVQLVHLDDLCDTHIFLYEHPNAQGRYICSSHDATIYDLAKMFKERYPQYI
IPQKFEGIDEDILRVHFSSKKLMELGYKFQYTMEDMFDEAIRSCCEKKLIPFRTAEGHGSEMVKKKPV
NSASERVSEFSEKEVLIA

>GSMUA_Achr4T24540_001_(MaDFR3)_Genomic

AGAACTGATCAATCAATCGCCTTCCACTGTGGAGCCATCGTGCTCGTCTCCGCCTTGGACAACTGAAT
CAGTAGAAAGCAAAAAGCGGCGACGATAACCTAACCATCTCGTCAAGTGCGGGTAGATGAGAGCACAT
TGTTGTTTGTTAGGTTGCATGCAAACAAAAAAGCACAAGGGGCTCGCATTTGTTATATGTAGCTACGT
AGGTTGAGCAGAGAGATTGCAGTCGTAGAAAAGGTGCATTGCGTCTTACTCCTTTGTTCCATCAACTT
CTTCTTCTGTTCCCATGGCGTACTTCCGCTGTCGCCCGTTGACAACGAGTAACCCCAGCGTGGGCGAG
AAGGGGATGGTGTGCGTGACTGGGGCCGCCGGATTCGTAGGGTCATGGCTCATTATGAGGCTCCTTGA
GCACGGCTACACTGTGAAAGCGACTGTTCGCGATCCGAGTACTGCTTCCCATTGATCTAATTTACTGG
TTAGCTAGTCCTAGTAGAGTTGGTGATGATGCGTGGTTAATTACGTGATCGTCGTGTTCAATAAGTTC
TATAAAGACATGAAAGTGTTTGATCGGATTGTGGTGGTTTGATCTTTGTTGCAGACAACTTGAGGAAG
GTGAAACATCTGCTGGATCTTCCAAAGGCTAGCACCGATCTTACGCTGTGGAAGGCTGACCTCGTTGA
CCAAGGAAGCTTCGATGATGCCGTGCGAGGGTGCGTGGGTGTCTTCCATGTCGCCACTCCAATGGATT
TCCAGTGCTCCGACCCCGAGGTAAGCCCAGACACAACCTACCTATTCTAGTTGACTCATACTGTACAT
CCCATGACTCTTCCGAAGATACAAGACGAGCTTGCAAAGATCTGCCAGTTTAATGGAGTTAGAGAAAG
CAAAAAAGACGATACATCATGCATTTGACATTGGTTTTGGCTTGTTGTTTCAGAACGAAGTAATAAAG
CCAGCGATAGATGGGATGATGAACGTCCTCAAGTCCTGCGCAAAGGCAGGCACAGTGAGGAGGGTGGT
GTTCACGTCGTCTGCTGGAACCACCTGCGTTCATGCTCGTCGCAAGGAGGAGTACGACGAGAACTCAT
GGAGCGATGTCGAGTTCTGCAGGGCTAAGAAGATGACTGGTTGGGTAATCAACCGGACACATACACGG
AATCATCAATTCGCCGACTACGTTTTGACTACAGTAGTGCAAATTAAGCTCTTATCTTTGTCCTGTGG
CCACACATTTTGCAGATGTACTTCGTCTCAAAGACGCTGGCGGAGAAGGCAGCATGGGAATTTGCCGA
GAAGAACCATTTGGATTTCATAAGCATCATACCGACCTTAGTCAACGGACCATTCATCATTCCCACCA
TGCCTCCCAGCATGCTCTCTGCTCTGGCTTTGGTAACAGGTTCGCATGCCCACGTCTTCTACGTAATA
AATCACCAGTTTCCTGCCACCTACATATCATTCGAACAGAAACCAACTCTCTCTCTCTCTTTCTAAAC
TCACTGCCAATGATGATGATGATGGTATAACAATGCTTGGCAGGGAACACACCACATTACTCGATCTT
GAACCCAGTCCAGTTTGTGCACTTGGATGATCTTTGCATGGCTCACATATTCTTGTTCGAGCATCCGG
AAGCGAAGGGGAGATACGTTTGCTCTTCTCACGACATCACCATCTCCGATCTGGCGAAGATGCTAACG
GAGAGGTACCCTGAGTATGACATCCCCACCGAGTAAGTCACCTCCGCGAGAAGGACTGTAAGATCTGG
TGTTGGACTCGAAGAGGGGATTATATATCTACTTTGATTCTCTTGCCATAAGACTGACTGAGGGCTGC
ACCAATTACACGTAGGTTTGAAGGGATCGATGAGGTATCGGACGTGATCAAGTTCTCCTCAAAGAAGC
TTACTGACTTGGGCTTTACGTTCAAGTACAGCATGGAGGACATGTTCGACGGAGCAATAGAGTCGTGC
AGGGAGAAGGGATTGCTGCCGCTGACAACCAAGAAAGAGCAGGTCGATGGCCACAACTAGATTTTACC

AAGGGAATAACACGTCAGATGATCTGTGTTGGAGAACTTCTATCTTCTGTTCATCTTTATTTGGGTCA
TGTATCTCAGAGCAATATAGTCTCTTTGCATCTTCTATCCAGTTAATAAACCGAATCGATGGCTTTAC
TAAAGGTGATATACGATGACAAACAAAAAGAAGGTCAAAAGACAAAAACAAATTAAGCCTTTGGATTG
TGATGA

>GSMUA_Achr4T24540_001_(MaDFR3)_Modified CDS

ATGGCGTACTTCCGCTGTCGCCCGTTGACAACGAGTAACCCCAGCGTGGGCGAGAAGGGGATGGTGTG
CGTGACTGGGGCCGCCGGATTCGTAGGGTCATGGCTCATTATGAGGCTCCTTGAGCACGGCTACACTG
TGAAAGCGACTGTTCGCGATCCGAACAACTTGAGGAAGGTGAAACATCTGCTGGATCTTCCAAAGGCT
AGCACCGATCTTACGCTGTGGAAGGCTGACCTCGTTGACCAAGGAAGCTTCGATGATGCCGTGCGAGG
GTGCGTGGGTGTCTTCCATGTCGCCACTCCAATGGATTTCCAGTGCTCCGACCCCGAGAACGAAGTAA
TAAAGCCAGCGATAGATGGGATGATGAACGTCCTCAAGTCCTGCGCAAAGGCAGGCACAGTGAGGAGG
GTGGTGTTCACGTCGTCTGCTGGAACCACCTGCGTTCATGCTCGTCGCAAGGAGGAGTACGACGAGAA
CTCATGGAGCGATGTCGAGTTCTGCAGGGCTAAGAAGATGACTGGTTGGATGTACTTCGTCTCAAAGA
CGCTGGCGGAGAAGGCAGCATGGGAATTTGCCGAGAAGAACCATTTGGATTTCATAAGCATCATACCG
ACCTTAGTCAACGGACCATTCATCATTCCCACCATGCCTCCCAGCATGCTCTCTGCTCTGGCTTTGGT
AACAGGGAACACACCACATTACTCGATCTTGAACCCAGTCCAGTTTGTGCACTTGGATGATCTTTGCA
TGGCTCACATATTCTTGTTCGAGCATCCGGAAGCGAAGGGGAGATACGTTTGCTCTTCTCACGACATC
ACCATCTCCGATCTGGCGAAGATGCTAACGGAGAGGTACCCTGAGTATGACATCCCCACCGAGTTTGA
AGGGATCGATGAGGTATCGGACGTGATCAAGTTCTCCTCAAAGAAGCTTACTGACTTGGGCTTTACGT
TCAAGTACAGCATGGAGGACATGTTCGACGGAGCAATAGAGTCGTGCAGGGAGAAGGGATTGCTGCCG
CTGACAACCAAGAAAGAGCAGGTCGATGGCCACAACTAG

>GSMUA_Achr4P24540_001_(MaDFR3)_Protein

MAYFRCRPLTTSNPSVGEKGMVCVTGAAGFVGSWLIMRLLEHGYTVKATVRDPNNLRKVKHLLDLPKA
STDLTLWKADLVDQGSFDDAVRGCVGVFHVATPMDFQCSDPENEVIKPAIDGMMNVLKSCAKAGTVRR
VVFTSSAGTTCVHARRKEEYDENSWSDVEFCRAKKMTGWMYFVSKTLAEKAAWEFAEKNHLDFISIIP
TLVNGPFIIPTMPPSMLSALALVTGNTPHYSILNPVQFVHLDDLCMAHIFLFEHPEAKGRYVCSSHDI
TISDLAKMLTERYPEYDIPTEFEGIDEVSDVIKFSSKKLTDLGFTFKYSMEDMFDGAIESCREKGLLP
LTTKKEQVDGHN

>GSMUA_Achr5T04080_001_(MaANS)_Genomic

GCTGCCAGTAGCGGAAGCTTCTCCTATATATGTACTGCAGCCTTCCTCGTTGGGACAGCAGCAGCAAG
CAGCAGCCCTCTTGGAATTGTTCCGACGAGGACCGAGAGAGACCAGATGGCCACCAAGGTCGTGTCTG
TGGCGCCCAGGGTGGAGATCCTTGCAAAGAGCGGCATCAACGAGATCCCGACCGAGTACGTCCGCCCC
GAGTCGGAGCGGCTCGACCTCGGTGACGCGTTCGAGGAGGTGAAGAAGGCGGCGGAGGGGCCTCAGAT
TCCTGTGGTGGACCTCCAGGGTTTCGACTCGCCTGATGAAGAGGTGAGAAGGGCGTGCGTGGAGGAGG
TGAGGAAGGCGGCGACGGAGTGGGGGGTGATGCACATCGTGAATCACGGCATCCCGTTGGAGCTCGTC
GAGCAGCTGAGGAAGGTGGGGAAGGAGTTCTTCGACCTGCCCATAGAGCAGAAGGAGCAGTACGCGAA
CGACCAGTCATCAGGGAAGATCCAAGGGTACGGGAGCAAGCTGGCGAACAACGCGAGTGGGCAGCTCG
AGTGGGAGGACTACTTCTTCCACCTTATATTCCCGGAGGAGAAGATCAACATCTCCATTTGGCCCAAG
CAACCAACCGACTACATGTCGGCTTCCGTCTCCCCGTATCCGTATTCCTATTTTTTCTTCTCGTCGTC
TTCCTTCAAAGGTTTTCTCTATCCGTCGGTGCAGTGAGGTGACGAAGAAGTTCGGGAGGCAGCTGAGG
GCGGTGGTCACCAAGATGCTGGAAGTTCTTTCCCTGGGTCTTGGACTGGAAGAGGGGAAGCTGGACAG
GGAACTCGGAGGGATGGAGGACCTGCTGATGCAGTTGAAGATCAACTACTACCCTATCTGCCCACAGC
CCGATCTCGCCCTCGGCGTCGAGGCGCACACCGACATCAGCGCGCTCTCCTTCATCCTCCACAACATG
GTGCCGGGGCTGCAGATCTACTACGGCGGCAGGTGGGTCACCGCCAAATGCGTGCCGGACTCCATCAT
CATGCACGTCGGAGACTGCCTCGAGATCCTGAGCAATGGGCAGTACAAGAGCATCCTCCACCGCGGGC
TCGTCAACAAGGAGAAGGTGCGCATCTCCTGGGCGGTCTTCTGCGAGCCTCCCAAAGACAAGATCGTG

CTGAAGCCACTGGAGGAGCTCGTGGCCGACGGGACGCCGGCCAAGTTCCCCCCGCGCACCTTCGAGCA
GCACATCCAGCACAAGCTCTTCAAGAAGACTCGGGGGGACTTCAAGACCCCAAACTGAGATCATCGCC
TGTGGTGCAACTGTCCTTTTAGTACATCGTCACGCTTCTACCGTCCCGTGTTAACCACGTTGCTTTCC
GGAGTCCAAGTATGTGATTTAGTCGGTCTTCTCCTTGTAGCACCGTCCTCAAAGGTCCATGGAAATGC
TCTCCAATAATATGCTATGTATGTTCTCTTGTTCAACCTCATGTCTTAGTCTACTTTGAAATCCTACT
CACAATAAACATCATACGATCAGTGGCTGGATGTGTGTTGGAATCTCACTTTGGTCCGCCAAGAACAT
ATCACTAGAGAATGGCCAACACTTTAAAGGAATTTG

>GSMUA_Achr5T04080_001_(MaANS)_Modified CDS

ATGGCCACCAAGGTCGTGTCTGTGGCGCCCAGGGTGGAGATCCTTGCAAAGAGCGGCATCAACGAGAT
CCCGACCGAGTACGTCCGCCCCGAGTCGGAGCGGCTCGACCTCGGTGACGCGTTCGAGGAGGTGAAGA
AGGCGGCGGAGGGGCCTCAGATTCCTGTGGTGGACCTCCAGGGTTTCGACTCGCCTGATGAAGAGGTG
AGAAGGGCGTGCGTGGAGGAGGTGAGGAAGGCGGCGACGGAGTGGGGGGTGATGCACATCGTGAATCA
CGGCATCCCGTTGGAGCTCGTCGAGCAGCTGAGGAAGGTGGGGAAGGAGTTCTTCGACCTGCCCATAG
AGCAGAAGGAGCAGTACGCGAACGACCAGTCATCAGGGAAGATCCAAGGGTACGGGAGCAAGCTGGCG
AACAACGCGAGTGGGCAGCTCGAGTGGGAGGACTACTTCTTCCACCTTATATTCCCGGAGGAGAAGAT
CAACATCTCCATTTGGCCCAAGCAACCAACCGACTACATTGAGGTGACGAAGAAGTTCGGGAGGCAGC
TGAGGGCGGTGGTCACCAAGATGCTGGAAGTTCTTTCCCTGGGTCTTGGACTGGAAGAGGGGAAGCTG
GACAGGGAACTCGGAGGGATGGAGGACCTGCTGATGCAGTTGAAGATCAACTACTACCCTATCTGCCC
ACAGCCCGATCTCGCCCTCGGCGTCGAGGCGCACACCGACATCAGCGCGCTCTCCTTCATCCTCCACA
ACATGGTGCCGGGGCTGCAGATCTACTACGGCGGCAGGTGGGTCACCGCCAAATGCGTGCCGGACTCC
ATCATCATGCACGTCGGAGACTGCCTCGAGATCCTGAGCAATGGGCAGTACAAGAGCATCCTCCACCG
CGGGCTCGTCAACAAGGAGAAGGTGCGCATCTCCTGGGCGGTCTTCTGCGAGCCTCCCAAAGACAAGA
TCGTGCTGAAGCCACTGGAGGAGCTCGTGGCCGACGGGACGCCGGCCAAGTTCCCCCCGCGCACCTTC
GAGCAGCACATCCAGCACAAGCTCTTCAAGAAGACTCGGGGGGACTTCAAGACCCCAAACTGA

>GSMUA_Achr5P04080_001_(MaANS)_Protein

MATKVVSVAPRVEILAKSGINEIPTEYVRPESERLDLGDAFEEVKKAAEGPQIPVVDLQGFDSPDEEV
RRACVEEVRKAATEWGVMHIVNHGIPLELVEQLRKVGKEFFDLPIEQKEQYANDQSSGKIQGYGSKLA
NNASGQLEWEDYFFHLIFPEEKINISIWPKQPTDYIEVTKKFGRQLRAVVTKMLEVLSLGLGLEEGKL
DRELGGMEDLLMQLKINYYPICPQPDLALGVEAHTDISALSFILHNMVPGLQIYYGGRWVTAKCVPDS
IIMHVGDCLEILSNGQYKSILHRGLVNKEKVRISWAVFCEPPKDKIVLKPLEELVADGTPAKFPPRTF
EQHIQHKLFKKTRGDFKTPN

>GSMUA_Achr8T01490_001_(MaANR)_Genomic

ATGACCGGAGCAATATGGATAAGAGTTCCAATTTAGAGGCCCTGTTGAGTCGGAAATAACTCTTGGTG
TTGTGGGGGTGACGAAGAAGCAGCCTTTGAACGGTGGTTAAGCTTTACTGTGGCTTCGTTGGTGAACA
CCCAACGTGATGATCTACCTGCACTTTCTGCGTCGTATTTAACGCCGTGCGAGTGGCCGAGAGGTGCA
CTGTGAGCCAGGATACAGCACTCGAAGAAGCGATCGAGATGGCGAGCCAGGAACAGAAGACTGCCTGC
GTCACCGGCGGCAATGGGTTCATCGCGTCGACGCTCGTCAAGCAGTTGCTGGAGAAGGGCTACGCTGT
CAACGTCACCGTCAGGGATCCAGGTTTCGTCTATGGTGACCACAGCATCCGACATGGCCGCCCCAGTG
TTTGACCGTTTGTGTTGTTGGTGCAGATAATGCGTCCAAGGTTGGTCGCCTGAAGCAGCTGCGGAGTC
TCGGCAGGTTGACCATTTTCCGAGCGGATCTGTCGGAGGAGGGCAGCTTCGATGCGGCGGTGAGCGGC
TGCCACTACGTCTTCCTCGTCGCAGCTCCGGTGAACATGGCGGCCGAGGACGCTGAGGTCGGCCCTCA
ACCACTCGATCACGAGACTGCTGCGTGTCTTTTTGCTGCTCATCAGTTCTTTGTCTCTTCTTCTGATT
TCGACGTCAGAATGAGCTGATCAAACCGGCGATCCAAGGAACCCTGAACTTGTTGAGATCGTGCGTCA
AGGCCAAGACGGTCGAGCGCGTCGTCCTGACGTCGTCGGCGGCCAGCGTCTCCATCAACGAGCTCAAA
GGAACTGGTATGGTGTTGGATGAGGAGGCCTGGTCGGACCTCACATACTTAACGTCCAAGAAACCCCC
TAGTTGGGTAAGCCATCGCGAGTTGCACGAACCTTGCTCGTTCTCAACTCACGGTAACAAGCAGCAGC

ACCACCGTGCAGGGATATGCGGTCTCCAAGGTGCTGGCGGAGAAGGAAGCATCCAAGTTTGCACTAGA
GAACGGCATAGATCTCGTGACGATGATCCCAGCCCTGACGATTGGCCCTGCGCTGGGCGCAGAGGTTA
ATCTGAGCTTAATGCTCGGCGTGTCTTTGCTATCAGGTAAGGAAGAGCGAAGTGGAAGAGACGGTGAA
TCGAAGAAACTGATATGAGAACTCGGCCTTAATTTTGGTGTCCACAGGCAACGAAGAGCTGATCGACG
GCTTCAGAATCATGCAAACCCTCTCCGGTTCCATCTCGTTCACCCACGTCGAGGACATTTGCAGGGCT
CACATCTTTGTTGCGGAGACCGAATCGGCTTCCGGCCGCTACATCTGCTGCTCCATCAACACGAGTCT
CCCGGAGCTTGCCAAGTTCCTCTCAGAACGATACCCTCGGTACAAGGTCCCTACAGAGTAAGCAAAAC
CCTGCAGCTCCTGCAATCAAAAGCATATTCTTGAAATCCAATTGCTAACTTGTGTGGCACGAAATCAA
TCGCAGCTTCTCTGACTTGCCCGAGAAGCCCAAGCTGATGCTGTCTTCGGAGAAGCTCATCAAAGCTG
GTTTTGAGTTCAAGTACAAGCAACTGGAAGACATCTACGACGATACTGTTCAGTTTGCAGAGGCTGTA
GGACTGCTGCAGCCGAAGTCGAAAGGTTCTCATCATCAAACCACAACTGGGAAGGGAGCATGAGAGGT
TTGCGTAGTAACTCGAGAGGCATCTTTCTTTAGGGTACTCAAATAAAAGCCATGCTTGCTATGGTAGA
TGTGGTTTTTCCCTCCGAACCAGGCAGTGTTGGTCTTCTTCCAATGCCATGTTCTCGAGATGAACCTC
GGCGGTGAGAATACCGTAAGCAGCAAGGCATGTTGTTGTCACTCCTATTATGCTATATAGTGAAGCTT
GCCTGAAATTTCAATTAATATTGCTGCGCAGAAATTTTAGTATATT

>GSMUA_Achr8T01490_001_(MaANR)_Modified CDS

ATGGCGAGCCAGGAACAGAAGACTGCCTGCGTCACCGGCGGCAATGGGTTCATCGCGTCGACGCTCGT
CAAGCAGTTGCTGGAGAAGGGCTACGCTGTCAACGTCACCGTCAGGGATCCAGATAATGCGTCCAAGG
TTGGTCGCCTGAAGCAGCTGCGGAGTCTCGGCAGGTTGACCATTTTCCGAGCGGATCTGTCGGAGGAG
GGCAGCTTCGATGCGGCGGTGAGCGGCTGCCACTACGTCTTCCTCGTCGCAGCTCCGGTGAACATGGC
GGCCGAGGACGCTGAGAATGAGCTGATCAAACCGGCGATCCAAGGAACCCTGAACTTGTTGAGATCGT
GCGTCAAGGCCAAGACGGTCGAGCGCGTCGTCCTGACGTCGTCGGCGGCCAGCGTCTCCATCAACGAG
CTCAAAGGAACTGGTATGGTGTTGGATGAGGAGGCCTGGTCGGACCTCACATACTTAACGTCCAAGAA
ACCCCCTAGTTGGGGATATGCGGTCTCCAAGGTGCTGGCGGAGAAGGAAGCATCCAAGTTTGCACTAG
AGAACGGCATAGATCTCGTGACGATGATCCCAGCCCTGACGATTGGCCCTGCGCTGGGCGCAGAGGTT
AATCTGAGCTTAATGCTCGGCGTGTCTTTGCTATCAGGTAAGGAAGAGCGAAAGCTGATCGACGGCTT
CAGAATCATGCAAACCCTCTCCGGTTCCATCTCGTTCACCCACGTCGAGGACATTTGCAGGGCTCACA
TCTTTGTTGCGGAGACCGAATCGGCTTCCGGCCGCTACATCTGCTGCTCCATCAACACGAGTCTCCCG
GAGCTTGCCAAGTTCCTCTCAGAACGATACCCTCGGTACAAGGTCCCTACAGACTTCTCTGACTTGCC
CGAGAAGCCCAAGCTGATGCTGTCTTCGGAGAAGCTCATCAAAGCTGGTTTTGAGTTCAAGTACAAGC
AACTGGAAGACATCTACGACGATACTGTTCAGTTTGCAGAGGCTGTAGGACTGCTGCAGCCGAAGTCG
AAAGGTTCTCATCATCAAACCACAACTGGGAAGGGAGCATGA

>GSMUA_Achr8P01490_001_(MaANR)_Protein

MASQEQKTACVTGGNGFIASTLVKQLLEKGYAVNVTVRDPDNASKVGRLKQLRSLGRLTIFRADLSEE
GSFDAAVSGCHYVFLVAAPVNMAAEDAENELIKPAIQGTLNLLRSCVKAKTVERVVLTSSAASVSINE
LKGTGMVLDEEAWSDLTYLTSKKPPSWGYAVSKVLAEKEASKFALENGIDLVTMIPALTIGPALGAEV
NLSLMLGVSLLSGKEERKLIDGFRIMQTLSGSISFTHVEDICRAHIFVAETESASGRYICCSINTSLP
ELAKFLSERYPRYKVPTDFSDLPEKPKLMLSSEKLIKAGFEFKYKQLEDIYDDTVQFAEAVGLLQPKS
KGSHHQTTTGKGA

>GSMUA_AchrUn_randomT18370_001_(MaLAR)_Genomic

CATACATTACATGATGAAGGCTGTACTGTACGAGGTAATGCAAAGCAAAGTGCACGATAGGCGATCCT
TGACGGTTAGGTGCAATCAAGACCGTCGGAGGGATGGATCCTGTACGTCTCCCCATGTGCATGTGGCT
TTATATGATCAGCCCGGGCGGCCGCTTCGCTTTGCCCACTGCTCCTTCCCCACCCTCTAATTCTTTCA
GCATATTCTCCCCGCCATGGCTTCCACCGCCGCTGGTCATGAGCTCGCGCCCACCACGGGAGCCACCC
TCGTCGTGGGTGGCACCGGCTACATCGGCCGCTTCGTCGCCGAGGCTGGCCTGGCCCTTGGCCACGCC
CTCTACGTCCTCGTCCGCACCTTTGGCCCCTTCGTTTCCCCCTCCCGTGCGGCCACCGCCGCAGCCCT

CCGCGAGAAAGGAGCCATTATTTTGGAGGTAATATTGCATGAGCTCTCATCTCGTTTATCGTGTATGT
TAACCTCAATAATGATGATGATGAGGAGGAGGAGGAGGAGGACTTCCGATGTGTTTCAGGGTTCCGTG
GACGATAAAGACTTCATGGAGAAGACTCTGAGAGAGAACAACATCCATGTAGTTATCTCCGCGGTTGG
CGGCGAGAGCATTCTGGATCAGCTCTGCTTGCTCGACGCCATCCAAGCTGCGGGCACCGTCAAGGTAC
GTGTTACACAACGAGTACTGCTGCTATTGTTTTGCATGGACAAGGGCGTCATGCATGGGCGATAGCTA
TGCCTTGCGCTGGTGTGGCAGCGGTTCCTGCCGTCGGAGTTCGGGCACGACATCGACAAGGCGAACCC
GGTGGAGCCGGCACTGAGCCTCTACAACAGAAAGAGAAGGGTTCGGCGAGCCGTCGAGGCCGCCGGCG
TTCCCTACACCTACATATGCTGCAACTCCATCGCCGGCTGGCCCTACCACGACAACAAACACCCGTCC
AAGGCGGCGCCACCGCTGGACCGCTTCGTCATCTATGGGGACGGCAGCGTCAGAGGTAAATCTCACTG
CCGACATGCATGAATAATTAGGCAGCGTGGACACCAAACTGCGACTTCAGGATCAATAAAAAATAAGA
TATTTCAAGTACCACATTAACATACTAATAATAAGACGACGTCCTTCTAAATCTAAATAAAGGAACGT
ACATATACAATTTATATAACCAACCCTTGGATCACTGAATTTTTAATAGTGGATCAATCAATAATATT
TAAAGAATAAAAACTAATTATGTAATATAAATAAATAAATTTAAAATATTAAGATTTATGTCTTAATC
CCAAGGTCTAGAGCTCGTTTGCGTGTTTCTTTAATTATTATTATTATTATTATTATTATTATTATTAT
TATTTAATGTTGATGACGTTGCAGAAGACAAATATTACATTGACCAACAAATAAAAAATCCTGAAAAA
CTATTGTTACTGTTACATTGAAGTGTAGGGTGTTGATGGATGCTTGATGTCGCGTTGGTTGTGTTGTA
GCCTTCTTTGTTGCAGGAAGTGACATTGGGAAATTTACGATGAGATCAGCCTTCGACAGTCGCACCGT
GAATAGAGCTGTTCACTTCCGCCCGACTTGCAATTGTGTTAGCTTGAACGAGATGGCTTCCCTGTGGG
AGCGCAAGATTGGAAGGACACTGCCAAGAGTCAGATTCACTGAGGAGGACCTGCTCGCCATTGCTAAA
GGTTGGAACCTTGGATCATGCTTTTGAGTTCCTTCTGACTGAATAAAGATTTTGAATTGGATCATCAC
TAATCTTAAACTTGAACCCAAGCGTATGATACAAGTCCAAATGAGACAAAAAAAAAAAAAAAAAAGAG
GAGAAATATGGGATTCGAAAGTATCGTGATAAAGAATGAAGCTTTCCGGATTAGATATCACCCAATTA
CAATCATTATAATTCATCCGATGTTCGACTGATCCGGTGGTACTTTATTATTATTCCTCATGTTGGCT
TATGCTAAGCATACTTCCACTTTCACTTGATGGAGAATGTTAATGTTTAGAATAAAGCGACCGAGATC
TTTTCGCATAGCTTAAGCTTTTTGGAATAAATGGTCAAATATTTATATACCATGATAAATTGATCAGA
TGACCATTATCATATATTAAAAAAAAGGTTTAAGAATTTTGGATAAATAATAACGAAGTTTTAAATGA
AAATTTATTAATCACTAATGATCTCAAAATCAAAATTTTTACATGGTATTTGAGGTCAGGTAATAGAT
TCATGCACATGATTCGACATGAAGAAAGTATCAATGGTTGCTACTTGGATCATCGGACACCATAAACA
TCCCTCTGCATTCCGACCATGAAAAGTTCATTTTATATAGGCAAATCGTAGATCTCAGCTTTTTTCAT
TCCTTTACATATACTGAAATTTCCAACACTAGCAAAAATAGAATGAAATCCATTGCATAAGATTCTCA
ACAATATTAATATCTATTGACACCAAGACTCCCAACAATAAAAATGATCTTTTGCTGCACACAAGGAA
CTGTAGTTATAACTTTTTTGGATGAAAATTTTCATCTACCAGTATAGTTAATGAACGGTATTAGTATA
TTCTACGACACCTATCACGACAATTATTAACCACAGGTATAGATATTATGACTACAGTTGTAAACCGT
TATCTACACCTAAAAGTATTGTAGTGACTTCTTACGCTGCGTCACTAATAAATCAGTGAGATTTTTTG
AGAAAAATACTGCTCTTCTCTCTCTCTCTCTCTCTCTCTCTCTATGTATACAATAAATTGTATGAT
TGTTCGTTTACGATGGAAGCTCTTATATTTCCTGTTGGGTTGGTGCAGAGAATGAGATTCCAGCCAGC
GTTGTTGCTGCCTTGACTCATGACATCTTCATCCTTGGATGCCAATCCAATTACTCGGTGGACGGGGT
GAAGGATGTGGAGGTCAGCTGCCTCTACCCCGACATGGCATTCCGGACACTCGATGACTGCTTCAGTG
ACTACATCTCGAGCCTTTTGCCCCACCAAGCTAGGGAGACAAGACCATCTGCGGCCGCTGCTACGGTG
GATCAACCGGCAGTTCCACCGGCCACTGCTTAATTACAAGCATGCATGGATGCATAGAACTCCATCGG
ACCTCTTCCATCATAGAATAATCTTTTATGTGATGTAATGAGATATAAAGAGATATAACTTTTGTATA
TGAATAAATACTAGCAGATCATAACATGCAGCGAAATTAAATAGAATTATAATCAAATAAAATATCAA
GATATATGTGAAAAATCTCTCCAATG

>GSMUA_AchrUn_randomT18370_001_(MaLAR)_Modified CDS

ATGGCTTCCACCGCCGCTGGTCATGAGCTCGCGCCCACCACGGGAGCCACCCTCGTCGTGGGTGGCAC
CGGCTACATCGGCCGCTTCGTCGCCGAGGCTGGCCTGGCCCTTGGCCACGCCCTCTACGTCCTCGTCC
GCACCTTTGGCCCCTTCGTTTCCCCCTCCCGTGCGGCCACCGCCGCAGCCCTCCGCGAGAAAGGAGCC
ATTATTTTGGAGGGTTCCGTGGACGATAAAGACTTCATGGAGAAGACTCTGAGAGAGAACAACATCCA
TGTAGTTATCTCCGCGGTTGGCGGCGAGAGCATTCTGGATCAGCTCTGCTTGCTCGACGCCATCCAAG
CTGCGGGCACCGTCAAGCGGTTCCTGCCGTCGGAGTTCGGGCACGACATCGACAAGGCGAACCCGGTG
GAGCCGGCACTGAGCCTCTACAACAGAAAGAGAAGGGTTCGGCGAGCCGTCGAGGCCGCCGGCGTTCC
CTACACCTACATATGCTGCAACTCCATCGCCGGCTGGCCCTACCACGACAACAAACACCCGTCCAAGG
CGGCGCCACCGCTGGACCGCTTCGTCATCTATGGGGACGGCAGCGTCAGAGCCTTCTTTGTTGCAGGA

AGTGACATTGGGAAATTTACGATGAGATCAGCCTTCGACAGTCGCACCGTGAATAGAGCTGTTCACTT
CCGCCCGACTTGCAATTGTGTTAGCTTGAACGAGATGGCTTCCCTGTGGGAGCGCAAGATTGGAAGGA
CACTGCCAAGAGTCAGATTCACTGAGGAGGACCTGCTCGCCATTGCTAAAGAGAATGAGATTCCAGCC
AGCGTTGTTGCTGCCTTGACTCATGACATCTTCATCCTTGGATGCCAATCCAATTACTCGGTGGACGG
GGTGAAGGATGTGGAGGTCAGCTGCCTCTACCCCGACATGGCATTCCGGACACTCGATGACTGCTTCA
GTGACTACATCTCGAGCCTTTTGCCCCACCAAGCTAGGGAGACAAGACCATCTGCGGCCGCTGCTACG
GTGGATCAACCGGCAGTTCCACCGGCCACTGCTTAA

>GSMUA_AchrUn_randomP18370_001_(MaLAR)_Protein

MASTAAGHELAPTTGATLVVGGTGYIGRFVAEAGLALGHALYVLVRTFGPFVSPSRAATAAALREKGA
IILEGSVDDKDFMEKTLRENNIHVVISAVGGESILDQLCLLDAIQAAGTVKRFLPSEFGHDIDKANPV
EPALSLYNRKRRVRRAVEAAGVPYTYICCNSIAGWPYHDNKHPSKAAPPLDRFVIYGDGSVRAFFVAG
SDIGKFTMRSAFDSRTVNRAVHFRPTCNCVSLNEMASLWERKIGRTLPRVRFTEEDLLAIAKENEIPA
SVVAALTHDIFILGCQSNYSVDGVKDVEVSCLYPDMAFRTLDDCFSDYISSLLPHQARETRPSAAAAT
VDQPAVPPATA
