# Supplementary material for: Genome-wide Expression Analysis and Metabolite Profiling Elucidate Transcriptional Regulation of Flavonoid Biosynthesis and Modulation under Abiotic Stresses in Banana
Source: Sci Rep. 2016 Aug 19;6:31361. doi: 10.1038/srep31361 (PMC4990921; doi:10.1038/srep31361)
Supplement: Supplementary Information [file srep31361-s2.pdf]

Supplementary Information

Genome-wide expression analysis and Metabolite Profiling Elucidate Transcriptional Regulation of Flavonoid Biosynthesis and Modulation under Abiotic Stresses in Banana

Ashutosh Pandey[1,2,*], Anshu Alok[2], Deepika Lakhwani[1], Jagdeep Singh[2], Mehar H. Asif[1] & Prabodh K. Trivedi[1,*]

[1]CSIR-National Botanical Research Institute, Council of Scientific and Industrial Research (CSIR-NBRI), Rana Pratap Marg, Lucknow-226001, INDIA

[2]National Agri-Food Biotechnology Institute (NABI), (Department of Biotechnology, Government of India), C-127, Industrial Area, Phase VIII, S.A.S. Nagar, Mohali 160071, India

[*]Authors for correspondence:

PKT: prabodht@nbri.res.in; prabodht@hotmail.com

AP: ashutosh_biotech@yahoo.co.in

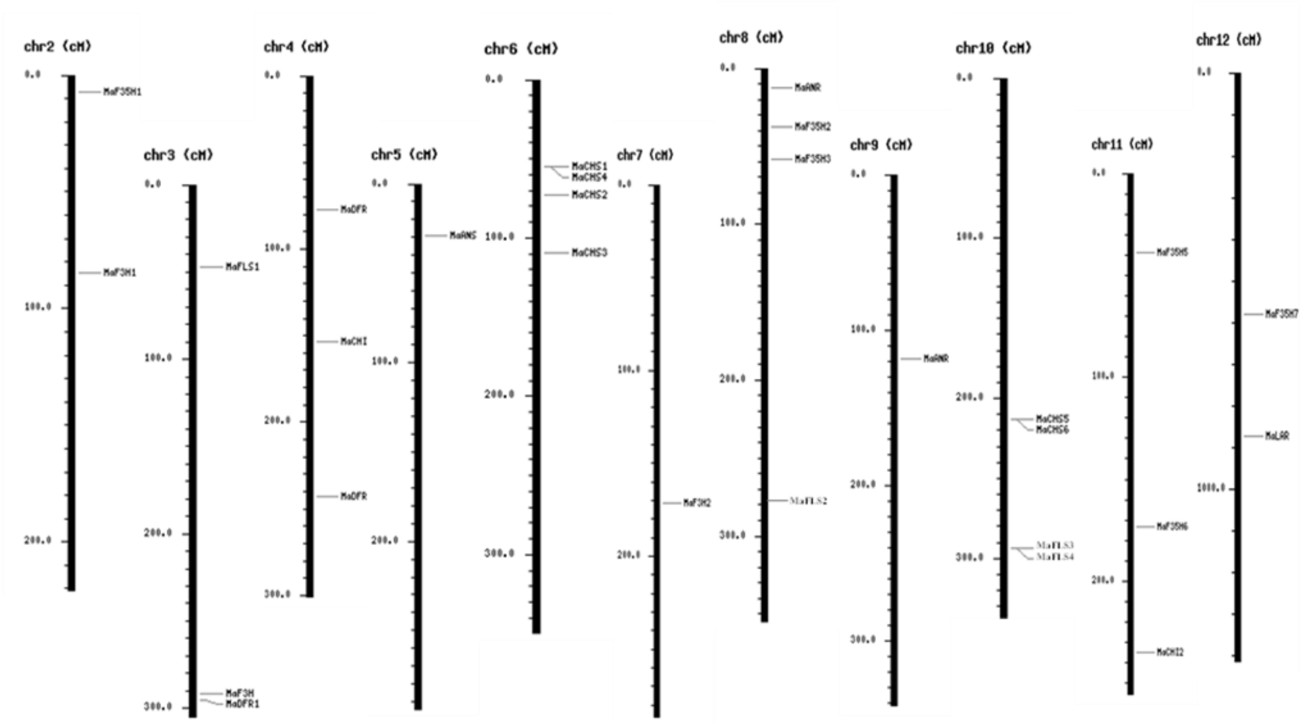

Supplementary Figure S1. Chromosomal localization of different biosynthetic genes in *Musa* genome. The black lines on the chromosomes (vertical) indicate the positions of the respective genes. Numbers represent nucleotide base pair positions.

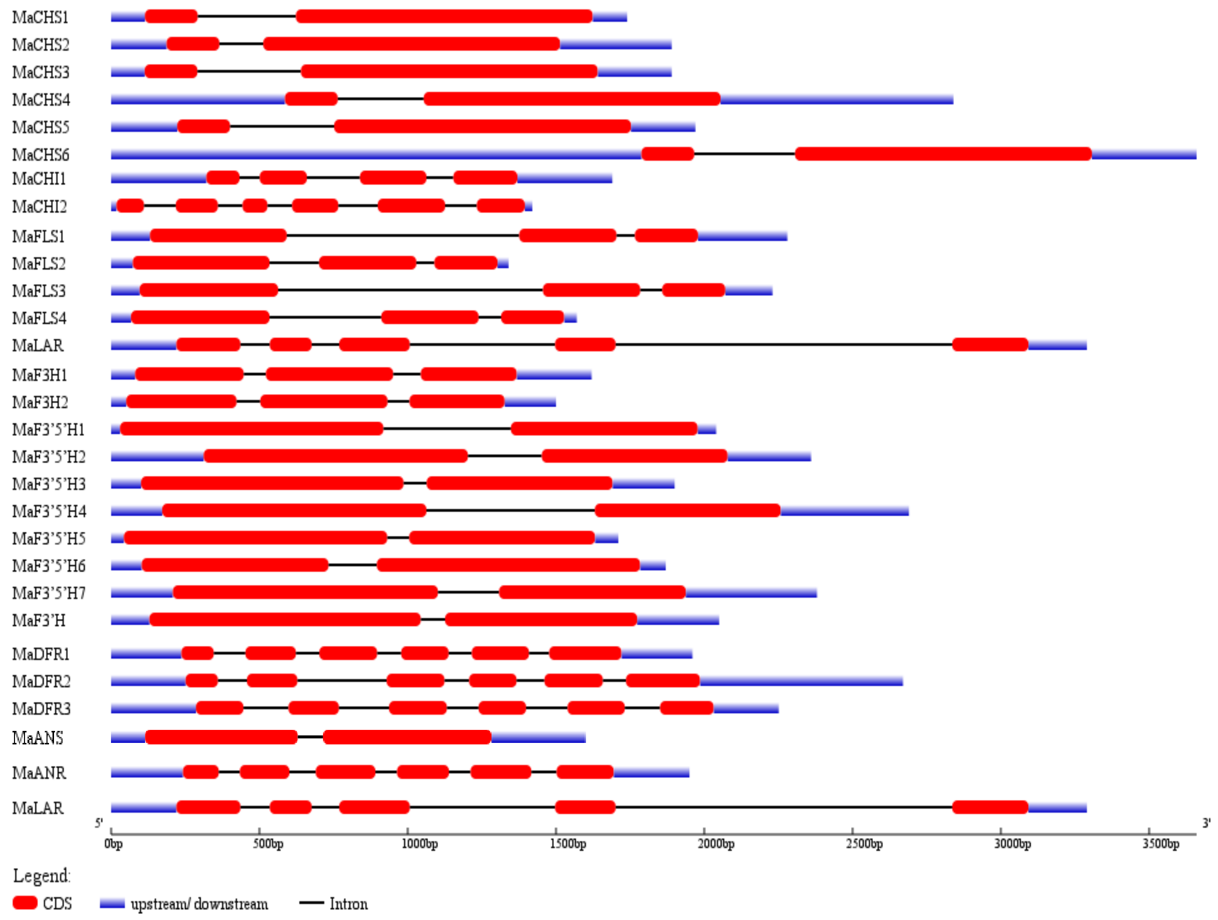

Supplementary Figure S2. Schematic representation of intron- exon composition of flavonoid biosynthetic genes of banana. Genes showing exons (in red boxes) and introns (in black lines).

Table S1. List of the primers used in the present study.

Primer name	Forward Sequence (5' to 3')	Reverse Sequence (5' to 3')
MaCHS1	CAGCCCTTCCATTAACCGGTTCACG	GAGCTCGGAACAGACCACGAGC
MaCHS2	CCACAGTCACCTTCCGCGGG	ACGAGCTGAAAGAGGGGTCGCTC
MaCHS3	CCTGTTCCAGATCGTCTCCGCC	CGCCTCCACCAGGCTCTTCTCTAT
MaCHS4	GACGCGGCAGGTGCTGAGCG	TACCGTCTCCACCGTCAGCCC
MaCHS5	CCCGACCCCGCCACAGAGAA	CCTCCGCCAGGCTCCGCTC
MaCHS5	GCCTCCTCATCTCCGGACCG	GACGTTGACAGGGGTGGCGGT
MaCHI1	CTGCTGAAGCCACTGACCGGG	AGGTCTCGGCCCTGAAAGCCT
MaCHI2	CCAAGTCCCATTCGCTCCTGAGA	TGCCCTTCCAATCCGCTAAATGC
MaF3'H	CGGTGAAGGCGATGGCGGAA	GAGCTCCCGCTGGGCTTGC
MaF3'5'H1	ATGGGGTGTCGCTTTCTGATGT	TTCCCGATTACTTGGTCCATCTC
MaF3'5'H2	CGTCAACGTCAAGGCTATCATC	GTAGTCGATCTCGGATTGCAG
MaF3'5'H3	AAAGCGTCGGGACGTAAGCG	GATGTTCACCTGCCCCGCCAAA
MaF3'5'H4	TCAGTACCTGTTGGGCATGCTA	GCGGGCGCAGAAAAACTTTGA
MaF3'5'H5	ATGCATCGGGGCGGAAGGGA	GTTCCACCACCATGTCCTTGAA
MaF3'5'H6	CGCCATCCTCTCCGAGTTCGT	AGCGACGAACATGTCGAAGATG
MaF3'5'H7	CTGCTCATGGTACAGTACGTGC	ATCGCCTTAAGGGACACCTTC
MaF3H1	CAAACTACAGCAGGCTCTCC	CTTAGTCTTCTCCAGTAGCTCC
MaF3H2	GATCGTGTATCCCCTGGCGA	TGGCATTCTCCGGCAACTCCA
MaFLS1	CGCCGGGGAGCCTCCAGG	TGGATTCTCCGGCCATGCGC
MaFLS2	GGATCCTCGTTCCGGGAGC	CCTCCCTATACGAAGAAGGCTG
MaFLS3	AATTCAAACCGGGGAGCTCGG	CGTTTGCTTTCCTGTAATCAGAA
MaFLS4	CCGGATCGGGGAGCTTGGAA	TGCTCGTTTACTTGCCTATAATC
MaANS	GAGGGGAAGCTGGACAGGGAAC	GTTGTGGAGGATGAAGGAGAGC
MaDFR1	TCGAACGGGTGCACTTCTCCTCC	CCTCACTGCACTGTTCCTCTGCTG
MaDFR2	AGACATCTTAAGGGTGCACTTCTCC	CACAGGTTTCTTCTTCACCATCTCG
MaDFR3	GGTACCCTGAGTATGACATCCCCA	TGCACGACTCTATTGCTCCGTCG
MaANR	CCGGTTCCATCTCGTTCACCCAC	CCGAGGGTATCGTTCTGAGAGGA
MaLAR	GCATTCTGGATCAGCTCTGCTTGC	CTCGCCGAACCCTTCTCTTTCTG
MaActin	ATGACATGGAGAAGATCTGGCATCA	AGCCTGGATGGCAACATACATAGC

Table S2. Quantification of different group of flavonoids (mean value ± standard deviation μg/100g DW) in various tissues of banana

Tissues	Isoquercetin	Quercetin	Kaempferol	Quercetin 3- *O* -galactoside	Rutin	Naringenin	Catechin	Epicatechin
YL	221.708±1.87	244.77±2.64	360.53±1.98	213.73±2.12	2700.02±12.75	50.62±0.86	7085.93±763	ND
BRT	486±5.86	840.5±12.85	53.98±0.87	140.85±1.87	117.3±0.86	10.21±0.15	860.71±56	ND
P.STEM	50.6±0.75	200.13±1.97	5.27±0.02	88.43±0.97	990.71±8.76	2.24±0.01	2325.07±653	ND
ROOT	10.28±0.14	70.5±0.75	ND	ND	351.75±6.86	ND	2799.83±563	ND
UR_PL	246.85±12.12	1811.77±47.83	595.61±17.55	321.69±15.89	14310.37±513.07	58.63±5.44	12742.37±418.34	7816.92±141.82
UR_PP	ND	11.15±1.28	130.86±7.81	2.09±0.23	248.49±21.1	3.91±0.39	167.33±23.83	106.72±15.48
R_PL	211.06±12.96	1696.43±47.89	319.97±28.55	298.58±10.26	9145.01±588.3	49.96±5.32	5655.28±414.61	3769.56±606.87
R_PP	ND	11.08±1.52	111.65±9.59	5.45±0.71	141.82±10.82	5.86±0.7	94.66±18.62	51.4±7.01

Three biological and three technical replicates were used for analysis.

Table S3. Quantification of different group of flavonoids flavonoids (mean value ± standard deviation µg/100g DW) in different developmental stages of fruit pulp of banana

	Isoquercetin	Quercetin	Kaempferol	Quercetin 3- *O* -galactoside	Rutin	Naringenin	Catechin	Epicatechin
6W-Pulp	ND	28.72±1.59	119.49±8.98	7.26±0.58	519.89±13.91	0.61±0.03	406.07±17.16	253.31±20.41
9W-Pulp	ND	33.06±1.85	115.72±6.05	3.3±0.42	151.49±12.71	5.47±0.49	236.55±11.49	179.32±12.44
12W-Pulp	ND	11.26±0.38	275.25±14.94	4.38±0.65	143.1±7.42	9.31±0.66	262.54±23.67	196.17±15.69
15W-Pulp	ND	8.71±0.77	110.7±4.99	4.19±0.36	111.21±4.94	5.91±0.31	138.02±23.57	168.26±14.94
18W-Pulp	ND	11.15±1.28	130.86±7.81	2.09±0.23	248.49±21.1	3.91±0.39	167.33±23.83	106.72±15.48
21W-Pulp	ND	8.6±0.48	242.53±8.68	3.05±0.48	278.19±31.32	2.82±0.28	274.03±20.17	142.22±21.32
24W-Pulp	ND	15.52±1.1	215.06±19.11	6.58±0.83	120.79±5.84	4.4±0.13	125.91±16.75	82.05±11.59
Ripe-Pulp	ND	11.08±1.52	111.65±9.59	5.45±0.71	141.82±10.82	5.86±0.7	94.66±18.62	51.4±7.01

Three biological and three technical replicates were used for analysis.

Table S4. Quantification of different group of flavonoids (mean value ± standard deviation µg/100g DW) in different developmental stages of fruit peel of banana

	Isoquercetin	Quercetin	Kaempferol	Quercetin 3- *O* -galactoside	Rutin	Naringenin	Catechin	Epicatechin
6W-Peel	564.88±44.49	416.1±40.59	397.4±25.53	612.62±20.73	15194.79±540.66	266.12±12.79	14363.32±800.77	6009.32±517.33
9W-Peel	189.83±18.34	461.32±53.85	310.95±27.92	205.05±15.01	12161.33±494.03	47.1±9.98	13253.52±559.01	9821.33±384.44
12W-Peel	419.79±38.84	2025.07±86.85	400.64±26.17	352.28±13.94	14282.38±570.34	46.38±5.77	6342.71±434.2	4248.26±271.39
15W-Peel	272.71±14.16	1778.25±95.55	427.49±42.89	315.02±20.44	14667.13±534.55	39.24±5.52	2410.01±201.89	1590.81±163.94
18W-Peel	246.85±11.41	1811.77±47.83	595.61±17.55	321.69±15.89	14310.37±513.07	58.63±5.44	12742.73±418.34	7816.92±141.82
21W-Peel	227.03±14.68	1655.2±70.11	179.77±13.28	270.19±12.38	15183.33±507.11	28.14±3.12	4941.73±527.22	3253.21±309.06
24W-Peel	244.11±14.25	1794.24±95.05	116.73±11.8	284±10.73	13496.52±556.97	29.29±1.99	8526.26±221.87	6006.4±245.13
Ripe-Peel	211.06±23.1	1696.43±47.89	319.98±28.55	298.58±10.26	9145.01±588.3	49.97±5.32	5655.28±414.61	3769.56±606.87
